# New insights in osteogenic differentiation revealed by mass spectrometric assessment of phosphorylated substrates in murine skin mesenchymal cells

**DOI:** 10.1186/1471-2121-14-47

**Published:** 2013-10-22

**Authors:** Erik Halcsik, Maria Fernanda Forni, Andre Fujita, Thiago Verano-Braga, Ole Nørregaard Jensen, Mari Cleide Sogayar

**Affiliations:** 1 Chemistry Institute, Department of Biochemistry, Cell and Molecular Therapy Center (NUCEL/NETCEM), School of Medicine, University of São Paulo, São Paulo 05508-000, SP, Brazil; 2 Department of Computer Science, Institute of Mathematics and Statistics, University of São Paulo, Rua do Matão 1010, São Paulo, SP 05508-090, Brazil; 3 Department of Biochemistry and Molecular Biology, University of Southern Denmark, Odense, Denmark

## Abstract

**Background:**

Bone fractures and loss represent significant costs for the public health system and often affect the patients quality of life, therefore, understanding the molecular basis for bone regeneration is essential. Cytokines, such as IL-6, IL-10 and TNFα, secreted by inflammatory cells at the lesion site, at the very beginning of the repair process, act as chemotactic factors for mesenchymal stem cells, which proliferate and differentiate into osteoblasts through the autocrine and paracrine action of bone morphogenetic proteins (BMPs), mainly BMP-2. Although it is known that BMP-2 binds to ActRI/BMPR and activates the SMAD 1/5/8 downstream effectors, little is known about the intracellular mechanisms participating in osteoblastic differentiation. We assessed differences in the phosphorylation status of different cellular proteins upon BMP-2 osteogenic induction of isolated murine skin mesenchymal stem cells using Triplex Stable Isotope Dimethyl Labeling coupled with LC/MS.

**Results:**

From 150 μg of starting material, 2,264 proteins were identified and quantified at five different time points, 235 of which are differentially phosphorylated. Kinase motif analysis showed that several substrates display phosphorylation sites for Casein Kinase, p38, CDK and JNK. Gene ontology analysis showed an increase in biological processes related with signaling and differentiation at early time points after BMP2 induction. Moreover, proteins involved in cytoskeleton rearrangement, Wnt and Ras pathways were found to be differentially phosphorylated during all timepoints studied.

**Conclusions:**

Taken together, these data, allow new insights on the intracellular substrates which are phosphorylated early on during differentiation to BMP2-driven osteoblastic differentiation of skin-derived mesenchymal stem cells.

## Background

Fractures and bone loss impose high costs for the Public Healthcare System. Furthermore, delayed-healing fractures lead to recurrence lesion, with quality of life’s loss and increased patient morbidity. In the normal healing process, the bone tissue function is regenerated through endochondral ossification and intramembranous ossification, which often occur at same time at the lesion site, under the influence of inflammatory agents, such as IL1, IL6 and TNF-α [1,2], which induce migration and proliferation of periosteum mesenchymal stem cells. These cells differentiate into osteoblasts, the major step in the regenerative process. However, during the individual’s lifetime, both the availability and the ability of these cells to differentiate diminish, leading to incomplete or total absence of tissue regeneration at the fracture site. Although physiological details are well understood, the molecular aspects of the differentiation process occurring in the osteoblast lineage from adjacent mesenchymal cells remain unclear.

To address this issue, autologous Mesenchymal Stem Cells (MSCs) have been utilized, improving the bone tissue regeneration capability and leading to reduction of both total costs and hospitalization period, with a significant decrease in lesion recurrence [3]. These cells gained importance in Regenerative Medicine, due to their ability to differentiate into chondrocytes, adipocytes and osteoblasts, and facility with which they may be isolated from several organs, among which is the skin. Due to its function of protecting from exposure to deleterious agents, such as UV light, physical injuries and pathogens, the skin displays a high cell proliferation rate, which is maintained by the self-renewal and differentiation capabilities of the several stem cell populations present in skin niches [4]. These cells are of particular interest, since they may be easily isolated from the skin, in reasonable amounts, being highly suitable for bone healing and repair [5].

Although it is known that osteogenic differentiation in MSCs is initiated through activation of canonical pathways such as SMAD (Sma and Mothers Against Decapentaplegic) proteins, the possible protein interactions with other pathways which may influence cell differentiation remain elusive. The activation of different downstream signaling cascade pathways, includes Hedgehog, Wnt, PTHr-P and BMPs, which, in turn, activate the main transcription factors related to osteogenesis through their respective pathways [1]. Smads, for example, may be positively or negatively regulated by phosphorylation of different residues, leading to activation or suppression of the BMP-initiated signal [6]. These kinase pathways, in turn, activate downstream effectors in the cytoplasm and nucleus by phosphorylating a network of substracts. Since the study of protein phosphorylation depends mainly on phosphospecific antibodies and the utilization of radioisotopes, identification of novel phosphorylation sites has been a laborious task. However, the development of mass spectrometry techniques by detection of inorganic phosphate neutral loss through CID (collision induced dissociation), originated from phosphoserine, phosphotyrosine and phosphothreonine, has made it possible to describe these novel phosphorylation sites in several proteins [7,8].

Even though the analysis of fragmented peptides using MS tools is an alternative method, due to their negative charge and low abundance, phosphorylated peptides display poor ionization and are subjected to signal suppression, when compared to the regular, non-modified peptides [7,9]. Therefore, it is necessary to enrich for the phosphorylated peptides population present in the sample and to eliminate interfering ions. This may be accomplished by using a metal affinity chromatography, such as IMAC or TiO_2_, thus improving the detection levels of modified peptides [8,10,11]. This technique, coupled with stable isotope labeling of peptides for quantitative proteomics, may provide information on the proteins which are differentially phosphorylated during BMP2-induced osteodifferentiation. An unexpensive and practical method for quantitative proteomics is the use of stable-isotope dimethyl labeling [12]. Primary amine of tryptic peptides and the lysine ϵ-amino group can react with formaldehyde in the presence of cyanoborohydride through reductive amination, giving rise to dimethylated amine as the product. Depending on which stable isotope is used (^13^C or ^2^H), different shifts in molecular mass may be achieved. Using both non-modified formaldehyde and cyanoborohydride, the mass shift is of 28 Da. Using both deuterated formaldehyde and cyanoborohydride, the mass shift is of 32 Da, and using ^13^C plus deuterated formaldehyde and cyanoborohydride, the mass shift is of 36 Da. Each isotope differs from each other by 4 Da per primary amine labeled, with the comparison between different samples being made by MS precursor ion identification on extracted chromatograms [13].

Here, we employed mass spectrometry coupled to TiO_2_ metal affinity chromatography techniques to uncover new players involved in mouse skin mesenchymal cells (msMSCs) osteogenic differentiation.

## Results

### Quantitative phosphoproteome and proteome of msMSC cells subjected to rhBMP2 osteoblastic differentiation

msMSC cells cultured in 100 mm dishes were treated with rhBMP2 for different periods of time, in order to assess protein phosphorylation changes during the first steps of osteoblastic differentiation. Previous experiments using the osteoblast differentiation medium (ascorbic acid, beta-glycerolphosphate and dexamethasone) showed intense calcification of our murine skin MSCs in 14 and 21 days (Forni et al., personal comunication). Homogeneity of the skin dermal MSCs was probed through a complete characterization of CD markers, namely: CD31, CD90, CD34, CD73 and CD29 (Forni et al., unpublished data), utilizing only cell populations displaying greater than 90% purity for the osteogenic differentiation assays.

**Figure 1:**
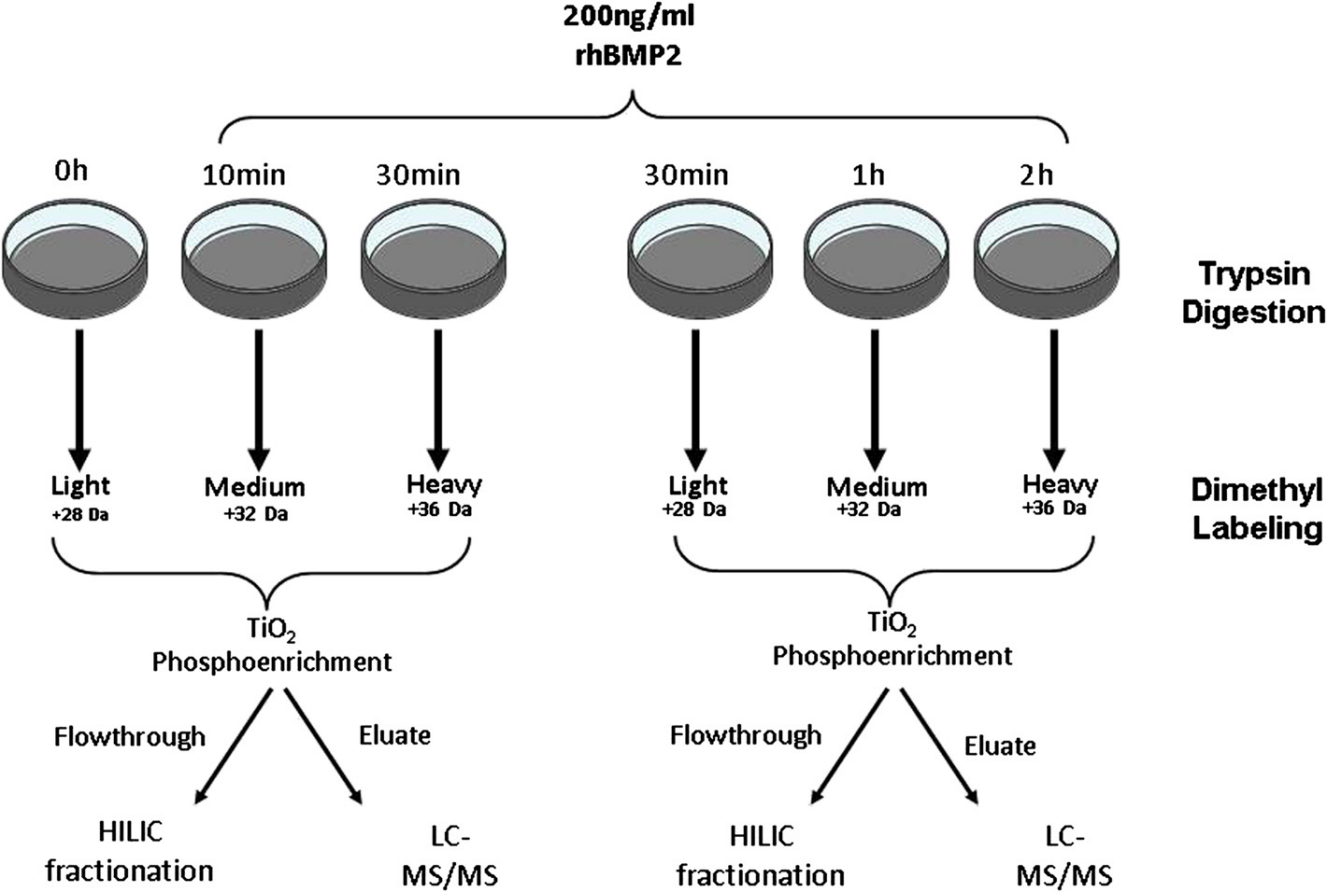
Induction of cells and mass spectrometry experiment outline.

**Figure 2:**
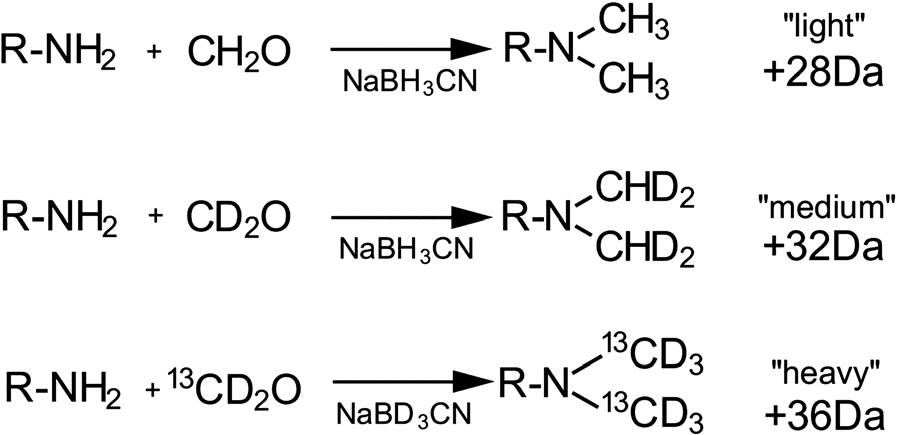
Stable Isotope Dimethyl labeling of tryptic peptides.

Due to the use of three different isotopes to label the samples and five different timepoints, it was necessary to carry out two independent experiments, each of which containing a light, an intermediate and a heavy isotope (Figures 1 and 2). We also evaluated protein level changes, through analysis of the total protein fraction (TiO_2_ flow-through and washed peptides) in order to compare total protein levels alterations. Since the TiO_2_ Flowthrough and Wash fractions represent more than 70% of the sample [14] and are highly complex, another fractionation step was performed. HILIC separation was used to reduce sample complexity, according to protein hydrophilicity.

### Data analysis

**Figure 3:**
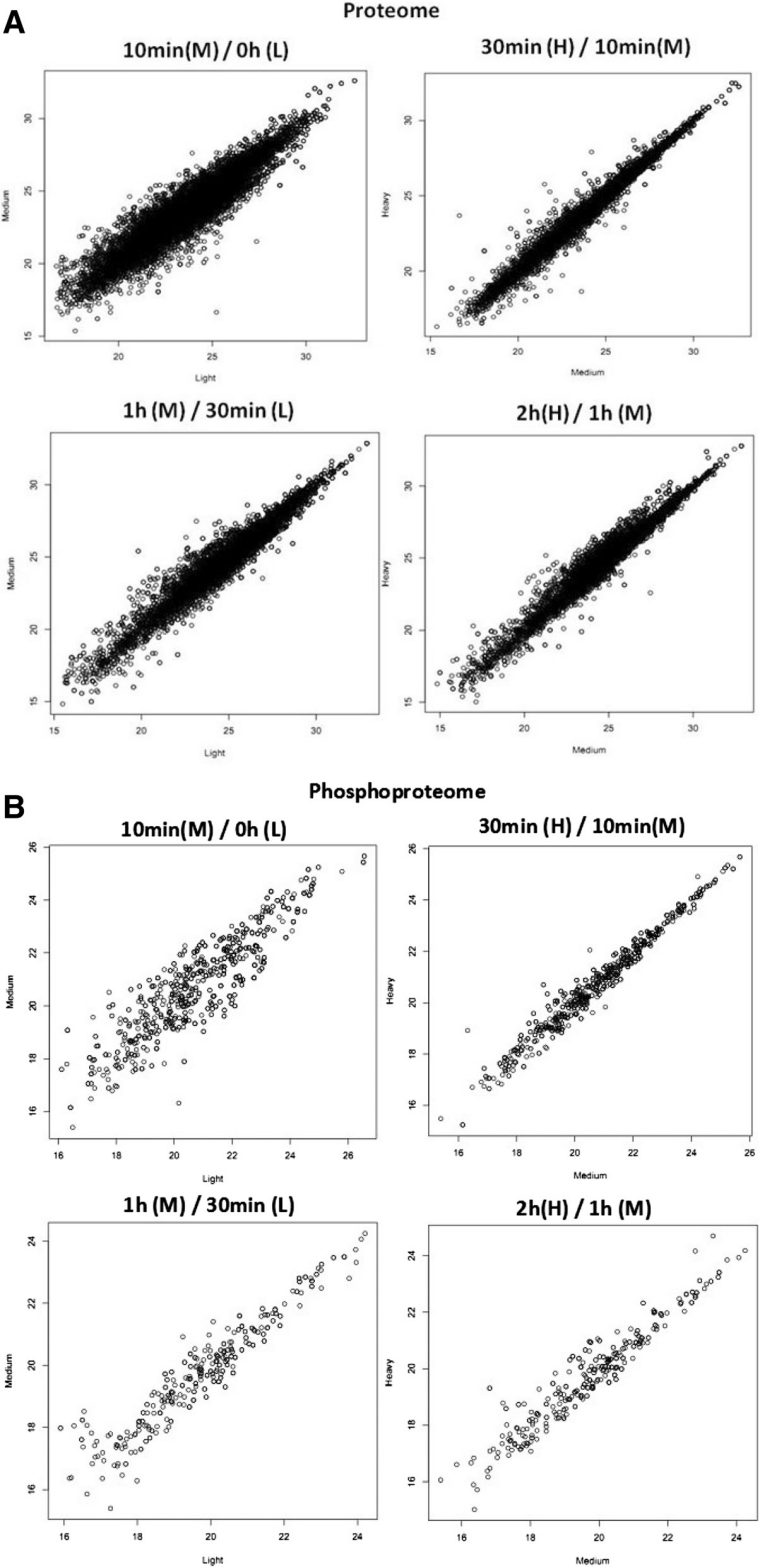
**Scatter plot analysis of labeled peptides.** The plot compares the different stable isotope labeled peptides (represented as circles) as follows: a) medium/light and b) heavy/medium. The 45º slope line shows a situation with no variation between the compared peptides, and circles that do not match in this line represent the peptides that were found to be differentially abundant. A) regular peptides; B) phosphorylated peptides.

The “raw” data acquired from Thermo LTQ-XL Orbitrap® was converted to “.mgf” files and an in-house MASCOT server was used to search for peptides containing dimethyl and carbamylation as a fixed modification and for phosphorylation in serine, tyrosine and threonine. The Thermo Proteome Discoverer software, version 1.1 was used to quantify all peptides based on the total area of Extracted Chromatogram (XIC), and the absolute values were normalized using a LOWESS (locally weighted scatterplot smoothing) algorithm (Figure 3). These data were input into the StatQuant software to evaluate the overall protein ratio by calculating the mean peptide ratio for all peptides corresponding to a given protein. The list for all peptides and phosphopeptides quantified can be accessed in the Additional file 1, and a summary of upregulated and downregulated phosphoproteins in each experiment, sorted by period of time indutction with rhBMP2 is shown in Additional file 2.

### Phosphosite localization

To assign phosphorylation sites, normalized Mascot delta score (nMD-score) was used [15]. Mascot delta score is the difference between the top two scores for the peptides identified by a given spectrum. Dividing this value by the score of the top score (rank 1) peptide, normalized delta score is obtained. In order to have 1% FLR (False Localization Rate) for correct phosphosite assignment with 99% certainty, peptides with nMD-score below 0.36 were discarded (corresponding to an MD-score of 10). A total of 950 unique phosphosites with 99% certainty that the sites were assigned correctly (1% FLR) were identified. These sites were found on 235 different proteins and their distributions were 87.5%, 11.5% and 0.8% for pS, pT and pY, respectively, which is comparable to previous works for mammalian cell types. All validated phosphosites with their MD-scores are listed in Additional file 1.

### Phosphorylation motif database search

**Figure 4:**
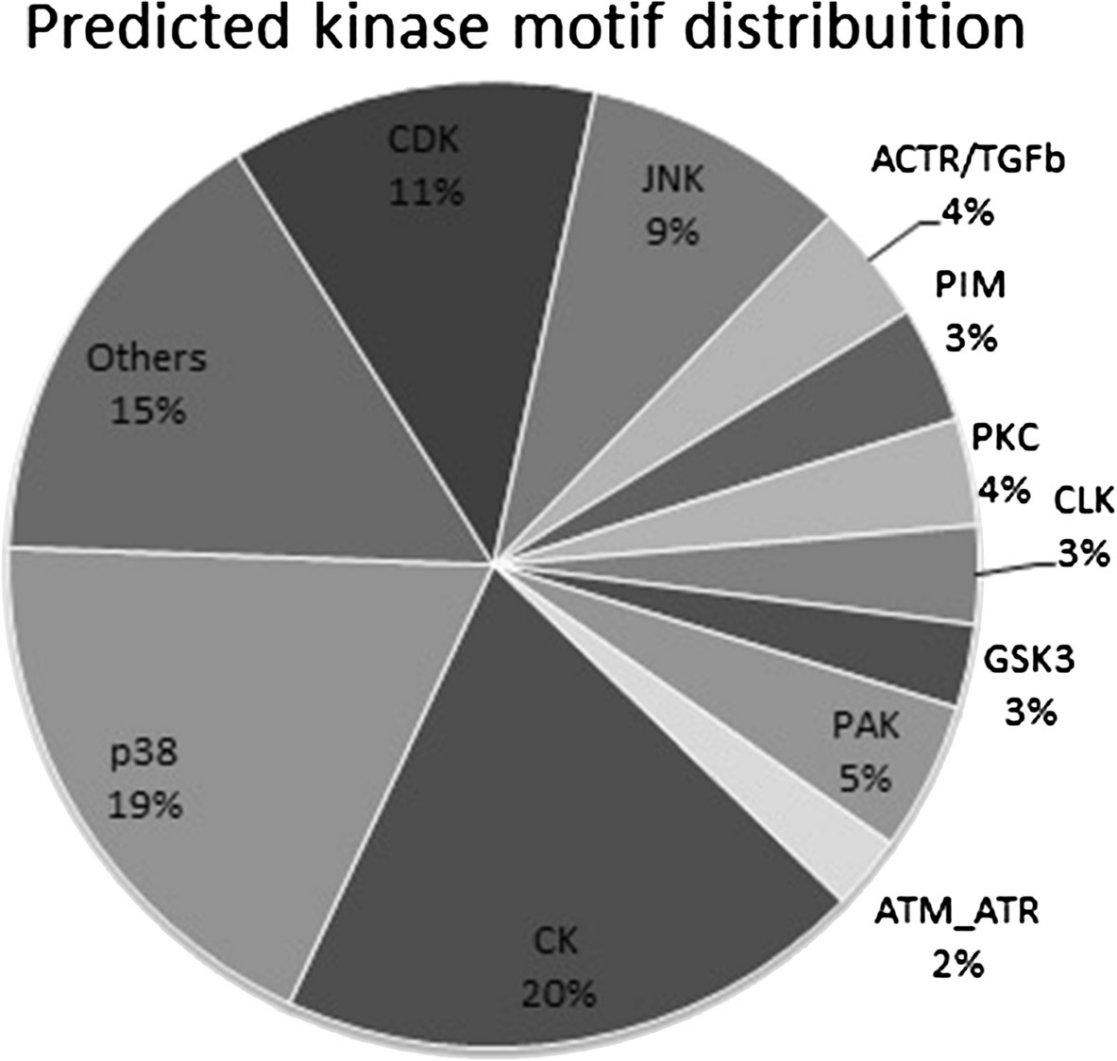
Predicted kinase motifs of substrates found in mass spectrometry experiments.

The analysis carried out to determine which kinase could possibly be involved in phosphsorylation of a given phosphorylated residue from phosphoproteome data was performed using the NetworKIN site (http://networkin.info/version_2_0/search.php). Figure 4 shows a summary of the complete dataset (Additional file 3) represented by a graph containing kinase motifs occurrencies.

### Network analysis using the ingenuity pathway analysis software

In order to evaluate possible intracellular interactors with the phosphopeptides found, a network analysis was performed. The Ingenuity Pathway Analysis software (http://www.ingenuity.com/) was used to map relationships among proteins, distributed into different cellular compartments. From the total list of proteins found to interact with phosphoproteins (Table 1 and Additional files 4 and 5), hits containing a transcription factor function were selected for further analysis of DNA-binding motifs in osteoblast differentiation-related genes. Non-phosphorylated population of peptides were classified according to biological process using the Gene Ontology Blast2Go software (http://www.blast2go.com) in order to map and annotate gene ontologies (Table 2).

**Table 1:** Transcription factors predicted to interact with phosphoproteins found in MS experiments

	
BARD1	BRCA1 associated RING domain 1
BMI1	BMI1 polycomb ring finger oncogene
CBX3	Chromobox homolog 3
CTNNB1	Catenin (cadherin-associated protein), beta 1, 88 kDa
HIC1	Hypermethylated in cancer 1
HMGA1	High mobility group AT-hook 1
HNF4A	Hepatocyte nuclear factor 4, alpha
ID3	Inhibitor of DNA binding 3, dominant negative helix-loop-helix protein
KDM5B	Lysine (K)-specific demethylase 5B
MAZ	MYC-associated zinc finger protein (purine-binding transcription factor)
MTA1	Metastasis associated 1
MYC	v-myc myelocytomatosis viral oncogene homolog (avian)
NFATC1	Nuclear factor of activated T-cells, cytoplasmic, calcineurin-dependent 1
NFKB1	Nuclear factor of kappa light polypeptide gene enhancer in B-cells 1
PPP1R13L	Protein phosphatase 1, regulatory (inhibitor) subunit 13 like
SIAH2	Seven in absentia homolog 2 (Drosophila)
SMAD3	SMAD family member 3
SOX4	SRY (sex determining region Y)-box 4
SP1	Sp1 transcription factor
SREBF1	Sterol regulatory element binding transcription factor 1
TFDP1	Transcription factor Dp-1
TGFB1I1	Transforming growth factor beta 1 induced transcript 1
TLE1	Transducin-like enhancer of split 1 (E(sp1) homolog, Drosophila)
YAP1	Yes-associated protein 1
CBX4	Chromobox homolog 4
E2F4	E2F transcription factor 4, p107/p130-binding

Predicted network of interaction for phosphoproteins found using Ingenuity Pathway Analysis. The list of phosphoproteins found were subjected to Ingenuity Pathway Analysis (IPA) to investigate problable protein interactions for each cellular compartment. Proteins described to be transcription factors were selected to investigate the activation of osteblast related genes by quantitative real-time PCR.

**Table 2:** Protein annotation for biological processes of upregulated proteins

**10 min/0 h**			**30 min/10 min**			**1 h/30 min**			**2 h/1 h**		
**GO term**	**#Seq**	**Score**	**GO term**	**#Seq**	**Score**	**GO term**	**#Seq**	**Score**	**GO term**	**#Seq**	**Score**
Signaling	**14**	**4,21**	Signaling	**31**	**8,92**	Signaling	**40**	**13,3**	Developmental process	**47**	**15**
Cellular response to stimulus	**19**	**5,67**	Anatomical structure development	**22**	**9,48**	Multicellular organismal development	**48**	**12,78**	Signaling	**17**	**4,13**
Anatomical structure morphogenesis	**13**	**4,21**	Multicellular organismal development	**8**	**2,39**	Anatomical structure development	**46**	**13,06**	Anatomical structure development	**41**	**12,3**
Cell differentiation	**18**	**5,95**	Cell differentiation	**6**	**2,48**	Cell-cell signaling	**9**	**2,96**	Multicellular organismal development	**43**	**8,57**
Regulation of signaling	**6**	**1,08**	Cell surface receptor linked signaling pathway	**3**	**2,6**	Cell activation	**7**	**3,18**	Cell differentiation	**20**	**9,36**
Cell morphogenesis involved in differentiation	**7**	**2,06**	Cell morphogenesis involved in differentiation	**30**	**5,77**	System development	**40**	**10,12**	Cell surface receptor linked signaling pathway	**9**	**3,63**
Organ development	**21**	**8**	G-protein coupled receptor protein signaling pathway	**4**	**3,2**	Cell differentiation	**33**	**15,09**	Embryo development	**7**	**3,14**
Regulation of cell differentiation	**6**	**1,12**	Signal transduction	**8**	**2,14**	Cytoskeleton organization	**26**	**9,55**	System development	**36**	**8,95**
Signal transduction	**11**	**5,73**	Epithelial cell differentiation	**13**	**6,01**	Regulation of signaling	**20**	**6,75**	Cell development	**15**	**5,97**
			Phosphorylation	**14**	**4,67**	Cell surface receptor linked signaling pathway	**18**	**7,95**	Cell morphogenesis involved in differentiation	**9**	**2,47**
			Transmembrane receptor protein tyrosine kinase signaling pathway	**4**	**2,56**	Signal transduction	**37**	**18,99**	Organ development	**26**	**10,7**
						Organ development	**26**	**11,08**	Regulation of signal transduction	**6**	**2,1**
						Cell morphogenesis involved in differentiation	**13**	**3,15**	Signal transduction	**17**	**6,54**
						Enzyme linked receptor protein signaling pathway	**9**	**3,8**	Epithelial cell differentiation	**2**	**2**
						Intracellular protein kinase cascade	**7**	**3,34**	Regulation of gene expression	**14**	**4,89**
						G-protein coupled receptor protein signaling pathway	**6**	**4,77**	Transmembrane receptor protein tyrosine kinase signaling pathway	**5**	**2,92**
						Small GTPase mediated signal transduction	**5**	**2,01**	DNA damage response, signal transduction by p53 class mediator resulting in cell cycle arrest	**3**	**3**
						Second-messenger-mediated signaling	**3**	**1,22**	Negative regulation of ubiquitin-protein ligase activity involved in mitotic cell cycle	**4**	**4**
						Wnt receptor signaling pathway	**3**	**1,08**	Positive regulation of ubiquitin-protein ligase activity involved in mitotic cell cycle	**4**	**4**
						Phosphorylation	**17**	**8,1**	Regulation of ubiquitin-protein ligase activity involved in mitotic cell cycle	**4**	**4,8**
						Positive regulation of signal transduction	**9**	**5,2**			
						Transmembrane receptor protein tyrosine kinase signaling pathway	**9**	**6,33**			
						MAPKKK cascade	**5**	**4**			
						Negative regulation of cell proliferation	**5**	**4,6**			
						Positive regulation of intracellular protein kinase cascade	**5**	**3**			
						Negative regulation of cell cycle	**4**	**1,84**			
						Post-translational protein modification	**4**	**4**			
						Ras protein signal transduction	**4**	**1,68**			
						Regulation of Wnt receptor signaling pathway	**3**	**1,56**			

Consistently upregulated proteins were input in Blast2go software for mapping and annotation of gene ontologies. Biological processes over-represented were selected and classified according to level. Top: low levels; bottom.

### Analysis of the promoter region of osteoblast-related genes for the presence of responsive elements for the BMP2-regulated transcription factors

**Figure 5:**
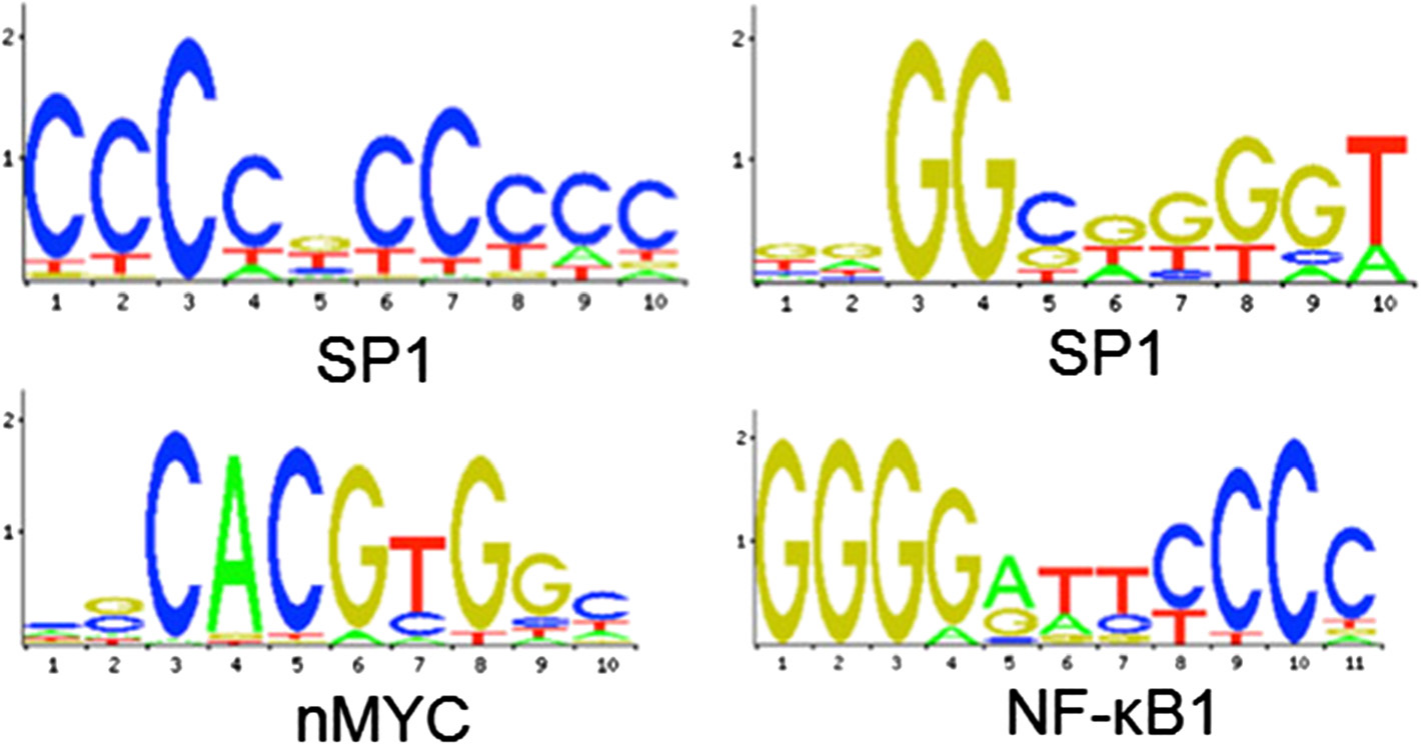
Transcription factor motifs found for selected osteodifferentiation genes.

After obtaining the list of transcription factors for the Ingenuity® network analysis, a curated database for transcription target genes, TRED was used to find target genes and text mining was performed to find which target genes are related with osteoblastic differentiation. We used the JASPAR database [16] which contains a curated, non-redundant set of profiles, derived from published collections of experimentally defined transcription factor binding sites for eukaryotes and sorted out the transcription factor which have well defined binding motifs (Figure 5) [17-19]. These motifs were used as a template for a search in the promoter region of the pre-selected genes, using the ENSEMBL cisRED database [20] and those which displayed at least one match or multiple matches for the sequences were selected for the qRT-PCR analysis (Additional file 6). The consensus sequences of sp1, c-Myc and NFkB were selected among others because they were present in the promoter region in more them 80% of the selected genes for qPCR validation.

### Analysis of differentially expressed genes involved in osteoblastogenesis activated by BMP2-induced transcription factors

**Figure 6:**
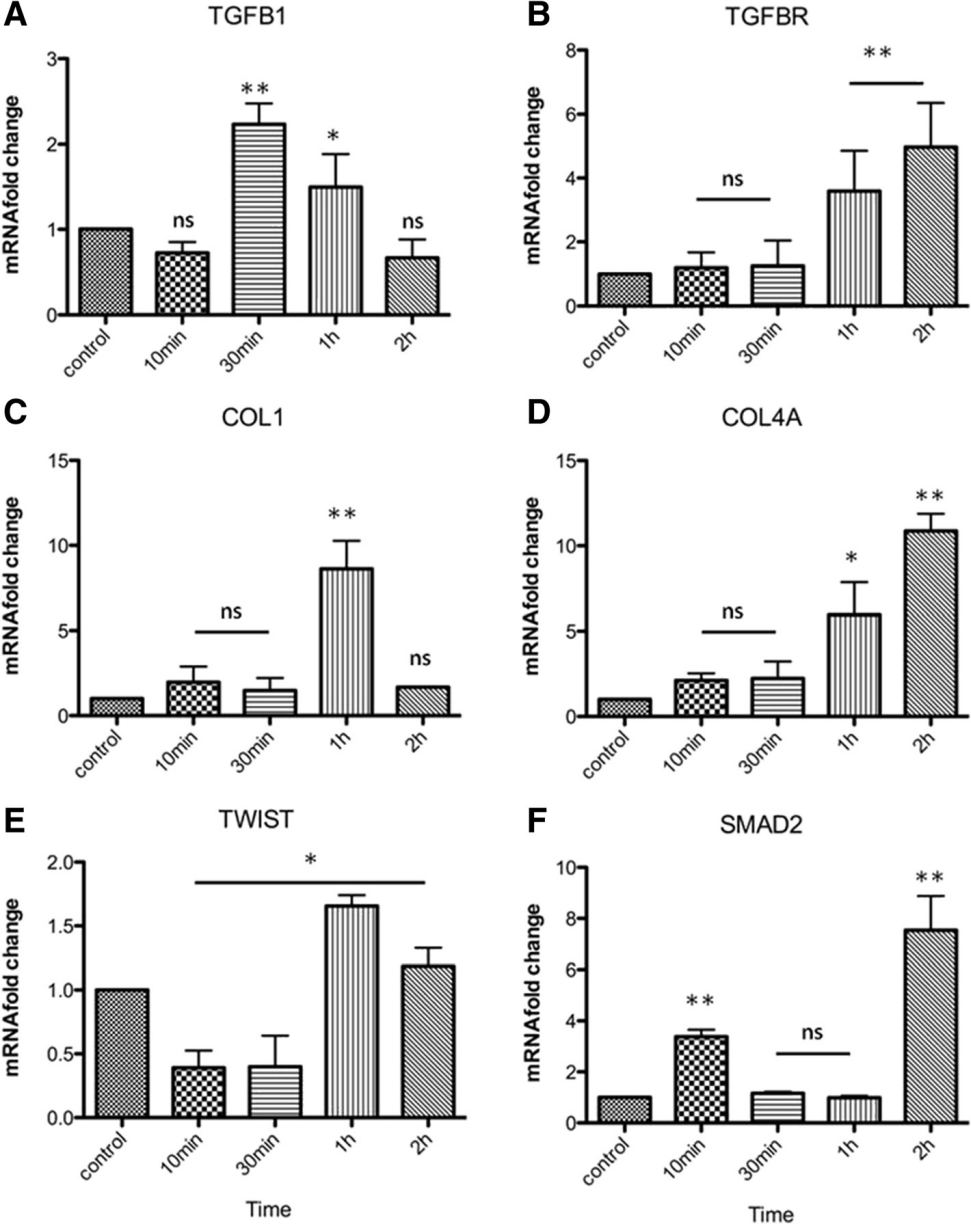
**mRNA fold change measured by qRT-PCR for osteoblast related genes.** Quantitative Real Time PCR of osteoblast related genes from rhBMP2 induced sMSC at indicated timepoints were normalized and compared according to housekeeping genes as control: mHPRT - hypoxanthine phosphoribosyltransferase 1, mGAPDH - glyceraldehyde-3-phosphate synthase and mHMBS - mouse hydroxymethylbilane synthase. **A)** TGFB1 relative levels were upregulated 3.2 fold after 30 min of rhBMP2 induction (p<0.001) followed by a decrease to basal levels after 2 h. **B)** TGFBR1 mRNA increase levels of up to 3.6 fold at 1 h and more than 4.9 fold at 2 h (Figure 6B, p<0.001). **C)** COL1 displays a punctual increase at 1 h after stimulus (8.6 fold, p<0.001). **D)** COL4A showed a progressively rising pattern at timepoints studied (5.96 fold at 1 h and 10.8 at 2 h, p<0.05 and p<0.001, respectively). **E)** TWIST is downregulated (0.39 fold after 10 min) but after 1h shows a slight increase (1.6 fold) at 1 h. **F)** SMAD2 presented a slight increase of 3.4 fold at 10 min and 2 h (p<0.001). Data represent three independent experiments and standard deviation bar is shown for all experimental timepoints studied. Asterisks represents levels of significance for statistical analysis (Two-Way ANOVA, Bonferroni test) between control and treatment groups (*p<0,05 and **p<0,01). ns: non statistically significant.

We used analysis of regulatory networks in order to investigate which transcription factors were activated, and which of them are related with activation of osteoblast-related genes (Table 3). Thirteen genes were selected to evaluate their role in osteoblastic differentiation of msMSC cells, and to confirm the *in silico* analysis. From the initial list of genes investigated, ten (TGF-beta receptor, TGF-beta induced protein, Collagens 1and 4a1, Sox9, Smad2,Twist and the osteoblast-specific genes RUNX2, OSX and DLX5) were found to be upregulated at different timepoints (Figure 6). The TGFB cytokine ant its receptor, TGFBR1, displayed the regulated motifs in their promoter regions. The mRNA relative levels of these two genes were evaluated after 10 min, 30 min, 1 h and 2 h of exposure to rhBMP2. The relative levels of TGFB1 were upregulated more than two times after 30 min of rhBMP2 induction (Figure 6A, 3.2 fold, p<0.001), but after reaching this peak, the relative levels decreased to basal levels after 2 h. This pattern was followed by a subsequent increase in the TGFBR1 mRNA relative levels of up to 3.6 fold at 1 h and more than 4.9 fold at 2 h (Figure 6B, p<0.001). Since the synthesis of extracellular matrix compounds, such as collagens, is known to be regulated during osteo differentiation, we selected two members of the collagen family that displayed the selected motifs, namely: collagen 1 and 4a. Both ECM components were upregulated, with collagen 1 displaying a punctual increase at 1 h after stimulus (Figure 6C, 8.6 fold, p<0.001) and collagen 4a followed a progressively rising pattern (Figure 6D, 5.96 fold at 1 h and 10.8 at 2 h, p<0.05 and p<0.001, respectively). Related to collagens and TGFB, the osteogenesis related gene Twist presents a downregulation pattern (0.39 fold after 10 min) from the basal levels during the beginning of the differentiation and after that a slight increase (1.6 fold) at 1 h, a decrease to 1.2 fold at 2 h (Figure 6E, p<0.05). Expression of SMAD2 was accessed, in order to stabilish a possible crosstalk between TGF-beta/Activin signaling during BMP2-driven osteogenesis. The mRNA relative levels of SMAD2 were accessed, presenting a slight increase of 3.4 fold at 10 min and a major increase of more than 7.5 fold at 2 h (Figure 6F, p<0.001).

**Table 3:** List of Primers used in qRT-PCR

**Gene**	**5′- 3′ nucleotide sequence**
**mBMP-2 F**	TCTTCCGGGAACAGATACAGG
**mBMP-2 R**	TGGTGTCCAATAGTCTGGTCA
**mOsterix F**	ATGGCGTCCTCTCTGCTTG
**mOsterix R**	TGAAAGGTCAGCGTATGGCTT
**mRunX-2 F**	GACTGTGGTTACCGTCATGGC
**mRunX-2 R**	ACTTGGTTTTTCATAACAGCGGA
**mDlx5 F**	CACCACCCGTCTCAGGAATC
**mDlx5 R**	GCTTTGCCATAAGAAGCAGAGG
**mTGFb1 F**	CTCCCGTGGCTTCTAGTGC
**mTGFb1 R**	GCCTTAGTTTGGACAGGATCTG
**mSMAD-2 F**	ATGTCGTCCATCTTGCCATTC
**mSMAD-2 R**	AACCGTCCTGTTTTCTTTAGCTT
**mTGFbR2 F**	CCGCTGCATATCGTCCTGTG
**mTGFbR2 R**	AGTGGATGGATGGTCCTATTACA
**mTwist1 F**	GGACAAGCTGAGCAAGATTCA
**mTwist1 R**	CGGAGAAGGCGTAGCTGAG
**mSox9 F**	GAGCCGGATCTGAAGAGGGA
**mSox9 R**	GCTTGACGTGTGGCTTGTTC
**mCol4a1 F**	CTGGCACAAAAGGGACGAG
**mCol4a1 R**	ACGTGGCCGAGAATTTCACC
**mCol-1 F**	GCTCCTCTTAGGGGCCACT
**mCol-1 R**	ATTGGGGACCCTTAGGCCAT
**mCol-2 F**	CAGGATGCCCGAAAATTAGGG
**mCol-2 R**	ACCACGATCACCTCTGGGT

List of primers used to amplify the cDNA in order to investigate changes in mRNA upon BMP2 treatment in qRT-PCR. F: forward; R: reverse.

**Figure 7:**
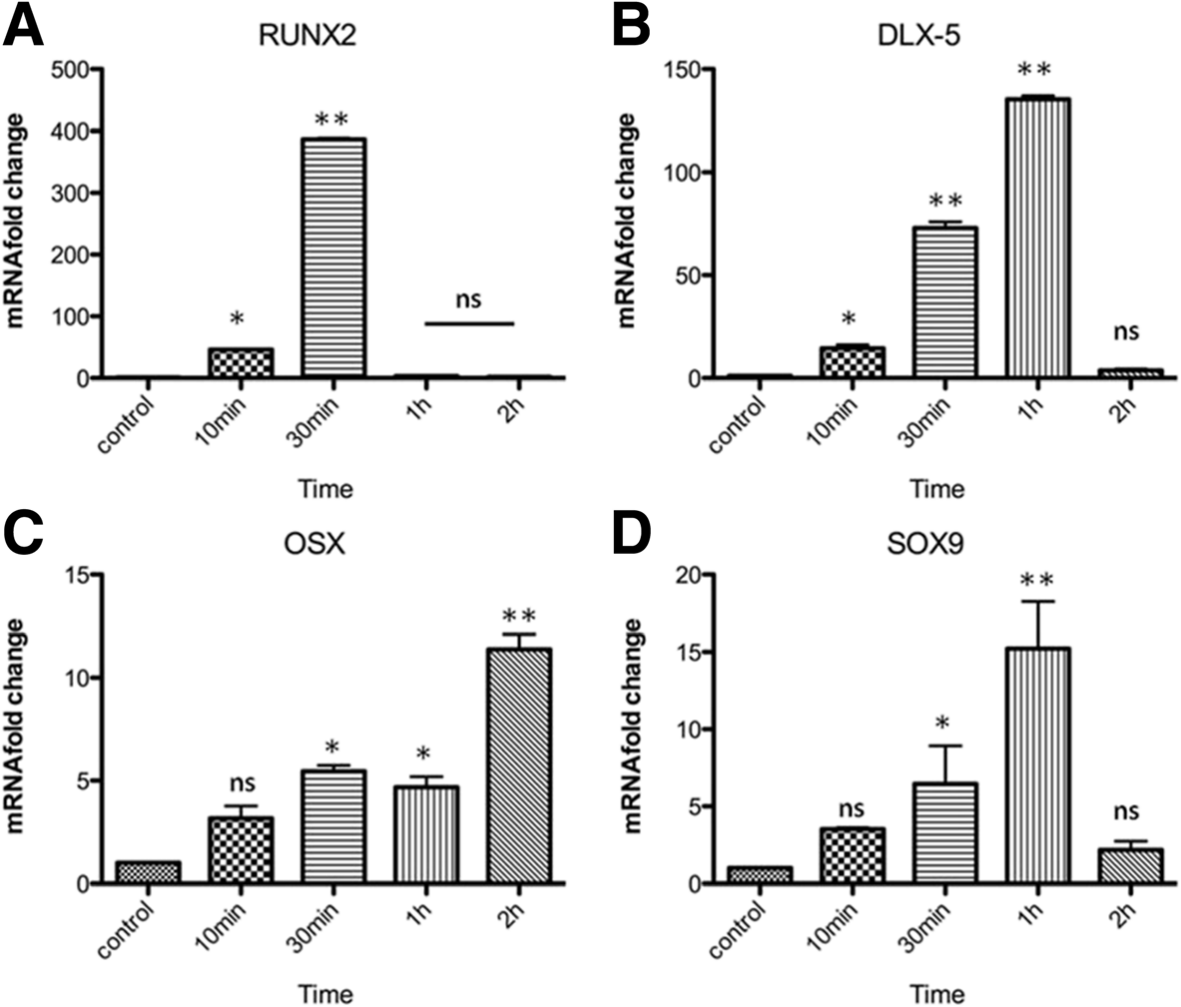
**mRNA fold change measured by qRT-PCR for osteoblast related genes (continued). A)** RUNX2 showed an intense upregulation, increasing almost 400 fold after 30 min, with a drastic descent to levels similar to basal levels after 1 h (p<0.001). **B)** DLX-5, displayed an increase at 10 min and at 30 min (14.4 fold, p<0.05, 72.9 fold, p<0.001, respectively) reaching a peak at 1 h (135.2 fold, p<0.001), followed by a decrease to basal levels at 2 h. **C)** OSX showed an increase, from 10 min to 2 h after stimulation (p<0.001). **D)** SOX9 was upregulated at 30 min (6.4 fold, p<0.05) and 1 h (15.2 fold, p<0.001). Data represent three independent experiments and standard deviation bar is shown for all experimental timepoints studied. Asterisks represents levels of significance for statistical analysis (Two-Way ANOVA, Bonferroni test) between control and treatment groups (*p<0,05 and **p<0,01). ns: non statistically significant.

We also evaluated a set of four transcription factors which, in addition to presenting the regulated motifs in their promoter regions, were key elements during the osteoprogenitors differentiation. The relative mRNA levels of RUNX2 were the first to be upregulated, increasing almost 400 fold after 30 min, with a drastic descent to levels similar to basal levels after 1 h (Figure 7A, p<0.001). Another important transcription factor, DLX-5, displayed a progressive increase at 10 min (14.4 fold, p<0.05) and 30 min (72.9 fold, p<0.001) reaching a peak at 1 h (135.2 fold, p<0.001), followed by a sharp decrease to basal levels at 2 h (Figure 7B). The transcription factor Osterix displayed a stepwise increase, beginning at 10 min, and reaching up to 10 fold after 2 h of stimulation (Figure 7C, p<0.001). Similarly, the SOX9 mRNA level was upregulated at 30 min (6.4 fold, p<0.05) and 1 h (15.2 fold, p<0.001) (Figure 7D).

## Discussion

In the present study, we used murine skin mesenchymal cells (msMSC) and stable dimethyl isotope labeling to quantify abundant proteins and phosphoproteins using TiO_2_ metal affinity chromatography, coupled with mass spectrometry, at five different periods of rhBMP2 induction, namely: 0, 10, 30, 60 and 120 min. From 150 μg of the combined samples, it was possible to identify and quantify 235 distinct phophoproteins and 2,029 distinct proteins, in all replicates. Based on the data acquired, and, also, on references from the literature, we proposed a model for BMP2-mediated osteodifferentiation differentiation of these msMSCs cells. Previous experiments carried out with these msMSCs, subjected to the osteoblast differentiation medium (ascorbic acid, beta-glycerolphosphate and dexamethasone) showed intense calcification at 14 and 21 days of treatment, with greater than 80% of the cells being Alizarin Red-positive” (Forni et al., unpublished data). This experiment could not be carried out solely with BMPs-supplemented culture medium, due to its lack of mineral components (e.g. beta-glycerolphosphate), which is necessary for mineralization.

**Figure 8:**
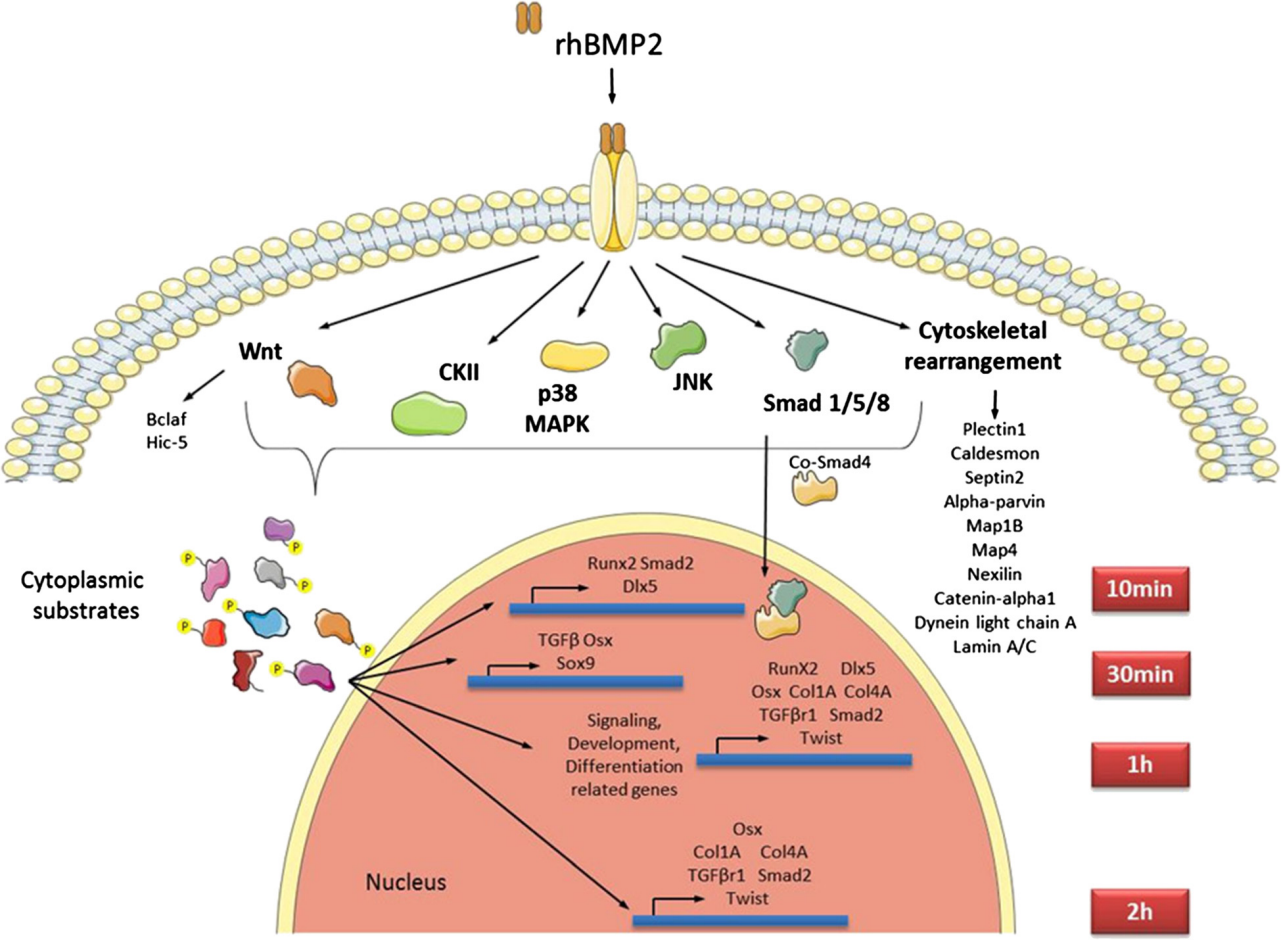
Proposed model for BMP2-mediated osteoblast differentiation in msMSCs.

The data found showed to be compatible with bone development, since BMPs act at the very early stages of cells differentiation to the osteoblastic lineage, but, later on in the process, these cells incorporate mineral precursors and originate the calcified bone tissue. The kinases which showed the highest number of phosphorylation motifs in phosphodata were represented, as well as gene activation for each time period studied (Figure 8).

We used triplex stable isotope dimethyl labeling to compare five different time periods of rhBMP2-induced osteoblastic differentiation of skin mesenchymal cells, combined into two different experimental groups (an “early period” group, which comprised the 0 h, 10 min and 30 min samples and a second, “late period” group, comprised of the 30 min, 1 h and 2 h samples). This was necessary in order to correctly compare the phosphoprotein ratios with their respective protein levels, since we do not expect a wide protein level variation during the period studied and, also, to avoid aberrations in phosphoprotein variation. Similarly, Song and colleagues [21] used similar approach through a pseudo-triplex dimethyl labeling in liver tissue samples and hepatocellular carcinoma.

Each pooled sample was split according to the affinity of peptides for the TiO_2_ column. The TiO_2_-Flowthrough fraction was subjected to HILIC and fractionated. Since this step decreased the sample complexity, it enhanced the number of peptides which could be identified and quantified, when compared to the entire pool, which was directly injected into the LC/MS (data not shown). Since we started from a relatively low amount of sample (50 μg of protein extract for each timepoint and 150 μg for a triplex experiment), no improvement in the number of detected peptides was obtained using HILIC in phospho-enriched samples. Analysis of the data also showed that there is a tendency for many phosphopeptides to be upregulated between 30 min and 1 h of rhBMP2 treatment, correlating with the period of activation for the Dlx5 transcription factors which trigger the expression of RUNX2 and OSX, both of which are upregulated upon rhBMP2 administration [22,23].

In order to compare measurements across LC-MS/MS experiments and to correct for non-biological variation (such as the bias generated in the sample quantification), data normalization is a crucial step prior to any further analysis. The standard normalization assumed in LC/MS experiments is based on dividing all peptide ratio values by log2. However, notice that this procedure only divides the peptide abundance by a common factor, re-scaling the relative abundance of the peptide. In other words, this “within sample” normalization does not remove the bias in the quantities across experiments. In order to remove the systematic errors introduced in different experiments, we applied the LOWESS regression, a technique commonly applied to microarray data analysis [24]. One premise to apply LOWESS normalization is that the differences among the overall intensity of different experiments would be the consequence of non-biological variation, i.e., most peptides will not show a significant change in the abundance between the two compared samples (peptides derived from closely related samples). Briefly, in a well performed experiment, the scatter-plot of peptides of one sample versus another would cluster the peptides along a straight line, and the slope would be equal to 1. Normalization of these data is equivalent to calculating the best-fit slope using regression techniques and adjusting the intensities so that the calculated slope is 1. However, sometimes, the intensities may be non-linear, therefore, local regression techniques, such as LOWESS regression, are more suitable. LOWESS regression [25] is estimated through a locally weighted polynomial regression for a subset of peptides in the neighborhood of each peptide. For more details, please refer to [26].

### BMP2 induces phosphorylation of substrates for different kinases in msMSCs

Kinase prediction analysis using the NetworKIN database, from the phosphorylated peptides found, suggested that, three major kinases could be acting as effectors of phosphorylation upon BMP2 treatment, namely: Casein kinase II (CK2), p38 MAPK and JNK. These kinases are followed, to a lesser extent, by Activin receptors and the CDK family of kinases. These data are in agreement with the literature of the field, since Bragdon and colleagues [27,28] showed the involvement of CK2 in BMP2-induced cells. The release of CK2 from BMP receptor type I (BMPRI) is related with osteblastogenesis, since specific peptides which interfere with this interaction, destabilize the CK2-BMPRI complex and enhance osteoblastic differentiation. It is possible that the role of CK2 in osteogenesis is much more than its release from BMPRI, involving many of the substrates found in this work and even other ones which could contribute to the enrollment of these undifferentiated stem cells to osteoblastogenesis.

The involvement of p38 MAPK in BMP2-driven osteoblatogenesis is well-known. Several studies [1,23] show activation of p38 within the first hour of BMP2 induction, and activation of Dlx5 and Osx, essential genes involved in osteblastic differentiation [23], as well as alkaline phosphatase. We confirmed these data in our model using quantitative real-time PCR experiments, showing an increase in mRNA relative expression for Osx and Dlx5. It is interesting to note that p38 may be involved in phosphorylation of several phosphoproteins found in our study, since 120 sites were predicted to be phosphorylated by this kinase.

Upon BMP2 treatment, JNK may also be activated, as previous studies described [29-32]. We found that 9% of all sites could be phosphorylated by this kinase up to 2 h of BMP2 treatment. Interestingly, JNK is transiently activated in MC3T3-E1cells, in a short window (from 2 h to 4 h after BMP2 induction), stimulating the expression of osteocalcin. However, at late periods of BMP2 induction, JNK acts inhibiting the RUNX2 function by its phosphorylation at Ser 104 in C2C12 cells [30]. These results show the dual function of JNK in osteoblastogenesis, which is regulated in a time-dependent manner. At early periods of time, JNK may have a role inducing osteogenesis, by phosphorylating intracellular substrates and augmenting the cellular sensibility for BMP2 [31]. On the other hand, at late periods, JNK would participate by slowing down the intracellular signaling for osteodiffentiation.

Similar number of phosphorylated sites were found for the CDK group of kinases. These kinases are related with cell cycle progression, and their activation or inhibition is associated with proliferation and quiescence, respectively. At a first glance, the activity of CDK kinases could lead to an impairment of osteoblastic differentiation, due to stimulation of cell proliferation. The role of CDK in osteoblastic differentiation is not well understood yet, however, its inhibitor, the p21 protein, has been involved in osteoblastic differentiation since p21 null mice exhibit enhanced osteoblastic differentiation, and overexpression of p21 protein delays bone formation [33]. It is possible that p21 could act independently of CDK, activating or repressing genes in the nucleus, with its role controlling osteoblastic differentiation being more complex than simply regulating the cell cycle. Further studies will be required to address this issue and how CDK/p21 regulation participates in osteoblastic differentiation.

### Biological processes overrepresented in BMP2-treated msMSCs

The proteomic data obtained were analyzed using the Gene Ontology classification. We observed which gene ontologies could be representative of the upregulated genes. Surprisingly, we found a high number of ontologies containing the following terms: multicellular organismal and anatomical structure development (48 and 46 entries, score 12.78, and 13.06 respectively), signal transduction (37 entries, score 18.99) signaling (40 entries, score 13.3), cell differentiation (33 entries, score 13.06), cell surface receptor linked signaling pathway (18 entries, score 7.95) and phosphorylation (17 entries, score 8.1) at the first hour of BMP2 treatment, in contrast with the first 10 and 30 min periods of induction, which showed a few gene ontologies with these terms assigned. This can be due to the fact that short periods of time are not sufficient to change the overall amount of protein in the cell [34], therefore, transcription and translation of new proteins must take place before we can observe changes in protein levels, which are sufficient to affect the gene ontologies classification observed. Nevertheless, comparing the second hour of BMP2 induction with the first one, less gene ontologies could be classified, leading to the conclusion that these proteins involved with signaling are regulated within the first hour BMP2 induction.

### BMP2-treated msMSCs phosphorylate intracellular messengers which, in turn, activate osteoblastic-related genes

BMP2 induction was shown to modify the post-translational modifications of intracelular proteins, at the timepoints studied. In order to investigate how these phosphorylated proteins activate transcription factors, and whether they are related with the activation of osteoblastic genes, a network analysis of proteins found in the phosphoproteome of BMP2-treated msMSCs was carried out. Through Ingenuity network analysis, we found different transcription factors related with the phospho data. However, not all of the transcription factors found were described to have any participation in osteoblast differentiation, or activation of osteoblastic related genes. Using a curated database for transcription target genes, TRED, a transcription factors binding motifs occurrence, JASPAR, and the literature on the field to search for osteoblastic target genes, one by one, we found three transcription factors from the Ingenuity output list, displaying important roles in osteoblastogenesis, namely: SP1, c-Myc e NF-ƙB.

TGF-β/BMPs are widely recognized for their role in bone formation during mammalian development, exhibiting versatile regulatory functions in the body [35]. In accordance with this finding, we observed increased levels of the mRNA for both the TGFB cytokine and for its receptor TGFBR. Also, signaling transduction by TGF-β/BMPs occurs specifically through both canonical Smad-dependent pathways (TGF-β/BMP ligands, receptors and Smads) and a non-canonical Smad-independent signaling pathway (e.g. p38 mitogen-activated protein kinase pathway, MAPK). Following TGF-β/BMP induction, both the Smad and p38 MAPK pathways converge at the RUNX2 gene to control mesenchymal precursor cell differentiation, which has also been found to have increased mRNA levels.

SOX9, a transcription factor of the sex-determining region Y (SRY)-related high mobility group box family of proteins, is crucial for skeletal development and marks all osteoblastic progenitors [36], being capable of inducing RUNX2 expression [37-39] . However, the role of SOX9 in osteoblastic differentiation is not completely understood. Conditional deletion of SOX9 in the limb bud mesenchyme led to the absence of chondrocytes and osteoblasts [40]. Contrastingly, when SOX9 was deleted in the neural crest cells that contribute to the craniofacial skeleton, the cells which normally form chondrocytes expressed osteoblasts markers [41], suggesting the existence of a the bipotential progenitor. However, SOX9 is not expressed by mature osteoblasts [36] and this is the probable cause of its downregulation after 2 h of the stimulus.

COL1 and COL4A display functions related with the building of the basal membrane for the newly-formed mature bone tissue. A recent report of comparative transcription of various fetal and adult mesenchymal stem cells sources through quantitative PCR profiling unveiled that collagens, such as collagen 1 and 4, were upregulated during several types of osteogenic differentiation, such as the one reported in this manuscript with the levels of these two extracellular matrix components being increased [42]. Supporting these findings, it has been reported that site mutations in collagen 1 leads to high bone mass in osteogenesis imperfecta [43].

Since the bHLH transcription factor Twist inhibits osteoblast differentiation through binding to a DNA-binding domain in RUNX2 [44], the early downregulation of this gene to levels below the basal level at 10 and 30 min could be indicative that the differentiation process was mediated by RUNX2. Moreover, it has been shown that RUNX2, a Runt domain-containing transcription factor, is indispensable for osteoblastic differentiation during both endochondral and intramembranous ossification and the function of mature osteoblasts, including the synthesis of bone matrix. Homozygous deletion of Runx2 in mice resulted in a complete lack of osteoblasts [41]. Our results show a sustained increase in the mRNA levels of this transcription factor after 30 min, pointining to the involvement of this gene in the osteogenesis induced by exposure to BMP2. Another essential gene related with osteoblastic differentiation is OSX, a transcription factor containing three zinc-fingers [45]. OSX was discovered as a BMP-induced gene in C2C12 cells, with its deletion resulting in complete absence of osteoblasts in mouse embryos, despite the relatively normal expression of RUNX2 [39], which indicates that OSX is activated after RUNX2 during osteoblastic differentiation. In accordance, we observed that after a significant increase in RUNX2 after 30 min of induction, a consistent increase of mRNA OSX levels is observed up to 2 h after stimulus. Finally, we analized the expression levels of DLX-5, an homeobox gene that plays an essential role in craniofacial, axial, and appendicular skeletal development [46], and specifically regulates RUNX2 expression by binding to the homeodomain-response elements in the RUNX2 distal promoter [47]. The increased amounts of DLX-5 after exposure to BMP2 indicates that this gene is also present in our differentiation event, generating a reliable axis between DLX-5/RUNX-2/OSX.

### Novel phosphorylated candidates found upon BMP2 treatment of msMSCs

From all three independent experiments, we chose proteins which displayed increased phosphorylation upon BMP2 treatment: a group of proteins related with cytoskeletal rearrangement and Ras protein signal transduction. Cytoskeletal rearrangement is observed during osteoblastic differentiation through the shift from a fibroblast-like to a spheric phenotype, upon induction with supplemented osteogenic differentiation medium (ODM), being antagonized by treatment with cytochalasin D, leading to a reduction of differentiation markers expression [48]. Thus, catenin alpha 1, alpha parvin, septin-2, caldesmon, microtubule associated proteins 1B and 4, nexilin, cytoplasmic dynein 1 light intermediate chain and isoforms of lamin A/C and plectin 1 were found to be upregulated at all time periods studied. Together with the previous studies which had described activation of these proteins using ODM [48,49], we found that these proteins were also activated upon BMP2 treatment. This may be explained by the fact that a common subset of proteins can be activated by both BMP2 and components of ODM, phosphorylating other proteins related which cytoskeletal rearrangement. Another protein related with cytoskeletal rearrangement found in our experiments was Rho GTPase-activating protein. The Rho family of GTPases plays an important role in osteoblastic differentiation, shown by differentiation to osteogenesis of constitutively RhoA-expressing mesenchymal stem cells [50]. Other proteins involving signaling pathways in osteoblastic differentiation were positively phosphorylated, namely: Transforming growth factor beta 1 induced transcript (Hic-5) and Bcl-2-associated transcription factor 1 displayed increasing phosphorylation levels. These proteins are related to the Wnt pathway and, specifically, Hic-5 was involved in regulation of intracellular signals by Smad 1, 5 and 8, effector proteins of the canonical BMP2 signaling pathway [51].

## Conclusions

Stable isotope dimethyl labeling of peptides may be used to quantify small amounts of proteins phosphorylated in cell extracts. During BMP2-induced differentiation in skin derived mesenchymal stem cells, it was possible to acess different proteins, which many of them were found to be phosphorylated in different timepoints, giving new cues about the events that occur in the short term of osteoblastic differentiation.

## Methods

### Cell isolation

The cells were isolated from BALB/C mice dermis through careful dissecation from skin and FACS sorting, being CD105+, CD73+, CD90+, lacking CD14 and CD34 as surface markers (more than 90% purity), being able to growth under plastic and differentiate into osteoblastic cells by osteodifferentiation-induced assay (ascorbic acid, beta-glycerolphosphate and dexamethasone) and Alizarin-Red stainig after 14 and 21 days. These cells were also capable of chondro, osteo and adipogenesis, validated through histochemistry and gene expression assays (Forni et al., in preparation), as described in the literature [52].

### Materials

The protease (Benzonase®) and phosphatase inhibitor cocktail (Complete®), were purchased from Roche (Mannheim, Germany). Modified porcine trypsin was purchased from Promega (Fitchburg, USA). DTT, ammonium bicarbonate, sodium cyanoborohydride, iodoacetamide, triethylammonium bicarbonate (TEAB) and glycolic acid, were from Sigma (Steinheim, Germany). CD_2_O (98% D, 20 wt%),^13^CD_2_O (99% ^13^C, 98% D, 20 wt%), and sodium cyanoborodeuteride (96% D) were from Isotec (Miamisburg, USA). Formaldehyde (37%) and ammonia solution (25%) was purchased from Merck (Darmstadt, Germany). Poros Oligo R3 reversed-phase material was from PerSeptive Biosystems (Framingham, USA). TiO2 beads were obtained from GL Science (Tokyo, Japan). EmporeTM C8 extraction disk was from 3 M Bioanalytical Technologies (St Paul, USA). The water used in all experiments was obtained from a Milli-Q purification system (Millipore, USA). All other chemicals were purchased from commercial sources and were of analysis grade.

### Total protein extract from murine-derived mesenchymal stem cells induced with rhBMP2

Cell extracts from mesenchymal stem cells were made as previously described [53], with some modifications. Briefly, murine skin-derived mesenchymal stem cells (msMSC) obtained in our laboratory, were seeded onto 100 mm diameter culture plate in Dulbecco’s modified Eagle’s Medium (DMEM) containing Glutamax-I (Life Technologies, USA), 1% penicillin/streptomycin (Life Technologies, USA) and 10% fetal bovine serum (Life Technologies, USA) at 37°C until they reached 90% confluence. The medium was then changed in each experimental group (10 min, 30 min, 1 h and 2 h) for DMEM supplemented with 200 ng/ml of rhBMP2 (Life Technologies, USA) and 10% fetal bovine serum. After the induction period, the cultures were washed twice with ice-cold PBS buffer. After washing, cells were harvested and the cell suspension was then centrifuged at 1,000 g for 5 min. The cell pellet was ressuspended in 100 μl of lysis buffer (7 M urea (Sigma, Steinheim, Germany), 2 M thiourea (Merck), 1% N-octyl glycoside (Sigma, Steinheim, Germany), 40 mM Tris (Sigma, Steinheim, Germany) containing phosphatase and proteinase inhibitors (PhosStop and Complete – Roche) and 300 units of Benzonase (Merck). The cells were then sonicated at 40% output with intervals of 3 × 15 s on ice to disrupt the cells and then incubated at −80°C for 30 min. After incubation, 20 mM DTT was added, and samples were incubated at room temperature for 35 min. Iodoacetamide (40 mM) was then added, followed by incubation for 35 min at room temperature in the dark. For protein precipitation, 14 ml of ice-cold acetone was added to the solution, followed by incubation at −20°C for 20 min. The proteins were pelleted by centrifugation at 6,000 g for 10 min at 4°C, and the pellet was stored at −20°C until further use. The BCA method was used to determine the protein concentration of each sample.

### Tryptic digestion of total protein extracts

Precipitated proteins from msMSC cells were solubilized in 100 mM TEAB, and 50 μg of total protein extract, quantified by the bicinchoninic acid assay kit (Pierce, Waltham, USA), incubated with chemically modified trypsin at a proportion of 1:100, and subsequently incubated at room temperature for 18 h.

### R3 microcolumns for desalting

The Poros Oligo R3 reversed phase resin was suspended in 70% acetonitrile. The R3 beads were loaded onto constricted GELoader (Eppendorf, Hamburg, Germany) tips containing a C_8_ microdisc and gentle air pressure was applied to pack the beads in order to obtain R3 microcolumns of 3 mm [54]. Each acidified sample was loaded onto an R3 microcolumn. The R3 microcolumns were subsequently washed with 30 μl of 0.1% TFA, and the peptides were eluted from the Poros R3 column using 30 μl of 70% acetonitrile, 0.1% TFA. The phosphopeptides were subsequently ressuspended in 0.5 μl of 100% formic acid and 10 μl of (0.1% formic acid) prior to nanoLC-MS analysis.

### Dimethyl labeling

After digestion, the total protein extract was quantified by the BCA method and the volume was adjusted to 100 μl of 100 mM TEAB. CH_2_O (4 μl, 4%, “light”) or 4% CD_2_O (“intermediate”) or 4% ^13^CD_2_O (“heavy”) was added, followed by the addition of 4 μl of 600 mM NaBH_3_CN (light and intermediate) or 4 μl of 600 mM NaBD_3_CN (heavy). The mixture was incubated for 1 h at room temperature. The reaction was quenched with 16 μl of 1% ammonia and 8 μl formic acid was added. The differentially labeled samples from three different time points were pooled and desalted using microcolumns filled with Poros R3 beads. This sample was subjected to vacuum centrifugation and stored at −20°C for further use.

### Titanium dioxide (TiO_2_) chromatography

The pooled samples were subjected to the phosphoenrichement procedure by mixing with TiO_2_ beads, which were ressuspended in loading buffer (1 M glycolic acid in 80% acetonitrile, 5% TFA). 15 mg of TiO_2_ beads (suspended in 100% acetonitrile) were washed in loading buffer and loaded into the sample tube. The mixture was incubated for 15 min at ambient temperature under agitation (150 rpm). The mixture was centrifuged for 60 s at 12,000 g and the supernatant was collected, dessalted, and lyophilized. The TiO_2_ beads, complexed with phosphopeptides, were washed twice with 500 μl of loading buffer and, subsequently, with 30 μl of washing buffer (80% acetonitrile, 5% TFA). The phosphopeptides were eluted using 50 μl of ammonium water (pH 11.3) followed by 10 μl of 30% acetonitrile. The eluent was acidified by adding 5 μl of 100% formic acid prior to the dessalting step.

### Offline TSK amide-80 HILIC peptide fractionation

Peptide fractionation was performed using a neutral TSK Amide-80 HILIC (3 μm; Tosoh Bioscience, San Francisco, USA) and a mobile phase containing TFA. The purified peptides were ressuspended in 90% acetonitrile, 0.1% TFA and loaded onto a 320 μm inner/450 μm outer diameter × 17 cm microcapillary column packed with TSK Amide-80 using an Agilent 1200 Series HPLC (Agilent, Santa Clara, USA). The HPLC gradient was 100-60% of solvent 90% acetonitrile/0.1% TFA in water for 42 min at a flow-rate of 6 μL/min. Fractions were collected every minute and combined into 8–12 fractions depending on the intensity of UV detection measured at 210.8 nm. The fractions were dried by vacuum centrifugation.

### Nano-LC-MS

Nano-LC-MS experiments were performed using a 7-tesla LTQ-FT mass spectrometer (Thermo Electron, Bremen, Germany). The sample was applied onto an EASY nano-LC system (Proxeon Biosystems, Odense, Denmark). The peptides were loaded onto an 18 cm analytical column (100 μm inner diameter/360 μm outer diameter, ReproSil-Pur C18 AQ 3 μm (Dr. Maisch, Ammerbuch-Entringen, Germany), and eluted from the column using a gradient from 100% phase A (0.5% acetic acid aqueous solution) to 40% phase B (0.5% acetic acid, 80% acetonitrile) in 113 min at 45 nl/min. The instrument was operated in a data-dependent mode automatically switching between MS, MS^2^, and pdMS^3^[55]. The top 10 parent ions of the spectra were chosen for fragmentation. The pdMS^3^ acquisition was set to automatically select and further fragment the fragment ion originating from the loss of phosphoric acid from the parent ions (standard pdMS3 settings).

### Database analysis

The “.raw” MS data were processed using the ThermoProteome Discoverer software (Thermo Electron, Bremen, Germany). The generated “.mgf” files were subsequently searched against the murine sequence library in the International Protein Index (IPI) protein sequence database (IPI mouse v3.77) using an in-house Mascot server (version 2.1) (Matrix Science Ltd. London, UK). The search was performed by choosing trypsin as the enzyme with two miss-cleavages allowed. Carbamidomethyl (Cys), dimethyl labeling for light, medium and heavy modifications of N-terminus and Lys (+28,+32 and +36 Da, respectively) were chosen as the fixed modification. As variable modifications, oxidations (Met) and phosphorylation (Serine, Threonine and Tyrosine, S,T,Y), were chosen. The data were searched with a peptide mass tolerance of 10 ppm and a fragment mass tolerance of 0.8 Da. A concatenated decoy database search was performed in a concatenated decoy mouse database (IPImousedecoy) derived from the IPI mouse database listed above for each of the conditions, and only peptides with up to 1% of False Discovery (FDR) were selected. Dimethyl quantification was performed using Thermo Proteome Discoverer (v1.1) from the extracted chromatograms (XIC’s) obtained. Normalization was achieved using the LOWESS fitting algorithm [24] and protein grouping and statistics were obtained using StatQuant [56]. The phosphopeptide subpopulation were compared to a databasis consisting of motifs for phosphorylation by different kinases in NetworKIN website (http://www.networkin.info/version_2_0/)[57]. Non-phosphorylated population of peptides were classified according to biological process using the Gene Ontology software Blast2go [58] (http://www.blast2go.com). Ingenuity (Ingenuity Systems, Redwood City, USA) was used to investigate protein network interactions. TRED (http://rulai.cshl.edu/cgiin/TRED/tred.cgi/process=home) was used to search for gene targets for transcription factors and JASPAR (http://129.177.120.189/cgibin/jaspar2010/jaspar_db.pl) was used to check for transcription factor binding motifs.

### Total mRNA extraction and purification from rhBMP2-induced msMSC cells

3.10^4^/ cells per ml were seeded onto 100 mm diameter culture plate. After treatment with rhBMP2 (Life Technologies, USA) for different time periods, cells were washed with ice-cold PBSA, and total mRNA was isolated using silica columns from the RNeasy® mini-kit (Qiagen, Hilden, Germany). The mRNA concentration was determined by absorbance at 260 nm and the purity of the preparations was evaluated by the A_260_nm/A_280_nm ratio, with purity being considered when this ratio was approximately 2.0.

### cDNA synthesis

Total cellular RNA, isolated as mentioned above, was used to synthetize the corresponding cDNA. An aliquot (1 μg) of RNA from each condition was incubated with 2 units of DNase I (Thermo Fischer Scientific, Waltham, USA) and 20 units of RNAseOUT for 10 min at 37°C. After this incubation period, both enzymes were heat-inactivated for 10 min at 75°C and 1 μl of 0.5 μg/μl of oligo-dT, 1 μl of 10 mM dNTP, were added. The samples were incubated for 10 min at 65°C and then immediately placed on ice. After addition of 200 units of SuperScript®, 2 μl of 100 mM DTT and 20 units of RNAseOUT were added to each tube, samples were incubated for 10 min at 25°C for primer annealing, and then for 120 min at 50°C for cDNA synthesis. SuperScript® enzyme was heat-inactivated and the template RNA was then degraded upon incubation with 5 units of RNaseH, for 30 min at 37°C.

### Quantitative Real Ttime PCR (qRT-PCR)

The experiments were carried out according to the MIQE guidelines [59]. The first step for the primer selections was to select from already published data a set of genes of interest differentially regulated during osteogenesis [22,60]. The primer sequences were then selected from a validated bank of oligos previously tested and approved for qRT-PCR, the PrimerBank [61]. The primer concentration was then optimized for each gene using a cDNA pool from different periods of time of treatment with BMP2, adopting the lowest primer concentration for each condition that did not interfere with the amplification curve inclination, in order to avoid non-specific results derived from primer dimers. The qRT-PCR reaction was carried out using 6 μl the SYBR® Green Dye (Life Technologies, Carlsbad, USA), 3 μl of 30 times diluted cDNA and 3 μl of a mix containing both the forward and the reverse primers (Table 3), and incubated under the following conditions: 2 min at 50°C, 10 min at 95°C, followed by 40 cycles of 15 seconds at 95°C and 60°C for 1 min. The data were collected and analyzed using the 7300 System Software (Life Technologies, Carlsbad, USA). The quality control of each reaction was achieved through a cycle of dissociation, in order to exclude possible cross-contaminations or the presence of dimers. To confirm the differential expression for each gene, the GeneAmp 5700 software was used, and the threshold was set to 0.1. The data was exported and interpreted using the qBASE^PLUS2^ (Biogazelle, Zwijnaarde, Belgium). The first step was to use the Genorm [62] tool, a very popular algorithm that finds the stablest reference (housekeeping) genes from a set of tested candidate reference genes in a given experimental condition, in this case, GAPDH, HMBS and HPRT. From this, a gene expression normalization factor was calculated for each sample, based on the geometric mean of a user-defined number of the reference genes. After analysis, the data was exported and the graphic pictures and statistical analysis were performed using the GraphPad Prism 5 software (GraphPad Software, La Jolla, USA). The data presented in this work are representative of 3 independent experiments, performed in duplicates, and were analised through a one-way Anova followed by a post-test of Tukey with p<0.005.

## Competing interests

The authors declared that they have no competing interests.

## Authors’ contributions

EH carried out the sample preparation, mass spectrometry and data analysis and drafted the manuscript. MFF carried out the RT-PCR experiments and data analysis and also isolated the cell line used in this work. AF participated in the mass spectrometry data normalization. TVB supported stable isotope dimethyl labeling and mass spectrometry experiments. ONJ participated in the manuscript revision, and revised a. MCS conceived of the study, and participated in its design and coordination and helped to draft the manuscript. All authors read and approved the final manuscript.

## Supplementary Material

Additional file 1 **Phosphorylated peptides found in MS experiments.** Acession number, delta score, retention area (for light medium and heavy labeled peptides) of phosphorylated peptides. Click here for file

Additional file 2 **Phosphorylated proteins found and relative ratio variation for different timepoints of BMP2-induction and calculated p-value for all peptides corresponding to a specific protein.** For a given accession number corresponding to a protein, a subset of peptides were found in MS experiments, and the ratio between each timepoint was calculated using StatQuant software according to the area of extracted chromatogram. Statistical analysis was calculated for a population of peptides corresponding to a same protein accession. Click here for file

Additional file 3 **Phosphorylated sites found in peptides according to MS experiments and kinase phosphorylation analysis using NetworKIN 2.0 Beta (http://www.networkin.info/version_2_0/).** To investigate which kinases could be involved in the phosphorylation of peptides found in MS experiments, NetworKIN 2.0 Beta kinase databank was used to match phoshorylated serine, threonine and tyrosine in peptides found to be phoshorylated, according to prior experiments in literature for a given kinase based on PhosphoELM (http://phospho.elm.eu.org/) and on Phosphosite (http://www.phosphosite.org). Click here for file

Additional file 4 **Network of proteins which were found to interact with phosphorylated proteins found.** The list of phosphoproteins found were subjected to Ingenuity Pathway Analysis (IPA) to investigate probable protein interactions for each cellular compartment. Proteins described to be transcription factors were selected to investigate the activation of osteoblast related genes by quantitative real-time PCR. Click here for file

Additional file 5 **Predicted network of interaction for phosphoproteins found using Ingenuity Pathway Analysis.** Ingenuity Pathway Analysis was used to identify the network of proteins which could interact with the phosphoproteins identified. Dashed arrows represent the predicted interactions and the full arrows represent a confirmed interaction. Proteins were separated by cell compartment and proteins known to be transcription factors were selected to further analysis to investigate possible activators of osteoblast differentiation by real-time quantitative PCR. Click here for file

Additional file 6 **Motifs and Pre-selected genes used in RT-PCR experiments.** The motifs listed were used to search for correspondents in the promoter region of the pre-selected genes. Those which displayed at least one match matches for the sequences (SP1, c-Myc e NF-ƙB) were selected for the qRT-PCR analysis. Click here for file
