# Supplementary material for: New insights in osteogenic differentiation revealed by mass spectrometric assessment of phosphorylated substrates in murine skin mesenchymal cells
Source: BMC Cell Biol. 2013 Oct 22;14:47. doi: 10.1186/1471-2121-14-47 (PMC3819743; doi:10.1186/1471-2121-14-47)
Supplement: Additional file 2 — Phosphorylated proteins found and relative ratio variation for different timepoints of BMP2-induction and calculated p-value for all peptides corresponding to a specific protein. For a given accession number corresponding to a protein, a subset of peptides were found in MS experiments, and the ratio between each timepoint was calculated using StatQuant software according to the area of extracted chromatogram. Statistical analysis was calculated for a population of peptides corresponding to a same protein accession. [file 1471-2121-14-47-S2.docx]

| Protein / Unique Peptides Identified / Ratio / p-value | IPI number | BMP2 induction time / peptides found / ratio | | | |
| --- | --- | --- | --- | --- | --- |
| **Xrn2 5'-3' exoribonuclease 2** | IPI00120046.1 | 10'/0h | 30'/10' | 1h/30' | 2h/1h |
| **peptides identified** |  | 4 | 4 | 2 | 2 |
| **ratio** |  | 0,900 | 1,878 | 1,143 | 1,674 |
| **p-value** |  | 0,024 | 0,000 | 0,556 | 0,147 |
| **DEAD box protein 21 Nucleolar RNA helicase 2** | IPI00120691.3 | 10'/0h | 30'/10' | 1h/30' | 2h/1h |
| **peptides identified** |  | 26 | 26 | 6 | 6 |
| **ratio** |  | 0,608 | 2,411 | 1,227 | 5,071 |
| **p-value** |  | 0,000 | 0,000 | 0,001 | 0,000 |
| **Nuclease-sensitive element-binding protein 1** | IPI00120886.3 | 10'/0h | 30'/10' | 1h/30' | 2h/1h |
| **peptides identified** |  | 2 | 2 | 1 | 1 |
| **ratio** |  | 1,434 | 0,810 | 0,761 | 0,189 |
| **p-value** |  | 0,002 | 0,039 | 1,000 | 1,000 |
| **Isoform 3 of Ribosome-binding protein 1** | IPI00121149.1 |  |  | 1h/30' | 2h/1h |
| **peptides identified** |  |  |  | 7 | 7 |
| **ratio** |  |  |  | 0,774 | 3,230 |
| **p-value** |  |  |  | 0,002 | 0,000 |
| **Thioredoxin-related transmembrane protein 1** | IPI00121341.1 |  |  | 1h/30' | 2h/1h |
| **peptides identified** |  |  |  | 1 | 1 |
| **ratio** |  |  |  | 0,355 | 7,547 |
| **p-value** |  |  |  | 1,000 | 1,000 |
| **GPN-loop GTPase 1** | IPI00122054.1 | 10'/0h | 30'/10' | 1h/30' | 2h/1h |
| **peptides identified** |  | 2 | 2 | 2 | 2 |
| **ratio** |  | 1,294 | 1,266 | 1,081 | 1,360 |
| **p-value** |  | 0,117 | 0,069 | 0,478 | 0,348 |
| **Caldesmon 1** | IPI00122450.1 | 10'/0h | 30'/10' | 1h/30' | 2h/1h |
| **peptides identified** |  | 2 | 2 | 1 | 1 |
| **ratio** |  | 1,094 | 0,643 | 0,830 | 0,591 |
| **p-value** |  | 0,008 | 0,016 | 1,000 | 1,000 |
| **Ser/Thr-Protein Phosphatase 6 Regulatory Subunit 3** | IPI00122858.1 | 10'/0h | 30'/10' |  |  |
| **peptides identified** |  | 3 | 3 |  |  |
| **ratio** |  | 4,631 | 1,538 |  |  |
| **p-value** |  | 0,612 | 0,062 |  |  |
| **Myosin-9** | IPI00123181.4 | 10'/0h | 30'/10' |  |  |
| **peptides identified** |  | 16 | 16 |  |  |
| **ratio** |  | 0,833 | 4,260 |  |  |
| **p-value** |  | 0,000 | 0,000 |  |  |
| **A-kinase anchor protein 12** | IPI00123709.1 | 10'/0h | 30'/10' | 1h/30' | 2h/1h |
| **peptides identified** |  | 6 | 6 | 11 | 11 |
| **ratio** |  | 1,157 | 0,929 | 0,949 | 0,961 |
| **p-value** |  | 0,001 | 0,130 | 0,021 | 0,231 |
| **Chloride channel, nucleotide-sensitive, 1A** | IPI00124248.1 | 10'/0h | 30'/10' | 1h/30' | 2h/1h |
| **peptides identified** |  | 14 | 14 | 4 | 4 |
| **ratio** |  | 0,856 | 1,118 | 1,187 | 0,633 |
| **p-value** |  | 0,000 | 0,000 | 0,001 | 0,000 |
| **of Band 4.1-like protein 3** | IPI00125501.1 | 10'/0h | 30'/10' | 1h/30' | 2h/1h |
| **peptides identified** |  | 2 | 2 | 2 | 2 |
| **ratio** |  | 0,932 | 0,868 | 0,972 | 0,988 |
| **p-value** |  | 0,223 | 0,033 | 0,761 | 0,051 |
| **LAG1 longevity assurance homolog 2** | IPI00126253.1 | 10'/0h | 30'/10' |  |  |
| **peptides identified** |  | 4 | 4 |  |  |
| **ratio** |  | 0,543 | 3,998 |  |  |
| **p-value** |  | 0,000 | 0,000 |  |  |
| **Lamina-associated polypeptide 2, isoforms alpha/zeta** | IPI00126338.5 | 10'/0h | 30'/10' |  |  |
| **peptides identified** |  | 2 | 2 |  |  |
| **ratio** |  | 0,387 | 3,525 |  |  |
| **p-value** |  | 0,007 | 0,001 |  |  |
| **Zinc finger Ran-binding domain-containing protein 2** | IPI00126804.6 | 10'/0h | 30'/10' | 1h/30' | 2h/1h |
| **peptides identified** |  | 2 | 4 | 5 | 5 |
| **ratio** |  | 1,155 | 0,672 | 1,356 | 0,732 |
| **p-value** |  | 0,017 | 0,000 | 0,000 | 0,000 |
| **Small acidic protein** | IPI00127941.1 | 10'/0h | 30'/10' | 1h/30' | 2h/1h |
| **peptides identified** |  | 6 | 6 | 3 | 3 |
| **ratio** |  | 1,114 | 1,041 | 0,855 | 0,677 |
| **p-value** |  | 0,168 | 0,188 | 0,171 | 0,007 |
| **Prostaglandin E synthase 3** | IPI00127989.1 | 10'/0h | 30'/10' | 1h/30' | 2h/1h |
| **peptides identified** |  | 10 | 10 | 3 | 3 |
| **ratio** |  | 0,852 | 1,022 | 1,796 | 0,890 |
| **p-value** |  | 0,000 | 0,045 | 0,001 | 0,061 |
| **of General vesicular transport factor p115** | IPI00128071.7 | 10'/0h | 30'/10' |  |  |
| **peptides identified** |  | 2 | 2 |  |  |
| **ratio** |  | 0,920 | 2,933 |  |  |
| **p-value** |  | 0,499 | 0,075 |  |  |
| **Ras GTPase-activating protein-binding protein 1** | IPI00130095.1 | 10'/0h | 30'/10' | 1h/30' | 2h/1h |
| **peptides identified** |  | 8 | 8 | 4 | 4 |
| **ratio** |  | 1,115 | 0,923 | 2,151 | 1,009 |
| **p-value** |  | 0,000 | 0,000 | 0,000 | 0,151 |

| Protein / Unique Peptides Identified / Ratio / p-value | IPI number | BMP2 induction time | | | |
| --- | --- | --- | --- | --- | --- |
| **Bag Family Molecular Chaperone Regulator 2** | IPI00130304.1 |  |  | 1h/30' | 2h/1h |
| **peptides identified** |  |  |  | 1 | 1 |
| **ratio** |  |  |  | 1,516 | 0,512 |
| **p-value** |  |  |  | 1,000 | 1,000 |
| **Microtubule-associated protein 1B** | IPI00130920.1 | 10'/0h | 30'/10' |  |  |
| **peptides identified** |  | 3 | 3 |  |  |
| **ratio** |  | 2,004 | 5,437 |  |  |
| **p-value** |  | 0,964 | 0,583 |  |  |
| **Calcium-regulated heat stable protein 1** | IPI00133349.1 |  |  | 1h/30' | 2h/1h |
| **peptides identified** |  |  |  | 3 | 3 |
| **ratio** |  |  |  | 0,996 | 0,671 |
| **p-value** |  |  |  | 0,000 | 0,000 |
| **DNA Topoisomerase 2-beta** | IPI00135443.2 | 10'/0h | 30'/10' |  |  |
| **peptides identified** |  | 2 | 2 |  |  |
| **ratio** |  | 0,051 | 26,138 |  |  |
| **p-value** |  | 0,000 | 0,000 |  |  |
| **Monocarboxylate Transporter 1** | IPI00137194.1 |  |  | 1h/30' | 2h/1h |
| **peptides identified** |  |  |  | 3 | 3 |
| **ratio** |  |  |  | 1,257 | 1,489 |
| **p-value** |  |  |  | 0,284 | 0,019 |
| **60S acidic ribosomal protein P2** | IPI00139795.2 | 10'/0h | 30'/10' | 1h/30' | 2h/1h |
| **peptides identified** |  | 12 | 12 | 4 | 4 |
| **ratio** |  | 0,712 | 1,115 | 0,878 | 0,726 |
| **p-value** |  | 0,000 | 0,000 | 0,007 | 0,000 |
| **Cytoplasmic dynein 1 light intermediate chain 1** | IPI00153421.1 | 10'/0h | 30'/10' | 1h/30' | 2h/1h |
| **peptides identified** |  | 2 | 2 | 2 | 2 |
| **ratio** |  | 0,668 | 1,027 | 1,079 | 1,276 |
| **p-value** |  | 0,046 | 0,219 | 0,578 | 0,181 |
| **Ataxin-2-like protein** | IPI00169500.3 | 10'/0h | 30'/10' |  |  |
| **peptides identified** |  | 2 | 2 |  |  |
| **ratio** |  | 1,207 | 0,737 |  |  |
| **p-value** |  | 0,233 | 0,157 |  |  |
| **Periphilin-1** | IPI00169907.1 | 10'/0h | 30'/10' | 1h/30' | 2h/1h |
| **peptides identified** |  | 6 | 6 | 3 | 3 |
| **ratio** |  | 1,094 | 0,722 | 1,217 | 0,472 |
| **p-value** |  | 0,004 | 0,000 | 0,099 | 0,000 |
| **Eukaryotic translation initiation factor 4B** | IPI00221581.1 | 10'/0h | 30'/10' | 1h/30' | 2h/1h |
| **peptides identified** |  | 52 | 52 | 22 | 22 |
| **ratio** |  | 1,007 | 0,803 | 0,922 | 0,573 |
| **p-value** |  | 0,947 | 0,000 | 0,009 | 0,000 |
| **Charged Multivesicular Body Protein 2B** | IPI00222386.3 | 10'/0h | 30'/10' |  |  |
| **peptides identified** |  | 2 | 2 |  |  |
| **ratio** |  | 1,019 | 1,275 |  |  |
| **p-value** |  | 0,876 | 0,261 |  |  |
| **Heterogeneous nuclear ribonucleoprotein K** | IPI00223253.1 | 10'/0h | 30'/10' | 1h/30' | 2h/1h |
| **peptides identified** |  | 4 | 4 | 2 | 2 |
| **ratio** |  | 1,651 | 0,989 | 1,654 | 0,857 |
| **p-value** |  | 0,001 | 0,690 | 0,049 | 0,047 |
| **Glycylpeptide N-tetradecanoyltransferase 1** | IPI00224128.7 |  |  | 1h/30' | 2h/1h |
| **peptides identified** |  |  |  | 2 | 2 |
| **ratio** |  |  |  | 0,884 | 1,055 |
| **p-value** |  |  |  | 0,598 | 0,517 |
| **Protein IWS1 homolog** | IPI00224200.1 | 10'/0h | 30'/10' |  |  |
| **peptides identified** |  | 2 | 2 |  |  |
| **ratio** |  | 1,155 | 0,736 |  |  |
| **p-value** |  | 0,130 | 0,021 |  |  |
| **Matrix-Remodeling-Associated Protein 7** | IPI00226263.5 | 10'/0h | 30'/10' | 1h/30' | 2h/1h |
| **peptides identified** |  | 6 | 6 | 2 | 2 |
| **ratio** |  | 1,330 | 0,879 | 0,507 | 2,683 |
| **p-value** |  | 0,001 | 0,015 | 0,046 | 0,002 |
| **Vimentin** | IPI00227299.6 | 10'/0h | 30'/10' | 1h/30' | 2h/1h |
| **peptides identified** |  | 2 | 2 | 2 | 2 |
| **ratio** |  | 0,947 | 1,015 | 2,676 | 0,721 |
| **p-value** |  | 0,359 | 0,467 | 0,250 | 0,160 |
| **Microfibrilar-Associated Protein 1** | IPI00228590.2 | 10'/0h | 30'/10' |  |  |
| **peptides identified** |  | 2 | 2 |  |  |
| **ratio** |  | 0,819 | 0,724 |  |  |
| **p-value** |  | 0,019 | 0,013 |  |  |
| **Isoform 3 of Band 4.1-like protein 3** | IPI00229295.1 | 10'/0h | 30'/10' |  |  |
| **peptides identified** |  | 2 | 2 |  |  |
| **ratio** |  | 1,201 | 0,962 |  |  |
| **p-value** |  | 0,166 | 0,381 |  |  |
| **Myristoylated alanine-rich C-kinase substrate** | IPI00229534.5 | 10'/0h | 30'/10' | 1h/30' | 2h/1h |
| **peptides identified** |  | 29 | 29 | 19 | 19 |
| **ratio** |  | 8,410 | 0,648 | 1,036 | 0,727 |
| **p-value** |  | 0,006 | 0,000 | 0,401 | 0,000 |

| Protein / Unique Peptides Identified / Ratio / p-value | IPI number | BMP2 induction time | | | |
| --- | --- | --- | --- | --- | --- |
| **Eukaryotic translation initiation factor 3 subunit B** | IPI00229859.1 | 10'/0h | 30'/10' | 1h/30' | 2h/1h |
| **peptides identified** |  | 8 | 8 | 6 | 6 |
| **ratio** |  | 1,641 | 1,460 | 1,260 | 1,571 |
| **p-value** |  | 0,133 | 0,000 | 0,012 | 0,015 |
| **Plectin** | IPI00230061.2 | 10'/0h | 30'/10' | 1h/30' | 2h/1h |
| **peptides identified** |  | 4 | 4 | 4 | 4 |
| **ratio** |  | 1,229 | 1,507 | 1,138 | 1,740 |
| **p-value** |  | 0,000 | 0,000 | 0,018 | 0,000 |
| **Histone H1.5** | IPI00230133.5 | 10'/0h | 30'/10' | 1h/30' | 2h/1h |
| **peptides identified** |  | 10 | 10 | 5 | 5 |
| **ratio** |  | 0,623 | 0,540 | 0,662 | 3,064 |
| **p-value** |  | 0,000 | 0,000 | 0,000 | 0,000 |
| **Importin subunit alpha-3** | IPI00230429.4 | 10'/0h | 30'/10' | 1h/30' | 2h/1h |
| **peptides identified** |  | 6 | 6 | 5 | 5 |
| **ratio** |  | 0,917 | 1,662 | 1,799 | 1,725 |
| **p-value** |  | 0,014 | 0,000 | 0,000 | 0,000 |
| **Nuclear Mitotic Apparatus Protein 1** | IPI00263048.2 | 10'/0h | 30'/10' | 1h/30' | 2h/1h |
| **peptides identified** |  | 2 | 2 | 2 | 2 |
| **ratio** |  | 0,525 | 2,555 | 1,699 | 1,912 |
| **p-value** |  | 0,013 | 0,000 | 0,431 | 0,160 |
| **Marcks-Related Protein** | IPI00281011.7 | 10'/0h | 30'/10' | 1h/30' | 2h/1h |
| **peptides identified** |  | 4 | 4 | 3 | 3 |
| **ratio** |  | 0,875 | 0,623 | 0,933 | 0,400 |
| **p-value** |  | 0,040 | 0,001 | 0,000 | 0,000 |
| **Arginine/Serine-Rich Splicing Factor 6** | IPI00310880.4 | 10'/0h | 30'/10' |  |  |
| **peptides identified** |  | 2 | 2 |  |  |
| **ratio** |  | 0,958 | 0,682 |  |  |
| **p-value** |  | 0,028 | 0,018 |  |  |
| **Leiomodin 1** | IPI00311422.5 |  |  | 1h/30' | 2h/1h |
| **peptides identified** |  |  |  | 1 | 1 |
| **ratio** |  |  |  | 0,450 | 1,432 |
| **p-value** |  |  |  | 1,000 | 1,000 |
| **Hepatoma-Derived Growth Factor** | IPI00313817.1 | 10'/0h | 30'/10' | 1h/30' | 2h/1h |
| **peptides identified** |  | 10 | 10 | 7 | 7 |
| **ratio** |  | 0,831 | 1,017 | 0,960 | 0,628 |
| **p-value** |  | 0,003 | 0,756 | 0,387 | 0,006 |
| **High Mobility Group Protein HMG-I** | IPI00314240.5 | 10'/0h | 30'/10' | 1h/30' | 2h/1h |
| **peptides identified** |  | 22 | 22 | 15 | 15 |
| **ratio** |  | 0,608 | 1,995 | 0,648 | 1,475 |
| **p-value** |  | 0,000 | 0,000 | 0,000 | 0,000 |
| **Hematologial and Neurological Expressed Protein1** | IPI00314755.5 | 10'/0h | 30'/10' | 1h/30' | 2h/1h |
| **peptides identified** |  | 12 | 12 | 6 | 6 |
| **ratio** |  | 0,850 | 0,819 | 0,582 | 0,524 |
| **p-value** |  | 0,000 | 0,000 | 0,000 | 0,000 |
| **60S Acidic Ribosomal Protein P0** | IPI00314950.2 | 10'/0h | 30'/10' | 1h/30' | 2h/1h |
| **peptides identified** |  | 10 | 10 | 11 | 11 |
| **ratio** |  | 0,940 | 2,010 | 1,132 | 2,510 |
| **p-value** |  | 0,026 | 0,000 | 0,132 | 0,000 |
| **Nucleolin** | IPI00317794.5 | 10'/0h | 30'/10' | 1h/30' | 2h/1h |
| **peptides identified** |  | 26 | 26 | 9 | 9 |
| **ratio** |  | 0,909 | 1,497 | 1,278 | 0,711 |
| **p-value** |  | 0,022 | 0,006 | 0,000 | 0,000 |
| **Nucleolar Protein 56** | IPI00318048.5 | 10'/0h | 30'/10' | 1h/30' | 2h/1h |
| **peptides identified** |  | 10 | 10 | 1 | 1 |
| **ratio** |  | 0,734 | 1,989 | 1,340 | 2,361 |
| **p-value** |  | 0,000 | 0,000 | 1,000 | 1,000 |
| **Spectrin Beta Chain 1** | IPI00319830.7 | 10'/0h | 30'/10' | 1h/30' | 2h/1h |
| **peptides identified** |  | 4 | 4 | 2 | 2 |
| **ratio** |  | 1,088 | 1,154 | 1,379 | 1,520 |
| **p-value** |  | 0,211 | 0,003 | 0,029 | 0,030 |
| **Membrane Associated Progesterone Receptor Component 1** | IPI00319973.3 | 10'/0h | 30'/10' | 1h/30' | 2h/1h |
| **peptides identified** |  | 36 | 36 | 10 | 10 |
| **ratio** |  | 1,629 | 1,364 | 0,724 | 2,751 |
| **p-value** |  | 0,000 | 0,000 | 0,000 | 0,000 |
| **Elongation Factor 1 beta** |  | 10'/0h | 30'/10' | 1h/30' | 2h/1h |
| **peptides identified** |  | 26 | 26 | 11 | 11 |
| **ratio** |  | 0,634 | 1,480 | 0,890 | 0,957 |
| **p-value** |  | 0,000 | 0,000 | 0,000 | 0,000 |
| **Eukaryotic Translation Factor 3 Subnunit C** | IPI00321647.2 |  |  | 1h/30' | 2h/1h |
| **peptides identified** |  |  |  | 1 | 1 |
| **ratio** |  |  |  | 1,326 | 1,726 |
| **p-value** |  |  |  | 1,000 | 1,000 |
| **Transcriptional Regulator ATRX** | IPI00322707.5 | 10'/0h | 30'/10' | 1h/30' | 2h/1h |
| **peptides identified** |  | 2 | 2 | 1 | 1 |
| **ratio** |  | 0,582 | 1,834 | 1,024 | 1,131 |
| **p-value** |  | 0,001 | 0,016 | 1,000 | 1,000 |

| Protein / Unique Peptides Identified / Ratio / p-value | IPI number | BMP2 induction time | | | |
| --- | --- | --- | --- | --- | --- |
| **Putative Uncharacterized Protein** | IPI00323820.4 | 10'/0h | 30'/10' |  |  |
| **peptides identified** |  | 9 | 9 |  |  |
| **ratio** |  | 0,926 | 4,115 |  |  |
| **p-value** |  | 0,355 | 0,016 |  |  |
| **Pleckstrin Homology-Like Domain Family B Member 2** | IPI00330773.3 | 10'/0h | 30'/10' | 1h/30' | 2h/1h |
| **peptides identified** |  | 4 | 4 | 2 | 2 |
| **ratio** |  | 1,136 | 0,853 | 1,093 | 0,606 |
| **p-value** |  | 0,087 | 0,066 | 0,274 | 0,054 |
| **Myb-Binding Protein 1A** | IPI00331361.2 | 10'/0h | 30'/10' | 1h/30' | 2h/1h |
| **peptides identified** |  | 8 | 8 | 5 | 5 |
| **ratio** |  | 0,635 | 1,982 | 0,837 | 3,515 |
| **p-value** |  | 0,000 | 0,000 | 0,290 | 0,001 |
| **High Mobility Group Protein HMGI-C** | IPI00331612.3 | 10'/0h | 30'/10' | 1h/30' | 2h/1h |
| **peptides identified** |  | 32 | 32 | 10 | 10 |
| **ratio** |  | 0,870 | 1,268 | 0,390 | 3,671 |
| **p-value** |  | 0,000 | 0,000 | 0,001 | 0,000 |
| **Proteasome Subunit Alpha Type-3** | IPI00331644.5 | 10'/0h | 30'/10' | 1h/30' | 2h/1h |
| **peptides identified** |  | 23 | 23 | 10 | 10 |
| **ratio** |  | 1,334 | 1,844 | 0,995 | 1,935 |
| **p-value** |  | 0,020 | 0,000 | 0,589 | 0,000 |
| **Non-Histone Chromosomal Protein HMG-14** | IPI00338745.4 | 10'/0h | 30'/10' | 1h/30' | 2h/1h |
| **peptides identified** |  | 2 | 2 | 1 | 1 |
| **ratio** |  | 1,241 | 0,759 | 0,734 | 0,136 |
| **p-value** |  | 0,080 | 0,019 | 1,000 | 1,000 |
| **La-Related Protein 7** | IPI00340860.5 | 10'/0h | 30'/10' | 1h/30' | 2h/1h |
| **peptides identified** |  | 2 | 2 | 2 | 2 |
| **ratio** |  | 1,126 | 0,616 | 1,546 | 0,930 |
| **p-value** |  | 0,000 | 0,015 | 0,000 | 0,000 |
| **Nuclear Ubiquitous Casein Kinase Substrate** | IPI00341869.5 | 10'/0h | 30'/10' | 1h/30' | 2h/1h |
| **peptides identified** |  | 26 | 26 | 21 | 21 |
| **ratio** |  | 0,842 | 0,866 | 0,917 | 0,544 |
| **p-value** |  | 0,000 | 0,000 | 0,043 | 0,000 |
| **28 Kda Heat- and Acid-Stable Phosphoprotein** | IPI00352475.3 | 10'/0h | 30'/10' | 1h/30' | 2h/1h |
| **peptides identified** |  | 30 | 30 | 7 | 7 |
| **ratio** |  | 1,079 | 0,962 | 0,911 | 0,573 |
| **p-value** |  | 0,003 | 0,065 | 0,001 | 0,000 |
| **Fermrhogef (Arhgef) and Pleckstrin Domain Protein 1** | IPI00356904.7 | 10'/0h | 30'/10' | 1h/30' | 2h/1h |
| **peptides identified** |  | 2 | 2 | 2 | 2 |
| **ratio** |  | 1,550 | 0,939 | 2,835 | 0,889 |
| **p-value** |  | 0,091 | 0,447 | 0,125 | 0,455 |
| **Tensin 1** | IPI00378438.6 | 10'/0h | 30'/10' | 1h/30' | 2h/1h |
| **peptides identified** |  | 2 | 2 | 2 | 2 |
| **ratio** |  | 1,938 | 1,409 | 1,101 | 1,355 |
| **p-value** |  | 0,015 | 0,003 | 0,000 | 0,000 |
| **ATP-Binding Cassette Sub-Family F Member 1** | IPI00396671.1 | 10'/0h | 30'/10' |  |  |
| **peptides identified** |  | 4 | 4 |  |  |
| **ratio** |  | 0,514 | 3,409 |  |  |
| **p-value** |  | 0,030 | 0,001 |  |  |
| **Nexilin** | IPI00400168.2 | 10'/0h | 30'/10' | 1h/30' | 2h/1h |
| **peptides identified** |  | 34 | 34 | 15 | 15 |
| **ratio** |  | 1,5674 | 0,7989 | 0,6361 | 0,9266 |
| **p-value** |  | 0,0000 | 0,0000 | 0,0000 | 0,1134 |
| **Pleckstrin Homology Domain-Containing Family O,2** | IPI00403031.5 | 10'/0h | 30'/10' |  |  |
| **peptides identified** |  | 2 | 2 |  |  |
| **ratio** |  | 1,900 | 0,887 |  |  |
| **p-value** |  | 0,029 | 0,052 |  |  |
| **Rho GTPase-Activating Protein 1** | IPI00404970.2 | 10'/0h | 30'/10' | 1h/30' | 2h/1h |
| **peptides identified** |  | 2 | 2 | 2 | 2 |
| **ratio** |  | 1,307 | 0,905 | 1,000 | 1,590 |
| **p-value** |  | 0,010 | 0,133 | 0,983 | 0,015 |
| **Microtubule-Associated Protein 4** | IPI00408119.6 | 10'/0h | 30'/10' | 1h/30' | 2h/1h |
| **peptides identified** |  | 6 | 6 | 2 | 2 |
| **ratio** |  | 1,124 | 0,678 | 1,100 | 0,690 |
| **p-value** |  | 0,250 | 0,000 | 0,344 | 0,023 |
| **Bcl-2-Associated Transcription Factor 1** | IPI00415385.1 | 10'/0h | 30'/10' | 1h/30' | 2h/1h |
| **peptides identified** |  | 16 | 16 | 6 | 6 |
| **ratio** |  | 1,027 | 0,650 | 2,060 | 0,646 |
| **p-value** |  | 0,016 | 0,000 | 0,001 | 0,002 |
| **Ubiquitin Carboxyl-Terminal Hydrolase 10** | IPI00420601.5 | 10'/0h | 30'/10' |  |  |
| **peptides identified** |  | 2 | 2 |  |  |
| **ratio** |  | 0,615 | 1,057 |  |  |
| **p-value** |  | 0,116 | 0,797 |  |  |
| **Eukaryotic Translation Initiation Factor 4 Gamma 1** | IPI00421179.1 |  |  | 1h/30' | 2h/1h |
| **peptides identified** |  |  |  | 1 | 1 |
| **ratio** |  |  |  | 1,028 | 2,138 |
| **p-value** |  |  |  | 1,000 | 1,000 |

| Protein / Unique Peptides Identified / Ratio / p-value | IPI number | BMP2 induction time | | | |
| --- | --- | --- | --- | --- | --- |
| **Swi5-Dependent Recombination DNA Repair Homolog** | IPI00458153.2 | 10'/0h | 30'/10' | 1h/30' | 2h/1h |
| **peptides identified** |  | 26 | 26 | 6 | 6 |
| **ratio** |  | 0,822 | 1,251 | 1,346 | 0,572 |
| **p-value** |  | 0,003 | 0,044 | 0,000 | 0,000 |
| **182 Kda Tankyrase-1-Binding Protein** | IPI00459443.5 | 10'/0h | 30'/10' | 1h/30' | 2h/1h |
| **peptides identified** |  | 4 | 4 | 3 | 3 |
| **ratio** |  | 1,256 | 1,094 | 1,118 | 0,830 |
| **p-value** |  | 0,000 | 0,001 | 0,080 | 0,019 |
| **E3 Ubiquitin-Protein Ligase Nedd4** | IPI00462445.2 | 10'/0h | 30'/10' |  |  |
| **peptides identified** |  | 4 | 4 |  |  |
| **ratio** |  | 5,416 | 2,364 |  |  |
| **p-value** |  | 0,184 | 0,011 |  |  |
| **Ferm Domain-Containing Protein 4a** | IPI00465706.2 | 10'/0h | 30'/10' |  |  |
| **peptides identified** |  | 4 | 4 |  |  |
| **ratio** |  | 1,629 | 0,903 |  |  |
| **p-value** |  | 0,128 | 0,217 |  |  |
| **Sh3 Domain-Containing Kinase-Binding Protein 1** | IPI00466258.2 |  |  | 1h/30' | 2h/1h |
| **peptides identified** |  |  |  | 1 | 1 |
| **ratio** |  |  |  | 0,805 | 0,367 |
| **p-value** |  |  |  | 1,000 | 1,000 |
| **Isoform 2 of DNA (Cytosine-5)-Methyltransferase 1** | IPI00469323.1 | 10'/0h | 30'/10' |  |  |
| **peptides identified** |  | 2 | 2 |  |  |
| **ratio** |  | 0,624 | 3,016 |  |  |
| **p-value** |  | 0,045 | 0,007 |  |  |
| **Ahnak Nucleoprotein** | IPI00553798.2 | 10'/0h | 30'/10' | 1h/30' | 2h/1h |
| **peptides identified** |  | 103 | 103 | 46 | 46 |
| **ratio** |  | 0,880 | 1,450 | 1,474 | 0,715 |
| **p-value** |  | 0,000 | 0,020 | 0,000 | 0,000 |
| **Heat Shock Protein Hsp 90-Beta** | IPI00554929.2 | 10'/0h | 30'/10' | 1h/30' | 2h/1h |
| **peptides identified** |  | 46 | 46 | 19 | 19 |
| **ratio** |  | 0,800 | 1,575 | 1,320 | 1,173 |
| **p-value** |  | 0,000 | 0,000 | 0,000 | 0,000 |
| **Thyroid Hormone Receptor-Associated Protein 3** | IPI00556768.1 | 10'/0h | 30'/10' | 1h/30' | 2h/1h |
| **peptides identified** |  | 4 | 4 | 3 | 3 |
| **ratio** |  | 1,000 | 0,705 | 1,686 | 0,664 |
| **p-value** |  | 0,870 | 0,000 | 0,005 | 0,000 |
| **Serine/Arginine Repetitive Matrix Protein 1** | IPI00605037.1 | 10'/0h | 30'/10' | 1h/30' | 2h/1h |
| **peptides identified** |  | 10 | 18 | 5 | 5 |
| **ratio** |  | 0,912 | 0,605 | 1,659 | 0,565 |
| **p-value** |  | 0,022 | 0,000 | 0,000 | 0,013 |
| **Isoform A of Prelamin-A/C** | IPI00620256.3 | 10'/0h | 30'/10' | 1h/30' | 2h/1h |
| **peptides identified** |  | 42 | 42 | 29 | 29 |
| **ratio** |  | 1,234 | 1,037 | 1,118 | 0,669 |
| **p-value** |  | 0,000 | 0,230 | 0,000 | 0,000 |
| **Isoform 2 of Yorkie Homolog** | IPI00621717.2 | 10'/0h | 30'/10' |  |  |
| **peptides identified** |  | 2 | 2 |  |  |
| **ratio** |  | 1,503 | 0,485 |  |  |
| **p-value** |  | 0,003 | 0,009 |  |  |
| **FACT COMPLEX SUBUNIT SSRP1.** | IPI00652593.2 | 10'/0h | 30'/10' | 1h/30' | 2h/1h |
| **peptides identified** |  | 12 | 12 | 1 | 1 |
| **ratio** |  | 0,931 | 1,589 | 1,492 | 1,172 |
| **p-value** |  | 0,031 | 0,000 | 1,000 | 1,000 |
| **Leucine-Rich Repeat Flightless-Interacting Protein 1** | IPI00654388.2 | 10'/0h | 30'/10' | 1h/30' | 2h/1h |
| **peptides identified** |  | 10 | 10 | 3 | 3 |
| **ratio** |  | 1,025 | 0,741 | 1,171 | 0,723 |
| **p-value** |  | 0,000 | 0,000 | 0,045 | 0,090 |
| **Chromobox Homolog 3** |  | 10'/0h | 30'/10' | 1h/30' | 2h/1h |
| **peptides identified** | IPI00677454.2 | 28 | 28 | 13 | 13 |
| **ratio** |  | 0,934 | 0,883 | 1,148 | 0,532 |
| **p-value** |  | 0,000 | 0,000 | 0,000 | 0,000 |
| **Nucleolar and Coiled-Body Phosphoprotein 1 Isoform D** | IPI00719871.1 | 10'/0h | 30'/10' | 1h/30' | 2h/1h |
| **peptides identified** |  | 2 | 2 | 3 | 3 |
| **ratio** |  | 0,573 | 1,537 | 0,947 | 3,516 |
| **p-value** |  | 0,020 | 0,004 | 0,063 | 0,000 |
| **Mitochondrial Import Receptor Subunit Tom22 Homolog** | IPI00751137.1 | 10'/0h | 30'/10' |  |  |
| **peptides identified** |  | 2 | 2 |  |  |
| **ratio** |  | 1,379 | 2,513 |  |  |
| **p-value** |  | 0,016 | 0,003 |  |  |
| **Eukaryotic Translation Initiation Factor 5b** | IPI00756424.3 | 10'/0h | 30'/10' | 1h/30' | 2h/1h |
| **peptides identified** |  | 18 | 18 | 8 | 8 |
| **ratio** |  | 0,890 | 1,547 | 1,045 | 2,503 |
| **p-value** |  | 0,003 | 0,000 | 0,012 | 0,000 |
| **of Lim Domain and Actin-Binding Protein, beta** | IPI00759925.1 | 10'/0h | 30'/10' | 1h/30' | 2h/1h |
| **peptides identified** |  | 22 | 22 | 10 | 10 |
| **ratio** |  | 1,456 | 0,786 | 0,861 | 1,010 |
| **p-value** |  | 0,000 | 0,000 | 0,000 | 0,723 |

| Protein / Unique Peptides Identified / Ratio / p-value | IPI number | BMP2 induction time | | | |
| --- | --- | --- | --- | --- | --- |
| **Ubiquitin-Associated Protein 2-Like** | IPI00761937.1 | 10'/0h | 30'/10' |  |  |
| **peptides identified** |  | 2 | 2 |  |  |
| **ratio** |  | 1,256 | 0,649 |  |  |
| **p-value** |  | 0,009 | 0,027 |  |  |
| **ATP-Citrate Synthase** | IPI00762047.1 |  |  | 1h/30' | 2h/1h |
| **peptides identified** |  |  |  | 1 | 1 |
| **ratio** |  |  |  | 0,878 | 1,005 |
| **p-value** |  |  |  | 1,000 | 1,000 |
| **Cordon-Bleu Protein-Like 1** | IPI00762331.2 |  |  | 1h/30' | 2h/1h |
| **peptides identified** |  |  |  | 2 | 2 |
| **ratio** |  |  |  | 2,217 | 0,637 |
| **p-value** |  |  |  | 0,000 | 0,000 |
| **ATP-Binding Cassette Sub-Family F Member 1** | IPI00762360.1 |  |  | 1h/30' | 2h/1h |
| **peptides identified** |  |  |  | 7 | 7 |
| **ratio** |  |  |  | 0,922 | 3,333 |
| **p-value** |  |  |  | 0,335 | 0,001 |
| **Serine/Arginine Repetitive Matrix Protein 2** | IPI00785240.1 | 10'/0h | 30'/10' | 1h/30' | 2h/1h |
| **peptides identified** |  | 16 | 16 | 6 | 6 |
| **ratio** |  | 1,216 | 0,721 | 1,623 | 0,541 |
| **p-value** |  | 0,001 | 0,000 | 0,008 | 0,018 |
| **Heterogeneous Nuclear Ribonucleoprotein A1** | IPI00817004.1 | 10'/0h | 30'/10' | 1h/30' | 2h/1h |
| **peptides identified** |  | 10 | 10 | 3 | 3 |
| **ratio** |  | 0,905 | 0,976 | 1,233 | 0,630 |
| **p-value** |  | 0,008 | 0,092 | 0,002 | 0,007 |
| **Lamina-Associated Polypeptide 2 Isoform Delta** | IPI00828461.1 | 10'/0h | 30'/10' | 1h/30' | 2h/1h |
| **peptides identified** |  | 8 | 8 | 2 | 2 |
| **ratio** |  | 0,575 | 2,776 | 0,624 | 12,087 |
| **p-value** |  | 0,000 | 0,000 | 0,033 | 0,004 |
| **Acidic Leucine-Rich Nuclear Phosphoprotein 32, B** | IPI00831258.1 |  |  | 1h/30' | 2h/1h |
| **peptides identified** |  |  |  | 6 | 6 |
| **ratio** |  |  |  | 1,038 | 0,851 |
| **p-value** |  |  |  | 0,007 | 0,000 |
| **TGF Beta-1-Induced Transcript 1 Protein** | IPI00850187.1 |  |  | 1h/30' | 2h/1h |
| **peptides identified** |  |  |  | 3 | 3 |
| **ratio** |  |  |  | 0,522 | 0,676 |
| **p-value** |  |  |  | 0,000 | 0,015 |
| **Uncharacterized Protein** | IPI00850843.2 |  |  | 1h/30' | 2h/1h |
| **peptides identified** |  |  |  | 1 | 1 |
| **ratio** |  |  |  | 1,124 | 0,914 |
| **p-value** |  |  |  | 1,000 | 1,000 |
| **Protein Dpy-19 Homolog 1** | IPI00857720.1 | 10'/0h | 30'/10' |  |  |
| **peptides identified** |  | 1 | 1 |  |  |
| **ratio** |  | 0,077 | 18,666 |  |  |
| **p-value** |  | 1,000 | 1,000 |  |  |
| **Palladin** | IPI00858000.1 | 10'/0h | 30'/10' | 1h/30' | 2h/1h |
| **peptides identified** |  | 16 | 16 | 6 | 6 |
| **ratio** |  | 1,479 | 1,289 | 0,841 | 0,696 |
| **p-value** |  | 0,000 | 0,000 | 0,000 | 0,000 |
| **Heterogeneous Nuclear Ribonucleoprotein C2** | IPI00874321.1 | 10'/0h | 30'/10' |  |  |
| **peptides identified** |  | 1 | 1 |  |  |
| **ratio** |  | 0,132 | 13,642 |  |  |
| **p-value** |  | 1,000 | 1,000 |  |  |
| **Hepatoma-Derived Growth Factor-Related Protein 2** | IPI00875101.1 | 10'/0h | 30'/10' | 1h/30' | 2h/1h |
| **peptides identified** |  | 22 | 22 | 4 | 4 |
| **ratio** |  | 1,137 | 0,739 | 1,531 | 0,738 |
| **p-value** |  | 0,000 | 0,000 | 0,003 | 0,000 |
| **Probable ATP-Dependent Rna Helicase Ddx10** | IPI00896604.1 | 10'/0h | 30'/10' | 1h/30' | 2h/1h |
| **peptides identified** |  | 2 | 2 | 1 | 1 |
| **ratio** |  | 0,620 | 2,217 | 1,328 | 2,210 |
| **p-value** |  | 0,000 | 0,011 | 1,000 | 1,000 |
| **Protein Prrc2c** | IPI00903422.1 |  |  | 1h/30' | 2h/1h |
| **peptides identified** |  |  |  | 2 | 2 |
| **ratio** |  |  |  | 1,314 | 0,737 |
| **p-value** |  |  |  | 0,000 | 0,000 |
| **Tyrosine-Protein Kinase Baz1b** | IPI00923656.1 | 10'/0h | 30'/10' | 1h/30' | 2h/1h |
| **peptides identified** |  | 2 | 2 | 2 | 2 |
| **ratio** |  | 0,376 | 5,182 | 0,771 | 3,806 |
| **p-value** |  | 0,093 | 0,133 | 0,269 | 0,103 |
| **La-Related Protein 1** | IPI00929786.1 | 10'/0h | 30'/10' |  |  |
| **peptides identified** |  | 2 | 2 |  |  |
| **ratio** |  | 1,235 | 1,511 |  |  |
| **p-value** |  | 0,364 | 0,410 |  |  |
| **High Mobility Group Protein Hmg-I/Hmg-Y Isoform C** | IPI00954313.1 | 10'/0h | 30'/10' |  |  |
| **peptides identified** |  | 8 | 8 |  |  |
| **ratio** |  | 0,772 | 1,276 |  |  |
| **p-value** |  | 0,000 | 0,003 |  |  |

**Additional File 2. Phosphorylated proteins found and relative ratio variation for different timepoints of BMP2-induction and calculated p-value for all peptides corresponding to a specific protein.** For a given accession number corresponding to a protein, a subset of peptides were found in MS experiments, and the ratio between each timepoint was calculated using StatQuant software according to the area of extracted chromatogram. Statistical analysis was calculated for a population of peptides corresponding to a same protein accession.
