# Supplementary material for: New insights in osteogenic differentiation revealed by mass spectrometric assessment of phosphorylated substrates in murine skin mesenchymal cells
Source: BMC Cell Biol. 2013 Oct 22;14:47. doi: 10.1186/1471-2121-14-47 (PMC3819743; doi:10.1186/1471-2121-14-47)
Supplement: Additional file 3 — Phosphorylated sites found in peptides according to MS experiments and kinase phosphorylation analysis using NetworKIN 2.0 Beta (http://www.networkin.info/version_2_0/). To investigate which kinases could be involved in the phosphorylation of peptides found in MS experiments, NetworKIN 2.0 Beta kinase databank was used to match phoshorylated serine, threonine and tyrosine in peptides found to be phoshorylated, according to prior experiments in literature for a given kinase based on PhosphoELM (http://phospho.elm.eu.org/) and on Phosphosite (http://www.phosphosite.org). [file 1471-2121-14-47-S3.docx]

| **PhosphoPeptide** | **Posição** | **Short_Description_Substrate** | **Kinase** |
| --- | --- | --- | --- |
| **ASRVPsSDEEV** | S1620 | 182 kDa tankyrase 1-binding protein | CK II\| Casein kinase II, alpha and alpha' chain |
| **SRVPSsDEEVV** | S1621 | 182 kDa tankyrase 1-binding protein | CK II\| Casein kinase II, alpha and alpha' chain |
| **KKEKKsLDSDE** | S57 | 28 kDa heat- and acid-stable phosphoprotein | CK II\| Casein kinase II, alpha and alpha' chain |
| **KKSLDsDESED** | S60 | 28 kDa heat- and acid-stable phosphoprotein | CK II\| Casein kinase II, alpha and alpha' chain |
| **LDSDEsEDEED** | S63 | 28 kDa heat- and acid-stable phosphoprotein | CK II\| Casein kinase II, alpha and alpha' chain |
| **EILPTtPISEQ** | T221 | 40S ribosomal protein S3 | CDK2\| Cell division protein kinase 2 |
| **RRRLSsLRAST** | S236 | 40S ribosomal protein S6 | RPS6KB1\| Ribosomal protein S6 kinase 1 |
| **RRRLSsLRAST** | S236 | 40S ribosomal protein S6 | PIM2\| Serine/threonine-protein kinase Pim-2 |
| **RRRLSsLRAST** | S236 | 40S ribosomal protein S6 | CLK1\| protein kinase CLK1 |
| **RRRLSsLRAST** | S236 | 40S ribosomal protein S6 | CLK2\| protein kinase CLK2 |
| **RRRLSsLRAST** | S236 | 40S ribosomal protein S6 | AKT1\| RAC-alpha serine/threonine-protein kinase |
| **RRRLSsLRAST** | S236 | 40S ribosomal protein S6 | AKT2\| RAC-beta serine/threonine-protein kinase |
| **RRRLSsLRAST** | S236 | 40S ribosomal protein S6 | Pim1\| Threonine-protein kinase Pim-1 |
| **RRRLSsLRAST** | S236 | 40S ribosomal protein S6 | DMPK\| Myotonin-protein kinase |
| **AEDSDsEPEPE** | S501 | 5`-3` exoribonuclease 2 | CK II\| Casein kinase II, alpha and alpha' chain |
| **AEDSDsEPEPE** | S501 | 5`-3` exoribonuclease 2 | CK II\| Casein kinase II, alpha and alpha' chain |
| **EAKEEsEESDE** | S304 | 60S acidic ribosomal protein P0 | CK II\| Casein kinase II, alpha and alpha' chain |
| **EESEEsDEDMG** | S307 | 60S acidic ribosomal protein P0 | CK II\| Casein kinase II, alpha and alpha' chain |
| **VEAKKEEsEESDDDM** | S101 | 60S acidic ribosomal protein P1 | CK II\| Casein kinase II, alpha and alpha' chain |
| **VEAKKEEsEESDDDM** | S101 | 60S acidic ribosomal protein P1 | CSNK1E\| Casein kinase I, epsilon isoform |
| **VEAKKEEsEESDDDM** | S101 | 60S acidic ribosomal protein P1 | CSNK1A1\| Casein kinase I, alpha isoform |
| **VEAKKEEsEESDDDM** | S101 | 60S acidic ribosomal protein P1 | CSNK1D\| Casein kinase I, delta isoform |
| **VEAKKEEsEESDDDM** | S101 | 60S acidic ribosomal protein P1 | CSNK1G2\| Casein kinase I, gamma 2 isoform |
| **KKEESEEsDDDMGFG** | S104 | 60S acidic ribosomal protein P1 | CK II\| Casein kinase II, alpha and alpha' chain |
| **KKEESEEsDDDMGFG** | S104 | 60S acidic ribosomal protein P1 | CSNK1E\| Casein kinase I, epsilon isoform |
| **KKEESEEsDDDMGFG** | S104 | 60S acidic ribosomal protein P1 | CSNK1A1\| Casein kinase I, alpha isoform |
| **KKEESEEsDDDMGFG** | S104 | 60S acidic ribosomal protein P1 | CSNK1D\| Casein kinase I, delta isoform |
| **KKEESEEsDDDMGFG** | S104 | 60S acidic ribosomal protein P1 | CSNK1G2\| Casein kinase I, gamma 2 isoform |
| **EKKEEsEESDD** | S102 | 60S acidic ribosomal protein P2 | CK II\| Casein kinase II, alpha and alpha' chain |
| **EESEEsDDDMG** | S105 | 60S acidic ribosomal protein P2 | CK II\| Casein kinase II, alpha and alpha' chain |
| **HSRQAsTDAGT** | S109 | 65 kDa Yes-associated protein (YAP65) | PIM2\| Serine/threonine-protein kinase Pim-2 |
| **HSRQAsTDAGT** | S109 | 65 kDa Yes-associated protein (YAP65) | CLK1\| protein kinase CLK1 |
| **HSRQAsTDAGT** | S109 | 65 kDa Yes-associated protein (YAP65) | CLK2\| protein kinase CLK2 |
| **HSRQAsTDAGT** | S109 | 65 kDa Yes-associated protein (YAP65) | CAMK2G\| CaMK-II gamma subunit |
| **KRKREtDDEGE** | T244 | Acidic leucine-rich nuclear phosphoprotein 32 | CK II\| Casein kinase II, alpha and alpha' chain |
| **EEGATsDGEKK** | S598 | A-kinase anchor protein 12 | CK II\| Casein kinase II, alpha and alpha' chain |
| **VLMIRtPEELD** | T634 | Alpha-1 catenin | CDK2\| Cell division protein kinase 2 |
| **VLMIRtPEELD** | T634 | Alpha-1 catenin | MAPK11\| Mitogen-activated protein kinase p38 beta |
| **VLMIRtPEELD** | T634 | Alpha-1 catenin | MAPK14\| Mitogen-activated protein kinase p38 alpha |
| **VLMIRtPEELD** | T634 | Alpha-1 catenin | MAPK13\| Mitogen-activated protein kinase p38 delta |
| **EELDDsDFETE** | S641 | Alpha-1 catenin | CK II\| Casein kinase II, alpha and alpha' chain |
| **PRKSSsISEEK** | S208 | Apoptotic chromatin condensation inducer in the nucleus | CK II\| Casein kinase II, alpha and alpha' chain |
| **LSVPTsDEEDE** | S109 | ATP-binding cassette sub-family F member 1 | CK II\| Casein kinase II, alpha and alpha' chain |
| **LSVPTsDEEDE** | S109 | ATP-binding cassette sub-family F member 1 | CK II\| Casein kinase II, alpha and alpha' chain |
| **PSRTAsFSESR** | S455 | ATP-citrate synthase | AKT1\| RAC-alpha serine/threonine-protein kinase |
| **PSRTAsFSESR** | S455 | ATP-citrate synthase | AKT2\| RAC-beta serine/threonine-protein kinase |
| **FCRSSsMADRS** | S20 | BAG-family molecular chaperone regulator-2 | CLK1\| protein kinase CLK1 |
| **FCRSSsMADRS** | S20 | BAG-family molecular chaperone regulator-2 | CLK2\| protein kinase CLK2 |
| **FCRSSsMADRS** | S20 | BAG-family molecular chaperone regulator-2 | PAK4\| Serine/threonine-protein kinase PAK 4 |
| **FCRSSsMADRS** | S20 | BAG-family molecular chaperone regulator-2 | PAK7\| Serine/threonine-protein kinase PAK 7 |
| **FCRSSsMADRS** | S20 | BAG-family molecular chaperone regulator-2 | TGFBR2\| TGF-beta receptor type II |
| **FCRSSsMADRS** | S20 | BAG-family molecular chaperone regulator-2 | ACTRIIA\| Activin receptor type II |
| **FCRSSsMADRS** | S20 | BAG-family molecular chaperone regulator-2 | ACVR2B\| Activin receptor type IIB |
| **SFGSVsPGGVK** | S962 | Band 4.1-like protein 3 | MAPK11\| Mitogen-activated protein kinase p38 beta |
| **SFGSVsPGGVK** | S962 | Band 4.1-like protein 3 | MAPK14\| Mitogen-activated protein kinase p38 alpha |
| **SFGSVsPGGVK** | S962 | Band 4.1-like protein 3 | MAPK13\| Mitogen-activated protein kinase p38 delta |
| **SFGSVsPGGVK** | S962 | Band 4.1-like protein 3 | CDK2\| Cell division protein kinase 2 |
| **EKRLStSPVRL** | T761 | Band 4.1-like protein 3 | DMPK\| Myotonin-protein kinase |
| **EKRLStSPVRL** | T761 | Band 4.1-like protein 3 | MOK\| MAPK/MAK/MRK overlapping kinase |
| **KRLSTsPVRLA** | S762 | Band 4.1-like protein 3 | CDK2\| Cell division protein kinase 2 |
| **KRLSTsPVRLA** | S762 | Band 4.1-like protein 3 | MAPK11\| Mitogen-activated protein kinase p38 beta |
| **KRLSTsPVRLA** | S762 | Band 4.1-like protein 3 | MAPK14\| Mitogen-activated protein kinase p38 alpha |
| **KRLSTsPVRLA** | S762 | Band 4.1-like protein 3 | MAPK13\| Mitogen-activated protein kinase p38 delta |
| **KRLSTsPVRLA** | S762 | Band 4.1-like protein 3 | MAPK8\| c-Jun N-terminal kinase 1 |
| **KRLSTsPVRLA** | S762 | Band 4.1-like protein 3 | MAPK10\| c-Jun N-terminal kinase 3 |
| **KRLSTsPVRLA** | S762 | Band 4.1-like protein 3 | MAPK9\| c-Jun N-terminal kinase 2 |
| **ALDYFsDKESG** | S385 | Bcl-2-associated transcription factor 1 | CK II\| Casein kinase II, alpha and alpha' chain |
| **QKFNDsEGDDT** | S397 | Bcl-2-associated transcription factor 1 | CK II\| Casein kinase II, alpha and alpha' chain |
| **KKETQsPEQVK** | S496 | Bcl-2-associated transcription factor 1 | CDK2\| Cell division protein kinase 2 |
| **KKETQsPEQVK** | S496 | Bcl-2-associated transcription factor 1 | MAPK11\| Mitogen-activated protein kinase p38 beta |
| **KKETQsPEQVK** | S496 | Bcl-2-associated transcription factor 1 | MAPK14\| Mitogen-activated protein kinase p38 alpha |
| **KKETQsPEQVK** | S496 | Bcl-2-associated transcription factor 1 | MAPK13\| Mitogen-activated protein kinase p38 delta |
| **KKETQsPEQVK** | S496 | Bcl-2-associated transcription factor 1 | MAPK8\| c-Jun N-terminal kinase 1 |
| **KKETQsPEQVK** | S496 | Bcl-2-associated transcription factor 1 | MAPK10\| c-Jun N-terminal kinase 3 |
| **KKETQsPEQVK** | S496 | Bcl-2-associated transcription factor 1 | MAPK9\| c-Jun N-terminal kinase 2 |
| **HSIQHsPERSG** | S268 | Bcl-2-associated transcription factor 1 | CDK2\| Cell division protein kinase 2 |
| **HSIQHsPERSG** | S268 | Bcl-2-associated transcription factor 1 | MAPK11\| Mitogen-activated protein kinase p38 beta |
| **HSIQHsPERSG** | S268 | Bcl-2-associated transcription factor 1 | MAPK14\| Mitogen-activated protein kinase p38 alpha |
| **HSIQHsPERSG** | S268 | Bcl-2-associated transcription factor 1 | MAPK13\| Mitogen-activated protein kinase p38 delta |
| **HSIQHsPERSG** | S268 | Bcl-2-associated transcription factor 1 | GSK3B\| Glycogen synthase kinase-3 beta |
| **HSIQHsPERSG** | S268 | Bcl-2-associated transcription factor 1 | GSK3A\| Glycogen synthase kinase-3 alpha |
| **STRQKsPEIHR** | S648 | Bcl-2-associated transcription factor 1 | CAMK2G\|CaMK-II gamma subunit |
| **STRQKsPEIHR** | S648 | Bcl-2-associated transcription factor 1 | CDK2\| Cell division protein kinase 2 |
| **STRQKsPEIHR** | S648 | Bcl-2-associated transcription factor 1 | MOK\| MAPK/MAK/MRK overlapping kinase |
| **EPQEEsPLKSK** | S177 | Bcl-2-associated transcription factor 1 | CDK2\| Cell division protein kinase 2 |
| **EDEGDsEPEAV** | S1468 | Bromodomain adjacent to zinc finger domain protein 1B | CK II\| Casein kinase II, alpha and alpha' chain |
| **TRRTRtFSATV** | T50 | Calcium-regulated heat stable protein 1 | RPS6KB1\| Ribosomal protein S6 kinase 1 |
| **VDKVTsPTKV-** | S789 | Caldesmon | CDK2\| Cell division protein kinase 2 |
| **KLEEKQKsDAEEDGG** | S554 | Calnexin precursor | CK II\| Casein kinase II, alpha and alpha' chain |
| **EDEILNRsPRNRKPR** | S583 | Calnexin | GSK3B\| Glycogen synthase kinase-3 beta |
| **EDEILNRsPRNRKPR** | S583 | Calnexin | MAPK8\| c-Jun N-terminal kinase 1 |
| **EDEILNRsPRNRKPR** | S583 | Calnexin | MAPK10\| c-Jun N-terminal kinase 3 |
| **EDEILNRsPRNRKPR** | S583 | Calnexin | CDK5\| Cell division protein kinase 5 |
| **EDEILNRsPRNRKPR** | S583 | Calnexin | MAPK11\| Mitogen-activated protein kinase p38 beta |
| **EDEILNRsPRNRKPR** | S583 | Calnexin | MAPK14\| Mitogen-activated protein kinase p38 alpha |
| **EDEILNRsPRNRKPR** | S583 | Calnexin | MAPK9\| c-Jun N-terminal kinase 2 |
| **EDEILNRsPRNRKPR** | S583 | Calnexin | CDC2\| Cell division control protein 2 homolog |
| **EDEILNRsPRNRKPR** | S583 | Calnexin | CDK2\| Cell division protein kinase 2 |
| **EDEILNRsPRNRKPR** | S583 | Calnexin | GSK3A\| Glycogen synthase kinase-3 alpha |
| **EDEILNRsPRNRKPR** | S583 | Calnexin | MAPK13\| Mitogen-activated protein kinase p38 delta |
| **EDEILNRsPRNRKPR** | S583 | Calnexin | MAPK12\| Mitogen-activated protein kinase 12 |
| **EDEILNRsPRNRKPR** | S583 | Calnexin | CDK3\| Cell division protein kinase 3 |
| **FTRRAsVCAEA** | S114 | cAMP-dependent protein kinase type II-beta reg. subunit | DMPK\| Myotonin-protein kinase |
| **FTRRAsVCAEA** | S114 | cAMP-dependent protein kinase type II-beta reg. subunit | PAK4\| Serine/threonine-protein kinase PAK 4 |
| **FTRRAsVCAEA** | S114 | cAMP-dependent protein kinase type II-beta reg. subunit | PKACA\| cAMP-dependent protein kinase, alpha-catalytic subunit |
| **FTRRAsVCAEA** | S114 | cAMP-dependent protein kinase type II-beta reg. subunit | PRKACB\| cAMP-dependent protein kinase, beta-catalytic subunit |
| **FTRRAsVCAEA** | S114 | cAMP-dependent protein kinase type II-beta reg. subunit | CLK1\| protein kinase CLK1 |
| **FTRRAsVCAEA** | S114 | cAMP-dependent protein kinase type II-beta reg. subunit | CLK2\| protein kinase CLK2 |
| **VCAEAyNPDEE** | Y120 | cAMP-dependent protein kinase type II-beta reg. subunit | MAP2K4\| mitogen-activated protein kinase kinase 4 |
| **VCAEAyNPDEE** | Y120 | cAMP-dependent protein kinase type II-beta reg. subunit | MAP2K3\| mitogen-activated protein kinase kinase 3 |
| **VCAEAyNPDEE** | Y120 | cAMP-dependent protein kinase type II-beta reg. subunit | MAP2K6\| mitogen-activated protein kinase kinase 6 |
| **VCAEAyNPDEE** | Y120 | cAMP-dependent protein kinase type II-beta reg. subunit | ITK\| Tyrosine-protein kinase ITK/TSK |
| **VCAEAyNPDEE** | Y120 | cAMP-dependent protein kinase type II-beta reg. subunit | BTK\| Tyrosine-protein kinase BTK |
| **VCAEAyNPDEE** | Y120 | cAMP-dependent protein kinase type II-beta reg. subunit | TEC\| Tyrosine-protein kinase Tec |
| **VRVGGsSVDLH** | S268 | Catenin delta-1 | CK II\| Casein kinase II, alpha and alpha' chain |
| **VRVGGsSVDLH** | S268 | Catenin delta-1 | PRKAA1\| 5`-AMP-activated protein kinase, catalytic alpha-1 chain |
| **VRVGGsSVDLH** | S268 | Catenin delta-1 | PRKAA2\| 5`-AMP-activated protein kinase, catalytic alpha-2 chain |
| **SFHDDsDEDLL** | S2484 | Insulin-like growth factor II receptor | CK II\| Casein kinase II, alpha and alpha' chain |
| **PSGPPsPNSPH** | S1690 | CDC42-binding protein kinase beta | CDK2\| Cell division protein kinase 2 |
| **PPSPNsPHRSQ** | S1693 | CDC42-binding protein kinase beta | CDK2\| Cell division protein kinase 2 |
| **SKATIsDEEIE** | S199 | Charged multivesicular body protein 2b | CK II\| Casein kinase II, alpha and alpha' chain |
| **DEYADsDEDQH** | S444 | Chromatin-specific transcription elongation factor | CK II\| Casein kinase II, alpha and alpha' chain |
| **GTKRKsLSDSE** | S93 | Chromobox protein homolog 3 | PAK4\| Serine/threonine-protein kinase PAK 4 |
| **GTKRKsLSDSE** | S93 | Chromobox protein homolog 3 | PAK7\| Serine/threonine-protein kinase PAK 7 |
| **GTKRKsLSDSE** | S93 | Chromobox protein homolog 3 | PIM2\| Serine/threonine-protein kinase Pim-2 |
| **KRKSLsDSESD** | S95 | Chromobox protein homolog 3 | CK II\| Casein kinase II, alpha and alpha' chain |
| **KKTKRtADSSS** | T8 | Chromobox protein homolog 5 | TGFBR2\| TGF-beta receptor type II |
| **KKTKRtADSSS** | T8 | Chromobox protein homolog 5 | ACVR2B\| Activin receptor type IIB |
| **KKTKRtADSSS** | T8 | Chromobox protein homolog 5 | PIM2\| Serine/threonine-protein kinase Pim-2 |
| **KRTADsSSSED** | S11 | Chromobox protein homolog 5 | CK II\| Casein kinase II, alpha and alpha' chain |
| **RTADSsSSEDE** | S12 | Chromobox protein homolog 5 | CK II\| Casein kinase II, alpha and alpha' chain |
| **TADSSsSEDEE** | S13 | Chromobox protein homolog 5 | CK II\| Casein kinase II, alpha and alpha' chain |
| **ADSSSsEDEEE** | S14 | Chromobox protein homolog 5 | CK II\| Casein kinase II, alpha and alpha' chain |
| **NDSSDsDDESH** | S437 | Cytosolic phospholipase A2 | CK II\| Casein kinase II, alpha and alpha' chain |
| **ASSAKtSPAKQ** | T521 | Dihydropyrimidinase-related protein 2 | MST2\| Serine/threonine-protein kinase 3 |
| **ASSAKtSPAKQ** | T521 | Dihydropyrimidinase-related protein 2 | AURKB\| Serine/threonine-protein kinase 12 |
| **ASSAKtSPAKQ** | T521 | Dihydropyrimidinase-related protein 2 | GSK3B\| Glycogen synthase kinase-3 beta |
| **ASSAKtSPAKQ** | T521 | Dihydropyrimidinase-related protein 2 | GSK3A\| Glycogen synthase kinase-3 alpha |
| **ASSAKtSPAKQ** | T521 | Dihydropyrimidinase-related protein 2 | STK24\| Serine/threonine-protein kinase 24 |
| **SSAKTsPAKQQ** | S522 | Dihydropyrimidinase-related protein 2 | CDK2\| Cell division protein kinase 2 |
| **GSARGsPTRPN** | S636 | Dihydropyrimidinase-related protein 3 | CDK2\| Cell division protein kinase 2 |
| **GSARGsPTRPN** | S636 | Dihydropyrimidinase-related protein 3 | MAPK11\| Mitogen-activated protein kinase p38 beta |
| **GSARGsPTRPN** | S636 | Dihydropyrimidinase-related protein 3 | MAPK14\| Mitogen-activated protein kinase p38 alpha |
| **GSARGsPTRPN** | S636 | Dihydropyrimidinase-related protein 3 | MAPK13\| Mitogen-activated protein kinase p38 delta |
| **GSARGsPTRPN** | S636 | Dihydropyrimidinase-related protein 3 | CDK5\| Cell division protein kinase 5 |
| **RSEDEsETEDE** | S672 | DNA replication licensing factor MCM3 | CK II\| Casein kinase II, alpha and alpha' chain |
| **VEAVNsDSDSE** | S1522 | DNA topoisomerase 2-beta | CK II\| Casein kinase II, alpha and alpha' chain |
| **AVNSDsDSEFG** | S1524 | DNA topoisomerase 2-beta | CK II\| Casein kinase II, alpha and alpha' chain |
| **PTRSPsDSSTA** | S339 | Drebrin | PIM2\| Serine/threonine-protein kinase Pim-2 |
| **LARLSsPVLHR** | S142 | Drebrin | CDK2\| Cell division protein kinase 2 |
| **LARLSsPVLHR** | S142 | Drebrin | MAPK11\| Mitogen-activated protein kinase p38 beta |
| **LARLSsPVLHR** | S142 | Drebrin | MAPK14\|Mitogen-activated protein kinase p38 alpha |
| **LARLSsPVLHR** | S142 | Drebrin | MAPK13\| Mitogen-activated protein kinase p38 delta |
| **LARLSsPVLHR** | S142 | Drebrin | MAPK8\| c-Jun N-terminal kinase 1 |
| **LARLSsPVLHR** | S142 | Drebrin | MAPK10\| c-Jun N-terminal kinase 3 |
| **LARLSsPVLHR** | S142 | Drebrin | MAPK9\| c-Jun N-terminal kinase 2 |
| **KPVTVsPTTPT** | S510 | Dynein light intermediate chain 1 | CDK2\| Cell division protein kinase 2 |
| **KPVTVsPTTPT** | S510 | Dynein light intermediate chain 1 | MAPK11\| Mitogen-activated protein kinase p38 beta |
| **KPVTVsPTTPT** | S510 | Dynein light intermediate chain 1 | MAPK14\| Mitogen-activated protein kinase p38 alpha |
| **KPVTVsPTTPT** | S510 | Dynein light intermediate chain 1 | MAPK13\| Mitogen-activated protein kinase p38 delta |
| **IDLFGsDDEEE** | S106 | Elongation factor 1-beta | CK II\| Casein kinase II, alpha and alpha' chain |
| **DDIDLFGsDNEEEDK** | S162 | Elongation factor 1-delta | CK II\| Casein kinase II, alpha and alpha' chain |
| **HRATAPQtQHVSPMR** | T495 | Elongation factor 1-delta | PRKCD\| Protein kinase C, delta type |
| **HRATAPQtQHVSPMR** | T495 | Elongation factor 1-delta | PKN1\| Protein kinase N1 (Protein kinase C-like 1) |
| **HRATAPQtQHVSPMR** | T495 | Elongation factor 1-delta | PRKCI\| Protein kinase C, iota type |
| **HRATAPQtQHVSPMR** | T495 | Elongation factor 1-delta | PRKCQ\| Protein kinase C, theta type |
| **HRATAPQtQHVSPMR** | T495 | Elongation factor 1-delta | PRKCZ\| Protein kinase C, zeta type |
| **HRATAPQtQHVSPMR** | T495 | Elongation factor 1-delta | PRKCA\| Protein kinase C, alpha type |
| **HRATAPQtQHVSPMR** | T495 | Elongation factor 1-delta | PRKCG\| Protein kinase C, gamma type |
| **HRATAPQtQHVSPMR** | T495 | Elongation factor 1-delta | PRKCB1\| Protein kinase C, beta type |
| **HRATAPQtQHVSPMR** | T495 | Elongation factor 1-delta | PRKCE\| Protein kinase C, epsilon type |
| **HRATAPQtQHVSPMR** | T495 | Elongation factor 1-delta | PKN2\| Protein kinase N2 (Protein kinase C-like 2) |
| **HRATAPQtQHVSPMR** | T495 | Elongation factor 1-delta | PRKCH\| Protein kinase C, eta type |
| **IDLFGsDNEEE** | S162 | Elongation factor 1-delta | CK II\| Casein kinase II, alpha and alpha' chain |
| **QTQHVsPMRQV** | S133 | Elongation factor 1-delta | CDK2\| Cell division protein kinase 2 |
| **AKKPAtPAEDD** | T147 | Elongation factor 1-delta | CDK2\| Cell division protein kinase 2 |
| **AKKPAtPAEDD** | T147 | Elongation factor 1-delta | CK II\| Casein kinase II, alpha and alpha' chain |
| **ATAPQtQHVSP** | T495 | Elongation factor 1-delta | ATM\| Serine-protein kinase ATM |
| **ATAPQtQHVSP** | T495 | Elongation factor 1-delta | ATR\| Serine-protein kinase ATR |
| **QPLLLsEDEED** | S39 | Eukaryotic translation initiation factor 3 subunit 8 | CK II\| Casein kinase II, alpha and alpha' chain |
| **PSESPsPPAAE** | S85 | Eukaryotic translation initiation factor 3 subunit 9 | MAPK11\| Mitogen-activated protein kinase p38 beta |
| **PSESPsPPAAE** | S85 | Eukaryotic translation initiation factor 3 subunit 9 | MAPK14\| Mitogen-activated protein kinase p38 alpha |
| **PSESPsPPAAE** | S85 | Eukaryotic translation initiation factor 3 subunit 9 | MAPK13\| Mitogen-activated protein kinase p38 delta |
| **PSESPsPPAAE** | S85 | Eukaryotic translation initiation factor 3 subunit 9 | MAPK10\| c-Jun N-terminal kinase 3 |
| **PSESPsPPAAE** | S85 | Eukaryotic translation initiation factor 3 subunit 9 | MAPK8\| c-Jun N-terminal kinase 1 |
| **PSESPsPPAAE** | S85 | Eukaryotic translation initiation factor 3 subunit 9 | MAPK9\| c-Jun N-terminal kinase 2 |
| **PSESPsPPAAE** | S85 | Eukaryotic translation initiation factor 3 subunit 9 | CDK2\| Cell division protein kinase 2 |
| **RERSRtGSESS** | T419 | Eukaryotic translation initiation factor 4B | PIM2\| Serine/threonine-protein kinase Pim-2 |
| **RERSRtGSESS** | T419 | Eukaryotic translation initiation factor 4B | CK II\| Casein kinase II, alpha and alpha' chain |
| **RERSRtGSESS** | T419 | Eukaryotic translation initiation factor 4B | RPS6KB1\| Ribosomal protein S6 kinase 1 |
| **RERSRtGSESS** | T419 | Eukaryotic translation initiation factor 4B | AKT1\| RAC-alpha serine/threonine-protein kinase |
| **RERSRtGSESS** | T419 | Eukaryotic translation initiation factor 4B | AKT2\| RAC-beta serine/threonine-protein kinase |
| **RERSRtGSESS** | T419 | Eukaryotic translation initiation factor 4B | Pim1\| Threonine-protein kinase Pim-1 |
| **RSRTGsESSQT** | S421 | Eukaryotic translation initiation factor 4B | PIM2\| Serine/threonine-protein kinase Pim-2 |
| **RSRTGsESSQT** | S421 | Eukaryotic translation initiation factor 4B | RPS6KB1\| Ribosomal protein S6 kinase 1 |
| **RSRTGsESSQT** | S421 | Eukaryotic translation initiation factor 4B | AKT1\| RAC-alpha serine/threonine-protein kinase |
| **RSRTGsESSQT** | S421 | Eukaryotic translation initiation factor 4B | AKT2\| RAC-beta serine/threonine-protein kinase |
| **RSRTGsESSQT** | S421 | Eukaryotic translation initiation factor 4B | CLK1\| protein kinase CLK1 |
| **RSRTGsESSQT** | S421 | Eukaryotic translation initiation factor 4B | CLK2\| protein kinase CLK2 |
| **PARSQsSDTEQ** | S496 | Eukaryotic translation initiation factor 4B | MOK\| MAPK/MAK/MRK overlapping kinase |
| **PARSQsSDTEQ** | S496 | Eukaryotic translation initiation factor 4B | TGFBR2\| TGF-beta receptor type II |
| **PARSQsSDTEQ** | S496 | Eukaryotic translation initiation factor 4B | ACTRIIA \| Activin receptor type II |
| **PARSQsSDTEQ** | S496 | Eukaryotic translation initiation factor 4B | CAMK2G\|CaMK-II gamma subunit |
| **ARSQSsDTEQQ** | S497 | Eukaryotic translation initiation factor 4B | CK II\| Casein kinase II, alpha and alpha' chain |
| **EMYSGsDDDDD** | S137 | Eukaryotic translation initiation factor 5B | CK II\| Casein kinase II, alpha and alpha' chain |
| **GPNIEsGNEDD** | S214 | Eukaryotic translation initiation factor 5B | CK II\| Casein kinase II, alpha and alpha' chain |
| **RRRAPsVANVG** | S2152 | Filamin A | PIM2\| Serine/threonine-protein kinase Pim-2 |
| **RRRAPsVANVG** | S2152 | Filamin A | CLK1\| protein kinase CLK1 |
| **RRRAPsVANVG** | S2152 | Filamin A | CLK2\| protein kinase CLK2 |
| **EDELEsGDQED** | S951 | General vesicular transport factor p115 | CK II\| Casein kinase II, alpha and alpha' chain |
| **EPNVSyICSRY** | Y279 | Glycogen synthase kinase-3 alpha | INSR\| Insulin receptor |
| **EPNVSyICSRY** | Y279 | Glycogen synthase kinase-3 alpha | IGF1R\| Insulin-like growth factor 1 receptor |
| **NRGGLsPANDT** | S47 | Glycylpeptide N-tetradecanoyltransferase 1 | CDK2\| Cell division protein kinase 2 |
| **NRGGLsPANDT** | S47 | Glycylpeptide N-tetradecanoyltransferase 1 | MAPK8\| c-Jun N-terminal kinase 1 |
| **NRGGLsPANDT** | S47 | Glycylpeptide N-tetradecanoyltransferase 1 | MAPK10\| c-Jun N-terminal kinase 3 |
| **NRGGLsPANDT** | S47 | Glycylpeptide N-tetradecanoyltransferase 1 | MAPK9\| c-Jun N-terminal kinase 2 |
| **NRGGLsPANDT** | S47 | Glycylpeptide N-tetradecanoyltransferase 1 | MAPK11\| Mitogen-activated protein kinase p38 beta |
| **NRGGLsPANDT** | S47 | Glycylpeptide N-tetradecanoyltransferase 1 | MAPK14\| Mitogen-activated protein kinase p38 alpha |
| **NRGGLsPANDT** | S47 | Glycylpeptide N-tetradecanoyltransferase 1 | MAPK13\| Mitogen-activated protein kinase p38 delta |
| **GAQSDsELPSY** | S1300 | BFA-resistant GEF 1 | CK II\| Casein kinase II, alpha and alpha' chain |
| **GAQSDsELPSY** | S1300 | BFA-resistant GEF 1 | TGFBR2\| TGF-beta receptor type II |
| **GAQSDsELPSY** | S1300 | BFA-resistant GEF 1 | ACTRIIA \| Activin receptor type II |
| **GAQSDsELPSY** | S1300 | BFA-resistant GEF 1 | ACVR2B\| Activin receptor type IIB |
| **GAQSDsELPSY** | S1300 | BFA-resistant GEF 1 | GSK3B\| Glycogen synthase kinase-3 beta |
| **GAQSDsELPSY** | S1300 | BFA-resistant GEF 1 | GSK3A\| Glycogen synthase kinase-3 alpha |
| **KAGLEsGAEPG** | S485 | H/ACA ribonucleoprotein complex subunit 4 | CK II\| Casein kinase II, alpha and alpha' chain |
| **RVHDRsEEEEE** | S99 | DDB1- and CUL4-associated factor 8 | CK II\| Casein kinase II, alpha and alpha' chain |
| **IEDVGsDEEEE** | S263 | Heat shock protein HSP 90-alpha | CK II\| Casein kinase II, alpha and alpha' chain |
| **IEDVGsDEEDD** | S255 | Heat shock protein HSP 90-beta | CK II\| Casein kinase II, alpha and alpha' chain |
| **DEEDDsGKDKK** | S261 | Heat shock protein HSP 90-beta | CK II\| Casein kinase II, alpha and alpha' chain |
| **GNAEGsSDEEG** | S132 | Hepatoma-derived growth factor (HDGF) | CK II\| Casein kinase II, alpha and alpha' chain |
| **NAEGSsDEEGK** | S133 | Hepatoma-derived growth factor (HDGF) | CK II\| Casein kinase II, alpha and alpha' chain |
| **DLLEDsPKRPK** | S165 | Hepatoma-derived growth factor (HDGF) | CDK2\| Cell division protein kinase 2 |
| **DLLEDsPKRPK** | S165 | Hepatoma-derived growth factor (HDGF) | CDK5\| Cell division protein kinase 5 |
| **MSKsESPKEPE** | S4 | Heterogeneous nuclear ribonucleoprotein A1 | CK II\| Casein kinase II, alpha and alpha' chain |
| **MSKSEsPKEPE** | S6 | Heterogeneous nuclear ribonucleoprotein A1 | CDK2\| Cell division protein kinase 2 |
| **MSKSEsPKEPE** | S6 | Heterogeneous nuclear ribonucleoprotein A1 | MAPK8\| c-Jun N-terminal kinase 1 |
| **MSKSEsPKEPE** | S6 | Heterogeneous nuclear ribonucleoprotein A1 | MAPK10\| c-Jun N-terminal kinase 3 |
| **MSKSEsPKEPE** | S6 | Heterogeneous nuclear ribonucleoprotein A1 | MAPK9\| c-Jun N-terminal kinase 2 |
| **SFGGRsSGSPY** | S355 | Heterogeneous nuclear ribonucleoprotein A3 | NEK2\| Serine/threonine-protein kinase Nek2 |
| **SFGGRsSGSPY** | S355 | Heterogeneous nuclear ribonucleoprotein A3 | TGFBR2\| TGF-beta receptor type II |
| **SFGGRsSGSPY** | S355 | Heterogeneous nuclear ribonucleoprotein A3 | ACTRIIA\| Activin receptor type II |
| **SFGGRsSGSPY** | S355 | Heterogeneous nuclear ribonucleoprotein A3 | ACVR2B\| Activin receptor type IIB |
| **PYGGGyGSGGG** | Y364 | Heterogeneous nuclear ribonucleoprotein A3 | IGF1R\| Insulin-like growth factor 1 receptor |
| **PYGGGyGSGGG** | Y364 | Heterogeneous nuclear ribonucleoprotein A3 | INSR\| Insulin receptor |
| **HTGPNsPDTAN** | S104 | Heterogeneous nuclear ribonucleoprotein H | CDK2\| Cell division protein kinase 2 |
| **DYDDMsPRRGP** | S284 | Heterogeneous nuclear ribonucleoprotein K | CDK2\| Cell division protein kinase 2 |
| **DYDDMsPRRGP** | S284 | Heterogeneous nuclear ribonucleoprotein K | MAPK11\| Mitogen-activated protein kinase p38 beta |
| **DYDDMsPRRGP** | S284 | Heterogeneous nuclear ribonucleoprotein K | MAPK14\| Mitogen-activated protein kinase p38 alpha |
| **DYDDMsPRRGP** | S284 | Heterogeneous nuclear ribonucleoprotein K | MAPK13\| Mitogen-activated protein kinase p38 delta |
| **MKNDKsEEEQS** | S233 | Heterogeneous nuclear ribonucleoproteins C1/C2 | CK II\| Casein kinase II, alpha and alpha' chain |
| **YSRAKsPQPPV** | S252 | Heterogenous nuclear ribonucleoprotein U | CDK2\| Cell division protein kinase 2 |
| **EEEGIsQESSE** | S99 | High mobility group protein HMG-I/HMG-Y | ATM\| Serine-protein kinase ATM |
| **EEEGIsQESSE** | S99 | High mobility group protein HMG-I/HMG-Y | \| Serine-protein kinase ATR |
| **EEEGIsQESSE** | S99 | High mobility group protein HMG-I/HMG-Y | CK II\| Casein kinase II, alpha and alpha' chain |
| **GISQEsSEEEQ** | S102 | High mobility group protein HMG-I/HMG-Y | CK II\| Casein kinase II, alpha and alpha' chain |
| **ISQESsEEEQ-** | S103 | High mobility group protein HMG-I/HMG-Y | CK II\| Casein kinase II, alpha and alpha' chain |
| **AIPEEsGDEDE** | S393 | Histone deacetylase 1 | CK II\| Casein kinase II, alpha and alpha' chain |
| **AVHEDsGDEDG** | S394 | Histone deacetylase 2 | CK II\| Casein kinase II, alpha and alpha' chain |
| **APVEKsPAKKK** | S18 | Histone H1.5 | CDK2\| Cell division protein kinase 2 |
| **ESLEDsDVDAD** | S60 | Importin alpha-3 subunit | CK II\| Casein kinase II, alpha and alpha' chain |
| **GSPSPAGtPPQPKRP** | T490 | Junctophilin-2 | MAPK8\| c-Jun N-terminal kinase 1 |
| **GSPSPAGtPPQPKRP** | T490 | Junctophilin-2 | CDC2\| Cell division control protein 2 homolog |
| **GSPSPAGtPPQPKRP** | T490 | Junctophilin-2 | GSK3B\| Glycogen synthase kinase-3 beta |
| **GSPSPAGtPPQPKRP** | T490 | Junctophilin-2 | MAPK10\| c-Jun N-terminal kinase 3 |
| **GSPSPAGtPPQPKRP** | T490 | Junctophilin-2 | CDK2\| Cell division protein kinase 2 |
| **GSPSPAGtPPQPKRP** | T490 | Junctophilin-2 | GSK3A\| Glycogen synthase kinase-3 alpha |
| **GSPSPAGtPPQPKRP** | T490 | Junctophilin-2 | MAPK11\| Mitogen-activated protein kinase p38 beta |
| **GSPSPAGtPPQPKRP** | T490 | Junctophilin-2 | MAPK14\| Mitogen-activated protein kinase p38 alpha |
| **GSPSPAGtPPQPKRP** | T490 | Junctophilin-2 | MAPK9\| c-Jun N-terminal kinase 2 |
| **GSPSPAGtPPQPKRP** | T490 | Junctophilin-2 | CDK5\| Cell division protein kinase 5 (EC 2.7.1.37) |
| **GSPSPAGtPPQPKRP** | T490 | Junctophilin-2 | MAPK13\| Mitogen-activated protein kinase p38 delta |
| **GSPSPAGtPPQPKRP** | T490 | Junctophilin-2 | MAPK12\| Mitogen-activated protein kinase 12 |
| **GSPSPAGtPPQPKRP** | T490 | Junctophilin-2 | CDK3\| Cell division protein kinase 3 |
| **GSPSPAGtPPQPKRP** | T490 | Junctophilin-2 | PFTK1\| Serine/threonine-protein kinase PFTAIRE-1 |
| **GSPSPAGtPPQPKRP** | T490 | Junctophilin-2 | PCTK1\| Serine/threonine-protein kinase PCTAIRE-1 |
| **VEDERsDREET** | S191 | LAG1 longevity assurance homolog 2 | CK II\| Casein kinase II, alpha and alpha' chain |
| **SDREEtESSEG** | T196 | LAG1 longevity assurance homolog 2 | CK II\| Casein kinase II, alpha and alpha' chain |
| **REETEsSEGEE** | S198 | LAG1 longevity assurance homolog 2 | CK II\| Casein kinase II, alpha and alpha' chain |
| **EETESsEGEEA** | S199 | LAG1 longevity assurance homolog 2 | CK II\| Casein kinase II, alpha and alpha' chain |
| **VEDERsDREET** | S341 | LAG1 longevity assurance homolog 2 | CK II\| Casein kinase II, alpha and alpha' chain |
| **SDREEtESSEG** | T346 | LAG1 longevity assurance homolog 2 | CK II\| Casein kinase II, alpha and alpha' chain |
| **REETEsSEGEE** | S348 | LAG1 longevity assurance homolog 2 | CK II\| Casein kinase II, alpha and alpha' chain |
| **EETESsEGEEA** | S349 | LAG1 longevity assurance homolog 2 | CK II\| Casein kinase II, alpha and alpha' chain |
| **RRATRsGAQAS** | S12 | Lamin A/C | PIM2\| Serine/threonine-protein kinase Pim-2 |
| **RRATRsGAQAS** | S12 | Lamin A/C | Pim1\| Threonine-protein kinase Pim-1 |
| **SSTPLsPTRIT** | S22 | Lamin A/C | CDK2\| Cell division protein kinase 2 |
| **ERLRLsPSPTS** | S390 | Lamin A/C | CDK2\| Cell division protein kinase 2 |
| **ERLRLsPSPTS** | S390 | Lamin A/C | DMPK\| Myotonin-protein kinase |
| **ERLRLsPSPTS** | S390 | Lamin A/C | MAPK8\| c-Jun N-terminal kinase 1 |
| **ERLRLsPSPTS** | S390 | Lamin A/C | MAPK10\| c-Jun N-terminal kinase 3 |
| **ERLRLsPSPTS** | S390 | Lamin A/C | MAPK9\| c-Jun N-terminal kinase 2 |
| **ERLRLsPSPTS** | S390 | Lamin A/C | MAPK11\| Mitogen-activated protein kinase p38 beta |
| **ERLRLsPSPTS** | S390 | Lamin A/C | MAPK14\| Mitogen-activated protein kinase p38 alpha |
| **ERLRLsPSPTS** | S390 | Lamin A/C | MAPK13\| Mitogen-activated protein kinase p38 delta |
| **LRLSPsPTSQR** | S392 | Lamin A/C | MAPK11\| Mitogen-activated protein kinase p38 beta |
| **LRLSPsPTSQR** | S392 | Lamin A/C | MAPK14\| Mitogen-activated protein kinase p38 alpha |
| **LRLSPsPTSQR** | S392 | Lamin A/C | MAPK13\| Mitogen-activated protein kinase p38 delta |
| **ASSHSsQTQGG** | S407 | Lamin A/C | ATM\| Serine-protein kinase ATM |
| **ASSHSsQTQGG** | S407 | Lamin A/C | \| Serine-protein kinase ATR |
| **ARRSAsASHQA** | S99 | Lamin B receptor | PIM2\| Serine/threonine-protein kinase Pim-2 |
| **ARRSAsASHQA** | S99 | Lamin B receptor | MOK\| MAPK/MAK/MRK overlapping kinase |
| **TESRSsTPLPT** | S159 | Lamina-associated polypeptide 2 isoform alpha | PAK4\| Serine/threonine-protein kinase PAK 4 |
| **TESRSsTPLPT** | S159 | Lamina-associated polypeptide 2 isoform alpha | PAK7\| Serine/threonine-protein kinase PAK 7 |
| **TESRSsTPLPT** | S159 | Lamina-associated polypeptide 2 isoform alpha | AURKB\| Serine/threonine-protein kinase 12 |
| **TESRSsTPLPT** | S159 | Lamina-associated polypeptide 2 isoform alpha | PAK2\| Serine/threonine-protein kinase PAK 2 |
| **TESRSsTPLPT** | S159 | Lamina-associated polypeptide 2 isoform alpha | \| Serine/threonine-protein kinase PAK 1 |
| **TESRSsTPLPT** | S159 | Lamina-associated polypeptide 2 isoform alpha | PAK3\| Serine/threonine-protein kinase PAK 3 |
| **ESRSStPLPTI** | T160 | Lamina-associated polypeptide 2 isoform alpha | CDK2\| Cell division protein kinase 2 |
| **TESRSsTPLPT** | S158 | Lamina-associated polypeptide 2 isoform alpha | PAK4\| Serine/threonine-protein kinase PAK 4 |
| **TESRSsTPLPT** | S158 | Lamina-associated polypeptide 2 isoform alpha | PAK7\| Serine/threonine-protein kinase PAK 7 |
| **TESRSsTPLPT** | S158 | Lamina-associated polypeptide 2 isoform alpha | AURKB\| Serine/threonine-protein kinase 12 |
| **TESRSsTPLPT** | S158 | Lamina-associated polypeptide 2 isoform alpha | PAK2\| Serine/threonine-protein kinase PAK 2 |
| **TESRSsTPLPT** | S158 | Lamina-associated polypeptide 2 isoform alpha | \| Serine/threonine-protein kinase PAK 1 |
| **TESRSsTPLPT** | S158 | Lamina-associated polypeptide 2 isoform alpha | PAK3\| Serine/threonine-protein kinase PAK 3 |
| **GPPDFsSDEER** | S66 | Lamina-associated polypeptide 2 isoform alpha | CK II\| Casein kinase II, alpha and alpha' chain |
| **PPDFSsDEERE** | S67 | Lamina-associated polypeptide 2 isoform alpha | CK II\| Casein kinase II, alpha and alpha' chain |
| **KSRKRsYSPDG** | S596 | Matrin-3 | PIM2\| Serine/threonine-protein kinase Pim-2 |
| **KSRKRsYSPDG** | S596 | Matrin-3 | RPS6KB1\| Ribosomal protein S6 kinase 1 |
| **RKRSYsPDGKE** | S598 | Matrin-3 | PIM2\| Serine/threonine-protein kinase Pim-2 |
| **RKRSYsPDGKE** | S598 | Matrin-3 | RPS6KB1\| Ribosomal protein S6 kinase 1 |
| **RKRSYsPDGKE** | S598 | Matrin-3 | CAMK2G\| CaMK-II gamma subunit) |
| **RKRSYsPDGKE** | S598 | Matrin-3 | MOK\| MAPK/MAK/MRK overlapping kinase |
| **PDGKEsPSDKK** | S604 | Matrin-3 | CDK2\| Cell division protein kinase 2 |
| **EPTVYsDEEEP** | S181 | Membrane associated progesterone receptor component 1 | CK II\| Casein kinase II, alpha and alpha' chain |
| **EPSEYtDEEDT** | T211 | Membrane associated progesterone receptor component 2 | CK II\| Casein kinase II, alpha and alpha' chain |
| **EVVGEsDSEVE** | S116 | Microfibrillar-associated protein 1 | CK II\| Casein kinase II, alpha and alpha' chain |
| **VGESDsEVEGD** | S118 | Microfibrillar-associated protein 1 | CK II\| Casein kinase II, alpha and alpha' chain |
| **IEKVLsPLRSP** | S1396 | Microtubule-associated protein 1B | CDK2\| Cell division protein kinase 2 |
| **LSPLRsPPLIG** | S1400 | Microtubule-associated protein 1B | CDK2\| Cell division protein kinase 2 |
| **PEGRAsPAPGS** | S91 | Mitochondrial precursor proteins import receptor | MAPK11\| Mitogen-activated protein kinase p38 beta |
| **PEGRAsPAPGS** | S91 | Mitochondrial precursor proteins import receptor | MAPK14\| Mitogen-activated protein kinase p38 alpha |
| **PEGRAsPAPGS** | S91 | Mitochondrial precursor proteins import receptor | MAPK13\| Mitogen-activated protein kinase p38 delta |
| **PEGRAsPAPGS** | S91 | Mitochondrial precursor proteins import receptor | MAPK10\| c-Jun N-terminal kinase 3 |
| **PEGRAsPAPGS** | S91 | Mitochondrial precursor proteins import receptor | MAPK8\| c-Jun N-terminal kinase 1 |
| **PEGRAsPAPGS** | S91 | Mitochondrial precursor proteins import receptor | MAPK9\| c-Jun N-terminal kinase 2 |
| **DKSKAsLEKAG** | S213 | Monocarboxylate transporter | CAMK2G\| CaMK-II gamma subunit |
| **PSATQsPISKK** | S1163 | Myb-binding protein 1A | CDK2\| Cell division protein kinase 2 |
| **PSATQsPISKK** | S1163 | Myb-binding protein 1A | MAPK11\| Mitogen-activated protein kinase p38 beta |
| **PSATQsPISKK** | S1163 | Myb-binding protein 1A | MAPK14\| Mitogen-activated protein kinase p38 alpha |
| **PSATQsPISKK** | S1163 | Myb-binding protein 1A | MAPK13\| Mitogen-activated protein kinase p38 delta |
| **ASLELsDDDTE** | S1956 | Myosin-10 | CK II\| Casein kinase II, alpha and alpha' chain |
| **GAGDGsDEEVD** | S1943 | Myosin-9 | CK II\| Casein kinase II, alpha and alpha' chain |
| **DGATPsPSNET** | S145 | Myristoylated alanine-rich C-kinase substrate | CDK2\| Cell division protein kinase 2 |
| **DGATPsPSNET** | S145 | Myristoylated alanine-rich C-kinase substrate | MAPK8\| c-Jun N-terminal kinase 1 |
| **DGATPsPSNET** | S145 | Myristoylated alanine-rich C-kinase substrate | MAPK10\| c-Jun N-terminal kinase 3 |
| **DGATPsPSNET** | S145 | Myristoylated alanine-rich C-kinase substrate | MAPK9\| c-Jun N-terminal kinase 2 |
| **GPRLGsPSGKT** | S559 | Protein AHNAK | CDK2\| Cell division protein kinase 2 |
| **GPRLGsPSGKT** | S559 | Protein AHNAK | MAPK11\| Mitogen-activated protein kinase p38 beta |
| **GPRLGsPSGKT** | S559 | Protein AHNAK | MAPK14\| Mitogen-activated protein kinase p38 alpha |
| **GPRLGsPSGKT** | S559 | Protein AHNAK | MAPK13\| Mitogen-activated protein kinase p38 delta |
| **GPRLGsPSGKT** | S559 | Protein AHNAK | MOK\| MAPK/MAK/MRK overlapping kinase |
| **GPRLGsPSGKT** | S559 | Protein AHNAK | MAPK8\| c-Jun N-terminal kinase 1 |
| **GPRLGsPSGKT** | S559 | Protein AHNAK | MAPK10\| c-Jun N-terminal kinase 3 |
| **GPRLGsPSGKT** | S559 | Protein AHNAK | MAPK9\| c-Jun N-terminal kinase 2 |
| **GGVTGsPEASI** | S5731 | Protein AHNAK | CDK2\| Cell division protein kinase 2 |
| **GGVTGsPEASI** | S5731 | Protein AHNAK | MAPK11\| Mitogen-activated protein kinase p38 beta |
| **GGVTGsPEASI** | S5731 | Protein AHNAK | MAPK14\| Mitogen-activated protein kinase p38 alpha |
| **GGVTGsPEASI** | S5731 | Protein AHNAK | MAPK13\| Mitogen-activated protein kinase p38 delta |
| **GGVTGsPEASI** | S5731 | Protein AHNAK | MAPK8\| c-Jun N-terminal kinase 1 |
| **GGVTGsPEASI** | S5731 | Protein AHNAK | MAPK10\| c-Jun N-terminal kinase 3 |
| **GGVTGsPEASI** | S5731 | Protein AHNAK | MAPK9\| c-Jun N-terminal kinase 2 |
| **KPRHRsNSFSD** | S5780 | Protein AHNAK | PIM2\| Serine/threonine-protein kinase Pim-2 |
| **KPRHRsNSFSD** | S5780 | Protein AHNAK | RPS6KB1\| Ribosomal protein S6 kinase 1 |
| **KEMLAsDDEED** | S80 | Nexilin | CK II\| Casein kinase II, alpha and alpha' chain |
| **MYKTIsQEFLT** | S365 | Nexilin | ATM\| Serine-protein kinase ATM |
| **MYKTIsQEFLT** | S365 | Nexilin | \| Serine-protein kinase ATR |
| **PKRKVsSAEGA** | S7 | Nonhistone chromosomal protein HMG-14 | PIM2\| Serine/threonine-protein kinase Pim-2 |
| **SKLPRtQPDGT** | T1744 | Nuclear mitotic apparatus protein 1 | ATM\| Serine-protein kinase ATM |
| **SKLPRtQPDGT** | T1744 | Nuclear mitotic apparatus protein 1 | \| Serine-protein kinase ATR |
| **PGEPAsPISQR** | S1757 | Nuclear mitotic apparatus protein 1 | CDK2\| Cell division protein kinase 2 |
| **SQFQEsDDADE** | S19 | Nuclear ubiquitous casein and cyclin-dependent kinases substrate | CK II\| Casein kinase II, alpha and alpha' chain |
| **NSQEDsEDSED** | S58 | Nuclear ubiquitous casein and cyclin-dependent kinases substrate | CK II\| Casein kinase II, alpha and alpha' chain |
| **ATVTPsPVKGK** | S181 | Nuclear ubiquitous casein and cyclin-dependent kinases substrate | CDK2\| Cell division protein kinase 2 |
| **ATVTPsPVKGK** | S181 | Nuclear ubiquitous casein and cyclin-dependent kinases substrate | MAPK8\| c-Jun N-terminal kinase 1 |
| **ATVTPsPVKGK** | S181 | Nuclear ubiquitous casein and cyclin-dependent kinases substrate | MAPK10\| c-Jun N-terminal kinase 3 |
| **ATVTPsPVKGK** | S181 | Nuclear ubiquitous casein and cyclin-dependent kinases substrate | MAPK9\| c-Jun N-terminal kinase 2 |
| **PAENSsAPEAE** | S314 | Nuclease sensitive element binding protein 1 | CK II\| Casein kinase II, alpha and alpha' chain |
| **KAAKNsEEEEE** | S563 | Nucleolar phosphoprotein p130 | CK II\| Casein kinase II, alpha and alpha' chain |
| **EELMSsDLEET** | S520 | Nucleolar protein Nop56 | CK II\| Casein kinase II, alpha and alpha' chain |
| **EVEEDsEDEEM** | S28 | Nucleolin | CK II\| Casein kinase II, alpha and alpha' chain |
| **EDEEMsEDEED** | S34 | Nucleolin | CK II\| Casein kinase II, alpha and alpha' chain |
| **DEEDDsSGEEV** | S41 | Nucleolin | CK II\| Casein kinase II, alpha and alpha' chain |
| **AKKEDsDEEED** | S145 | Nucleolin | CK II\| Casein kinase II, alpha and alpha' chain |
| **AAAPAsEDEDD** | S184 | Nucleolin | CK II\| Casein kinase II, alpha and alpha' chain |
| **EEDAEsEDEEE** | S125 | Nucleophosmin | CK II\| Casein kinase II, alpha and alpha' chain |
| **KTGVTSTsDSEEEGD** | S273 | PC4 and SFRS1 interacting protein | CSNK1E\| Casein kinase I, epsilon isoform |
| **KTGVTSTsDSEEEGD** | S273 | PC4 and SFRS1 interacting protein | CSNK1A1\| Casein kinase I, alpha isoform |
| **KTGVTSTsDSEEEGD** | S273 | PC4 and SFRS1 interacting protein | CK II\| Casein kinase II, alpha and alpha' chain |
| **KTGVTSTsDSEEEGD** | S273 | PC4 and SFRS1 interacting protein | CSNK1D\| Casein kinase I, delta isoform |
| **KTGVTSTsDSEEEGD** | S273 | PC4 and SFRS1 interacting protein | CSNK1G2\| Casein kinase I, gamma 2 isoform |
| **GVTSTSDsEEEGDDQ** | S275 | PC4 and SFRS1 interacting protein | CSNK1E\| Casein kinase I, epsilon isoform |
| **GVTSTSDsEEEGDDQ** | S275 | PC4 and SFRS1 interacting protein | CSNK1A1\| Casein kinase I, alpha isoform |
| **GVTSTSDsEEEGDDQ** | S275 | PC4 and SFRS1 interacting protein | CK II\| Casein kinase II, alpha and alpha' chain |
| **GVTSTSDsEEEGDDQ** | S275 | PC4 and SFRS1 interacting protein | CSNK1D\| Casein kinase I, delta isoform |
| **GVTSTSDsEEEGDDQ** | S275 | PC4 and SFRS1 interacting protein | CSNK1G2\| Casein kinase I, gamma 2 isoform |
| **TSRDTsPSSGS** | S205 | Periphilin 1 | CDK2\| Cell division protein kinase 2 |
| **TSRDTsPSSGS** | S205 | Periphilin 1 | CLK1\| protein kinase CLK1 |
| **TSRDTsPSSGS** | S205 | Periphilin 1 | CLK2\| protein kinase CLK2 |
| **TSRDTsPSSGS** | S205 | Periphilin 1 | MAPK11\| Mitogen-activated protein kinase p38 beta |
| **TSRDTsPSSGS** | S205 | Periphilin 1 | MAPK14\| Mitogen-activated protein kinase p38 alpha |
| **TSRDTsPSSGS** | S205 | Periphilin 1 | MAPK13\| Mitogen-activated protein kinase p38 delta |
| **TSRDTsPSSGS** | S205 | Periphilin 1 | MAPK8\| c-Jun N-terminal kinase 1 |
| **TSRDTsPSSGS** | S205 | Periphilin 1 | MAPK10\| c-Jun N-terminal kinase 3 |
| **TSRDTsPSSGS** | S205 | Periphilin 1 | MAPK9\| c-Jun N-terminal kinase 2 |
| **GFRSRsSSVGS** | S4384 | Plectin 1 | PAK4\| Serine/threonine-protein kinase PAK 4 |
| **GFRSRsSSVGS** | S4384 | Plectin 1 | PAK7\| Serine/threonine-protein kinase PAK 7 |
| **GFRSRsSSVGS** | S4384 | Plectin 1 | NEK2\| Serine/threonine-protein kinase Nek2 |
| **GFRSRsSSVGS** | S4384 | Plectin 1 | DMPK\| Myotonin-protein kinase |
| **GFRSRsSSVGS** | S4384 | Plectin 1 | RPS6KB1\| Ribosomal protein S6 kinase 1 |
| **RSRSSsVGSSS** | S4386 | Plectin 1 | PIM2\| Serine/threonine-protein kinase Pim-2 |
| **RSRSSsVGSSS** | S4386 | Plectin 1 | AKT1\| RAC-alpha serine/threonine-protein kinase |
| **RSRSSsVGSSS** | S4386 | Plectin 1 | AKT2\| RAC-beta serine/threonine-protein kinase |
| **RSRSSsVGSSS** | S4386 | Plectin 1 | PAK4\| Serine/threonine-protein kinase PAK 4 |
| **RSRSSsVGSSS** | S4386 | Plectin 1 | PAK7\| Serine/threonine-protein kinase PAK 7 |
| **RSRSSsVGSSS** | S4386 | Plectin 1 | RPS6KB1\| Ribosomal protein S6 kinase 1 |
| **RSRSSsVGSSS** | S4386 | Plectin 1 | TGFBR2\| TGF-beta receptor type II |
| **RSRSSsVGSSS** | S4386 | Plectin 1 | ACTRIIA\| Activin receptor type II |
| **RSRSSsVGSSS** | S4386 | Plectin 1 | ACVR2B\| Activin receptor type IIB |
| **RSRSSsVGSSS** | S4386 | Plectin 1 | CLK1\| protein kinase CLK1 |
| **RSRSSsVGSSS** | S4386 | Plectin 1 | CLK2\| protein kinase CLK2 |
| **RKVMDsDEDDD** | S119 | Programmed cell death protein 5 | CK II\| Casein kinase II, alpha and alpha' chain |
| **KYAKEsLKEED** | S243 | Proteasome subunit alpha type 3 | CK II\| Casein kinase II, alpha and alpha' chain |
| **KEEDEsDDDNM** | S250 | Proteasome subunit alpha type 3 | CK II\| Casein kinase II, alpha and alpha' chain |
| **RKDDDsDDESQ** | S458 | Protein KIAA0776 | CK II\| Casein kinase II, alpha and alpha' chain |
| **GGGEEsEGEEV** | S304 | purine-rich element binding protein B | CK II\| Casein kinase II, alpha and alpha' chain |
| **RYHGHsMSDPG** | S293 | Pyruvate dehydrogenase E1 component alpha subunit | RPS6KB1\| Ribosomal protein S6 kinase 1 |
| **HGHSMsDPGVS** | S295 | Pyruvate dehydrogenase E1 component alpha subunit | SGK\| Serine/threonine-protein kinase Sgk1 |
| **TQFPPsQSEER** | S126 | Ran-binding protein 3 | ATM\| Serine-protein kinase ATM |
| **TQFPPsQSEER** | S126 | Ran-binding protein 3 | \| Serine-protein kinase ATR |
| **AQKSSsPAPAD** | S232 | Ras-GTPase-activating protein binding protein 1 | CDK2\| Cell division protein kinase 2 |
| **AQKSSsPAPAD** | S232 | Ras-GTPase-activating protein binding protein 1 | MAPK11\| Mitogen-activated protein kinase p38 beta |
| **AQKSSsPAPAD** | S232 | Ras-GTPase-activating protein binding protein 1 | MAPK14\| Mitogen-activated protein kinase p38 alpha |
| **AQKSSsPAPAD** | S232 | Ras-GTPase-activating protein binding protein 1 | MAPK13\| Mitogen-activated protein kinase p38 delta |
| **AQKSSsPAPAD** | S232 | Ras-GTPase-activating protein binding protein 1 | MAPK8\| c-Jun N-terminal kinase 1 |
| **AQKSSsPAPAD** | S232 | Ras-GTPase-activating protein binding protein 1 | MAPK10\| c-Jun N-terminal kinase 3 |
| **AQKSSsPAPAD** | S232 | Ras-GTPase-activating protein binding protein 1 | MAPK9\| c-Jun N-terminal kinase 2 |
| **QECPPsPEPTR** | S186 | Ras-related protein R-Ras2 | CDK2\| Cell division protein kinase 2 |
| **PERQPsWDPSP** | S107 | Reticulon-4 | PIM2\| Serine/threonine-protein kinase Pim-2 |
| **PERQPsWDPSP** | S107 | Reticulon-4 | CAMK2G\| CaMK-II gamma subunit |
| **DSKSSsPELVT** | S51 | Rho-GTPase-activating protein 1 | CDK2\| Cell division protein kinase 2 |
| **DSKSSsPELVT** | S51 | Rho-GTPase-activating protein 1 | MAPK11\| Mitogen-activated protein kinase p38 beta |
| **DSKSSsPELVT** | S51 | Rho-GTPase-activating protein 1 | MAPK14\| Mitogen-activated protein kinase p38 alpha |
| **DSKSSsPELVT** | S51 | Rho-GTPase-activating protein 1 | MAPK13\| Mitogen-activated protein kinase p38 delta |
| **DSKSSsPELVT** | S51 | Rho-GTPase-activating protein 1 | MAPK8\| c-Jun N-terminal kinase 1 |
| **DSKSSsPELVT** | S51 | Rho-GTPase-activating protein 1 | MAPK10\| c-Jun N-terminal kinase 3 |
| **DSKSSsPELVT** | S51 | Rho-GTPase-activating protein 1 | MAPK9\| c-Jun N-terminal kinase 2 |
| **KKAEGsPNQGK** | S583 | Ribosome-binding protein 1 | CDK2\| Cell division protein kinase 2 |
| **KKAEGsPNQGK** | S583 | Ribosome-binding protein 1 | MAPK11\| Mitogen-activated protein kinase p38 beta |
| **KKAEGsPNQGK** | S583 | Ribosome-binding protein 1 | MAPK14\| Mitogen-activated protein kinase p38 alpha |
| **KKAEGsPNQGK** | S583 | Ribosome-binding protein 1 | MAPK13\| Mitogen-activated protein kinase p38 delta |
| **YHLPDAEsDEDEDFK** | S218 | Septin-2 (NEDD5 protein homolog) | CK II\| Casein kinase II, alpha and alpha' chain |
| **APQTSsSPPPV** | S695 | serine/arginine repetitive matrix 1 | STK6\| Serine/threonine-protein kinase 6 |
| **APQTSsSPPPV** | S695 | serine/arginine repetitive matrix 1 | NEK2\| Serine/threonine-protein kinase Nek2 |
| **IGKARsPTDDK** | S257 | Serine/threonine-protein kinase PRP4 homolog | CDK2\| Cell division protein kinase 2 |
| **KDRKKsPIINE** | S277 | Serine/threonine-protein kinase PRP4 homolog | CDK2\| Cell division protein kinase 2 |
| **KDRKKsPIINE** | S277 | Serine/threonine-protein kinase PRP4 homolog | CAMK2G\| CaMK-II gamma subunit |
| **KDRKKsPIINE** | S277 | Serine/threonine-protein kinase PRP4 homolog | MAPK10\| c-Jun N-terminal kinase 3 |
| **KDRKKsPIINE** | S277 | Serine/threonine-protein kinase PRP4 homolog | MAPK8\| c-Jun N-terminal kinase 1 |
| **KDRKKsPIINE** | S277 | Serine/threonine-protein kinase PRP4 homolog | MAPK9\| c-Jun N-terminal kinase 2 |
| **SGSPHsPHQLS** | S2032 | Serine/threonine-protein kinase WNK1 | CDK2\| Cell division protein kinase 2 |
| **RSQIRsRTPSA** | S301 | SGTA protein | GSK3B\| Glycogen synthase kinase-3 beta |
| **RSQIRsRTPSA** | S301 | SGTA protein | GSK3A\| Glycogen synthase kinase-3 alpha |
| **RSQIRsRTPSA** | S301 | SGTA protein | STK6\| Serine/threonine-protein kinase 6 |
| **RSQIRsRTPSA** | S301 | SGTA protein | CLK1\| protein kinase CLK1 |
| **RSQIRsRTPSA** | S301 | SGTA protein | CLK2\| protein kinase CLK2 |
| **RSQIRsRTPSA** | S301 | SGTA protein | ATM\| Serine-protein kinase ATM |
| **RSQIRsRTPSA** | S301 | SGTA protein | \| Serine-protein kinase ATR |
| **RSQIRsRTPSA** | S301 | SGTA protein | \| Serine/threonine-protein kinase PAK 1 |
| **RSQIRsRTPSA** | S301 | SGTA protein | PAK2\| Serine/threonine-protein kinase PAK 2 |
| **RSQIRsRTPSA** | S301 | SGTA protein | PAK3\| Serine/threonine-protein kinase PAK 3 |
| **RSQIRsRTPSA** | S301 | SGTA protein | PRKCD\| Protein kinase C, delta type |
| **RSQIRsRTPSA** | S301 | SGTA protein | PRKCI\| Protein kinase C, iota type |
| **RSQIRsRTPSA** | S301 | SGTA protein | PRKCA\| Protein kinase C, alpha type |
| **RSQIRsRTPSA** | S301 | SGTA protein | PRKCZ\| Protein kinase C, zeta type |
| **RSQIRsRTPSA** | S301 | SGTA protein | PRKCG\| Protein kinase C, gamma type |
| **RSQIRsRTPSA** | S301 | SGTA protein | PRKCQ\| Protein kinase C, theta type |
| **RSQIRsRTPSA** | S301 | SGTA protein | PRKDC\| DNA-dependent protein kinase catalytic subunit |
| **RSRTPsASNDD** | S305 | SGTA protein | PIM2\| Serine/threonine-protein kinase Pim-2 |
| **RSRTPsASNDD** | S305 | SGTA protein | RPS6KB1\| Ribosomal protein S6 kinase 1 |
| **RSRTPsASNDD** | S305 | SGTA protein | AKT1\| RAC-alpha serine/threonine-protein kinase 1 (Protein kinase B) |
| **RSRTPsASNDD** | S305 | SGTA protein | AKT2\| RAC-beta serine/threonine-protein kinase 2 (Protein kinase B) |
| **RSRTPsASNDD** | S305 | SGTA protein | Pim1\| Threonine-protein kinase Pim-1 |
| **KRRPPsPEPST** | S2102 | Spectrin beta chain, brain 1 | CDK2\| Cell division protein kinase 2 |
| **KRRPPsPEPST** | S2102 | Spectrin beta chain, brain 1 | PIM2\| Serine/threonine-protein kinase Pim-2 |
| **KRRPPsPEPST** | S2102 | Spectrin beta chain, brain 1 | MOK\| MAPK/MAK/MRK overlapping kinase |
| **KRRPPsPEPST** | S2102 | Spectrin beta chain, brain 1 | MAPK11\| Mitogen-activated protein kinase p38 beta |
| **KRRPPsPEPST** | S2102 | Spectrin beta chain, brain 1 | MAPK14\| Mitogen-activated protein kinase p38 alpha |
| **KRRPPsPEPST** | S2102 | Spectrin beta chain, brain 1 | MAPK13\| Mitogen-activated protein kinase p38 delta |
| **ATEQRtSSKES** | T2159 | Spectrin beta chain, brain 1 | TGFBR2\| TGF-beta receptor type II |
| **ATEQRtSSKES** | T2159 | Spectrin beta chain, brain 1 | ACTRIIA\| Activin receptor type II |
| **ATEQRtSSKES** | T2159 | Spectrin beta chain, brain 1 | ACVR2B\| Activin receptor type IIB |
| **ATEQRtSSKES** | T2159 | Spectrin beta chain, brain 1 | CK II\| Casein kinase II, alpha and alpha' chain |
| **ATEQRtSSKES** | T2159 | Spectrin beta chain, brain 1 | CAMK2G\| Calcium/calmodulin-dependent protein kinase type II gamma |
| **TSSKEsSPIPS** | S2164 | Spectrin beta chain, brain 1 | TGFBR2\| TGF-beta receptor type II |
| **TSSKEsSPIPS** | S2164 | Spectrin beta chain, brain 1 | ACTRIIA\| Activin receptor type II |
| **TSSKEsSPIPS** | S2164 | Spectrin beta chain, brain 1 | ACVR2B\| Activin receptor type IIB |
| **TSSKEsSPIPS** | S2164 | Spectrin beta chain, brain 1 | NEK2\| Serine/threonine-protein kinase Nek2 |
| **TSSKEsSPIPS** | S2164 | Spectrin beta chain, brain 1 | CSNK1E\| Casein kinase I, epsilon isoform |
| **TSSKEsSPIPS** | S2164 | Spectrin beta chain, brain 1 | CSNK1A1\| Casein kinase I, alpha isoform |
| **TSSKEsSPIPS** | S2164 | Spectrin beta chain, brain 1 | CSNK1D\| Casein kinase I, delta isoform |
| **TSSKEsSPIPS** | S2164 | Spectrin beta chain, brain 1 | STK6\| Serine/threonine-protein kinase 6 |
| **TSSKEsSPIPS** | S2164 | Spectrin beta chain, brain 1 | PRKCD\| Protein kinase C, delta type |
| **TSSKEsSPIPS** | S2164 | Spectrin beta chain, brain 1 | PRKCQ\| Protein kinase C, theta type |
| **TSSKEsSPIPS** | S2164 | Spectrin beta chain, brain 1 | PRKCI\| Protein kinase C, iota type |
| **TSSKEsSPIPS** | S2164 | Spectrin beta chain, brain 1 | PRKCA\| Protein kinase C, alpha type |
| **TSSKEsSPIPS** | S2164 | Spectrin beta chain, brain 1 | PRKCZ\| Protein kinase C, zeta type |
| **TSSKEsSPIPS** | S2164 | Spectrin beta chain, brain 1 | PRKCG\| Protein kinase C, gamma type |
| **SSKESsPIPSP** | S2165 | Spectrin beta chain, brain 1 | CDK2\| Cell division protein kinase 2 |
| **SSKESsPIPSP** | S2165 | Spectrin beta chain, brain 1 | MAPK11\| Mitogen-activated protein kinase p38 beta |
| **SSKESsPIPSP** | S2165 | Spectrin beta chain, brain 1 | MAPK14\| Mitogen-activated protein kinase p38 alpha |
| **SSKESsPIPSP** | S2165 | Spectrin beta chain, brain 1 | MAPK13\| Mitogen-activated protein kinase p38 delta |
| **SSKESsPIPSP** | S2165 | Spectrin beta chain, brain 1 | MAPK8\| c-Jun N-terminal kinase 1 |
| **SSKESsPIPSP** | S2165 | Spectrin beta chain, brain 1 | MAPK10\| c-Jun N-terminal kinase 3 |
| **SSKESsPIPSP** | S2165 | Spectrin beta chain, brain 1 | MAPK9\| c-Jun N-terminal kinase 2 |
| **SSPIPsPTSDR** | S2169 | Spectrin beta chain, brain 1 | CDK2\| Cell division protein kinase 2 |
| **SSPIPsPTSDR** | S2169 | Spectrin beta chain, brain 1 | MAPK11\| Mitogen-activated protein kinase p38 beta |
| **SSPIPsPTSDR** | S2169 | Spectrin beta chain, brain 1 | MAPK14\| Mitogen-activated protein kinase p38 alpha |
| **SSPIPsPTSDR** | S2169 | Spectrin beta chain, brain 1 | MAPK13\| Mitogen-activated protein kinase p38 delta |
| **PSRSSsPQPKV** | S914 | splicing coactivator subunit SRm300 | CDK2\| Cell division protein kinase 2 |
| **PSRSSsPQPKV** | S914 | splicing coactivator subunit SRm300 | MAPK11\| Mitogen-activated protein kinase p38 beta |
| **PSRSSsPQPKV** | S914 | splicing coactivator subunit SRm300 | MAPK14\| Mitogen-activated protein kinase p38 alpha |
| **PSRSSsPQPKV** | S914 | splicing coactivator subunit SRm300 | MAPK13\| Mitogen-activated protein kinase p38 delta |
| **LKSGMsPEQSR** | S1132 | splicing coactivator subunit SRm300 | MAPK11\| Mitogen-activated protein kinase p38 beta |
| **LKSGMsPEQSR** | S1132 | splicing coactivator subunit SRm300 | MAPK14\| Mitogen-activated protein kinase p38 alpha |
| **LKSGMsPEQSR** | S1132 | splicing coactivator subunit SRm300 | MAPK13\| Mitogen-activated protein kinase p38 delta |
| **LKSGMsPEQSR** | S1132 | splicing coactivator subunit SRm300 | CDK2\| Cell division protein kinase 2 |
| **LKSGMsPEQSR** | S1132 | splicing coactivator subunit SRm300 | GSK3B\| Glycogen synthase kinase-3 beta |
| **LKSGMsPEQSR** | S1132 | splicing coactivator subunit SRm300 | GSK3A\| Glycogen synthase kinase-3 alpha |
| **NQSISsPVLDA** | S1404 | splicing coactivator subunit SRm300 | CDK2\| Cell division protein kinase 2 |
| **PPNPEDRsPSPEPIY** | S80 | Splicing factor 1 (Transcription factor ZFM1) | MAPK10\| c-Jun N-terminal kinase 3 |
| **PPNPEDRsPSPEPIY** | S80 | Splicing factor 1 (Transcription factor ZFM1) | MAPK11\| Mitogen-activated protein kinase p38 beta |
| **PPNPEDRsPSPEPIY** | S80 | Splicing factor 1 (Transcription factor ZFM1) | MAPK14\| Mitogen-activated protein kinase p38 alpha |
| **PPNPEDRsPSPEPIY** | S80 | Splicing factor 1 (Transcription factor ZFM1) | MAPK8\| c-Jun N-terminal kinase 1 |
| **PPNPEDRsPSPEPIY** | S80 | Splicing factor 1 (Transcription factor ZFM1) | MAPK9\| c-Jun N-terminal kinase 2 |
| **PPNPEDRsPSPEPIY** | S80 | Splicing factor 1 (Transcription factor ZFM1) | CDC2\| Cell division control protein 2 homolog |
| **PPNPEDRsPSPEPIY** | S80 | Splicing factor 1 (Transcription factor ZFM1) | CDK2\| Cell division protein kinase 2 |
| **PPNPEDRsPSPEPIY** | S80 | Splicing factor 1 (Transcription factor ZFM1) | GSK3B\| Glycogen synthase kinase-3 beta |
| **PPNPEDRsPSPEPIY** | S80 | Splicing factor 1 (Transcription factor ZFM1) | GSK3A\| Glycogen synthase kinase-3 alpha |
| **PPNPEDRsPSPEPIY** | S80 | Splicing factor 1 (Transcription factor ZFM1) | CDK5\| Cell division protein kinase 5 |
| **PPNPEDRsPSPEPIY** | S80 | Splicing factor 1 (Transcription factor ZFM1) | MAPK13\| Mitogen-activated protein kinase p38 delta |
| **PPNPEDRsPSPEPIY** | S80 | Splicing factor 1 (Transcription factor ZFM1) | MAPK12\| Mitogen-activated protein kinase 12 |
| **PPNPEDRsPSPEPIY** | S80 | Splicing factor 1 (Transcription factor ZFM1) | CDK3\| Cell division protein kinase 3 |
| **NPEDRSPsPEPIYNS** | S82 | Splicing factor 1 (Transcription factor ZFM1) | RPS6KA1\| Ribosomal protein S6 kinase alpha 1 |
| **NPEDRSPsPEPIYNS** | S82 | Splicing factor 1 (Transcription factor ZFM1) | MAPK10\| c-Jun N-terminal kinase 3 |
| **NPEDRSPsPEPIYNS** | S82 | Splicing factor 1 (Transcription factor ZFM1) | MAPK11\| Mitogen-activated protein kinase p38 beta |
| **NPEDRSPsPEPIYNS** | S82 | Splicing factor 1 (Transcription factor ZFM1) | MAPK14\| Mitogen-activated protein kinase p38 alpha |
| **NPEDRSPsPEPIYNS** | S82 | Splicing factor 1 (Transcription factor ZFM1) | MAPK8\| c-Jun N-terminal kinase 1 |
| **NPEDRSPsPEPIYNS** | S82 | Splicing factor 1 (Transcription factor ZFM1) | MAPK9\| c-Jun N-terminal kinase 2 |
| **NPEDRSPsPEPIYNS** | S82 | Splicing factor 1 (Transcription factor ZFM1) | RPS6KA3\| Ribosomal protein S6 kinase alpha 3 |
| **NPEDRSPsPEPIYNS** | S82 | Splicing factor 1 (Transcription factor ZFM1) | CDC2\| Cell division control protein 2 homolog |
| **NPEDRSPsPEPIYNS** | S82 | Splicing factor 1 (Transcription factor ZFM1) | CDK2\| Cell division protein kinase 2 |
| **NPEDRSPsPEPIYNS** | S82 | Splicing factor 1 (Transcription factor ZFM1) | GSK3B\| Glycogen synthase kinase-3 beta |
| **NPEDRSPsPEPIYNS** | S82 | Splicing factor 1 (Transcription factor ZFM1) | GSK3A\| Glycogen synthase kinase-3 alpha |
| **NPEDRSPsPEPIYNS** | S82 | Splicing factor 1 (Transcription factor ZFM1) | RPS6KA5\| Ribosomal protein S6 kinase alpha 5 |
| **NPEDRSPsPEPIYNS** | S82 | Splicing factor 1 (Transcription factor ZFM1) | RPS6KB1\| Ribosomal protein S6 kinase 1 |
| **NPEDRSPsPEPIYNS** | S82 | Splicing factor 1 (Transcription factor ZFM1) | CDK5\| Cell division protein kinase 5 |
| **NPEDRSPsPEPIYNS** | S82 | Splicing factor 1 (Transcription factor ZFM1) | MAPK13\| Mitogen-activated protein kinase p38 delta |
| **NPEDRSPsPEPIYNS** | S82 | Splicing factor 1 (Transcription factor ZFM1) | RPS6KA4\| Ribosomal protein S6 kinase alpha 4 |
| **NPEDRSPsPEPIYNS** | S82 | Splicing factor 1 (Transcription factor ZFM1) | RPS6KA2\| Ribosomal protein S6 kinase alpha 2 |
| **NPEDRSPsPEPIYNS** | S82 | Splicing factor 1 (Transcription factor ZFM1) | MAPK12\| Mitogen-activated protein kinase 12 |
| **NPEDRSPsPEPIYNS** | S82 | Splicing factor 1 (Transcription factor ZFM1) | CDK3\| Cell division protein kinase 3 |
| **NPEDRSPsPEPIYNS** | S82 | Splicing factor 1 (Transcription factor ZFM1) | RPS6KB2\| Ribosomal protein S6 kinase 2 |
| **RPDPDsDEDED** | S155 | Splicing factor 45 | CK II\| Casein kinase II, alpha and alpha' chain |
| **PIETGsPKTKE** | S449 | Splicing factor arginine/serine-rich 11 | CDK2\| Cell division protein kinase 2 |
| **PSKARsVSPPP** | S314 | Splicing factor, arginine/serine-rich 6 | PAK4\| Serine/threonine-protein kinase PAK 4 |
| **PSKARsVSPPP** | S314 | Splicing factor, arginine/serine-rich 6 | PAK7\| Serine/threonine-protein kinase PAK 7 |
| **PSKARsVSPPP** | S314 | Splicing factor, arginine/serine-rich 6 | PAK2\| Serine/threonine-protein kinase PAK 2 |
| **PSKARsVSPPP** | S314 | Splicing factor, arginine/serine-rich 6 | \| Serine/threonine-protein kinase PAK 1 |
| **PSKARsVSPPP** | S314 | Splicing factor, arginine/serine-rich 6 | PAK3\| Serine/threonine-protein kinase PAK 3 |
| **KARSVsPPPKR** | S316 | Splicing factor, arginine/serine-rich 6 | CDK2\| Cell division protein kinase 2 |
| **KARSVsPPPKR** | S316 | Splicing factor, arginine/serine-rich 6 | PIM2\| Serine/threonine-protein kinase Pim-2 |
| **KARSVsPPPKR** | S316 | Splicing factor, arginine/serine-rich 6 | MAPK11\| Mitogen-activated protein kinase p38 beta |
| **KARSVsPPPKR** | S316 | Splicing factor, arginine/serine-rich 6 | MAPK14\| Mitogen-activated protein kinase p38 alpha |
| **KARSVsPPPKR** | S316 | Splicing factor, arginine/serine-rich 6 | MAPK13\| Mitogen-activated protein kinase p38 delta |
| **KARSVsPPPKR** | S316 | Splicing factor, arginine/serine-rich 6 | MAPK8\| c-Jun N-terminal kinase 1 |
| **KARSVsPPPKR** | S316 | Splicing factor, arginine/serine-rich 6 | MAPK10\| c-Jun N-terminal kinase 3 |
| **KARSVsPPPKR** | S316 | Splicing factor, arginine/serine-rich 6 | MAPK9\| c-Jun N-terminal kinase 2 |
| **GSPRRsASPER** | S231 | Splicing factor, arginine/serine-rich 7 | AURKB\| Serine/threonine-protein kinase 12 |
| **GSPRRsASPER** | S231 | Splicing factor, arginine/serine-rich 7 | PAK4\| Serine/threonine-protein kinase PAK 4 |
| **GSPRRsASPER** | S231 | Splicing factor, arginine/serine-rich 7 | PAK7\| Serine/threonine-protein kinase PAK 7 |
| **GSPRRsASPER** | S231 | Splicing factor, arginine/serine-rich 7 | \| Serine/threonine-protein kinase PAK 1 |
| **GSPRRsASPER** | S231 | Splicing factor, arginine/serine-rich 7 | PAK2\| Serine/threonine-protein kinase PAK 2 |
| **GSPRRsASPER** | S231 | Splicing factor, arginine/serine-rich 7 | PAK3\| Serine/threonine-protein kinase PAK 3 |
| **GSPRRsASPER** | S231 | Splicing factor, arginine/serine-rich 7 | STK6\| Serine/threonine-protein kinase 6 |
| **GSPRRsASPER** | S231 | Splicing factor, arginine/serine-rich 7 | DMPK\| Myotonin-protein kinase |
| **PRRSAsPERMD** | S233 | Splicing factor, arginine/serine-rich 7 | MOK\| MAPK/MAK/MRK overlapping kinase |
| **PRRSAsPERMD** | S233 | Splicing factor, arginine/serine-rich 7 | MAPK11\| Mitogen-activated protein kinase p38 beta |
| **PRRSAsPERMD** | S233 | Splicing factor, arginine/serine-rich 7 | MAPK14\| Mitogen-activated protein kinase p38 alpha |
| **PRRSAsPERMD** | S233 | Splicing factor, arginine/serine-rich 7 | MAPK13\| Mitogen-activated protein kinase p38 delta |
| **PRRSAsPERMD** | S233 | Splicing factor, arginine/serine-rich 7 | MAPK8\| c-Jun N-terminal kinase 1 |
| **PRRSAsPERMD** | S233 | Splicing factor, arginine/serine-rich 7 | MAPK10\| c-Jun N-terminal kinase 3 |
| **PRRSAsPERMD** | S233 | Splicing factor, arginine/serine-rich 7 | MAPK9\| c-Jun N-terminal kinase 2 |
| **PRRSAsPERMD** | S233 | Splicing factor, arginine/serine-rich 7 | CLK1\| protein kinase CLK1 |
| **DEYADsDEDQH** | S444 | Structure-specific recognition protein 1 | CK II\| Casein kinase II, alpha and alpha' chain |
| **GMNPSyDEYAD** | Y438 | Structure-specific recognition protein 1 | IGF1R\| Insulin-like growth factor 1 receptor |
| **GMNPSyDEYAD** | Y438 | Structure-specific recognition protein 1 | INSR\| Insulin receptor |
| **PSYDEyADSDE** | Y441 | Structure-specific recognition protein 1 | EPHA4\| Ephrin type-A receptor 4 |
| **PSYDEyADSDE** | Y441 | Structure-specific recognition protein 1 | EPHA7\| Ephrin type-A receptor 7 |
| **PSYDEyADSDE** | Y441 | Structure-specific recognition protein 1 | EPHA3\| Ephrin type-A receptor 3 |
| **PSYDEyADSDE** | Y441 | Structure-specific recognition protein 1 | IGF1R\| Insulin-like growth factor 1 receptor |
| **PSYDEyADSDE** | Y441 | Structure-specific recognition protein 1 | INSR\| Insulin receptor |
| **KKRKRsPSPSP** | S302 | SWI/SNF complex 170 kDa subunit | CDK2\| Cell division protein kinase 2 |
| **KKRKRsPSPSP** | S302 | SWI/SNF complex 170 kDa subunit | PIM2\| Serine/threonine-protein kinase Pim-2 |
| **KKRKRsPSPSP** | S302 | SWI/SNF complex 170 kDa subunit | RPS6KB1\| Ribosomal protein S6 kinase 1 |
| **RKRSPsPSPTP** | S304 | SWI/SNF complex 170 kDa subunit | PIM2\| Serine/threonine-protein kinase Pim-2 |
| **RKRSPsPSPTP** | S304 | SWI/SNF complex 170 kDa subunit | RPS6KB1\| Ribosomal protein S6 kinase 1 |
| **RSPSPsPTPEA** | S306 | SWI/SNF complex 170 kDa subunit | CDK2\| Cell division protein kinase 2 |
| **RSPSPsPTPEA** | S306 | SWI/SNF complex 170 kDa subunit | MAPK11\| Mitogen-activated protein kinase p38 beta |
| **RSPSPsPTPEA** | S306 | SWI/SNF complex 170 kDa subunit | MAPK14\| Mitogen-activated protein kinase p38 alpha |
| **RSPSPsPTPEA** | S306 | SWI/SNF complex 170 kDa subunit | MAPK13\| Mitogen-activated protein kinase p38 delta |
| **RSPSPsPTPEA** | S306 | SWI/SNF complex 170 kDa subunit | CLK1\| protein kinase CLK1 |
| **RSPSPsPTPEA** | S306 | SWI/SNF complex 170 kDa subunit | CLK2\| protein kinase CLK2 |
| **DWEDDsDEDMS** | S113 | Telomerase-binding protein p23 | CK II\| Casein kinase II, alpha and alpha' chain |
| **DEEDVsEEEAE** | S247 | Thioredoxin domain containing protein 1 precursor | CK II\| Casein kinase II, alpha and alpha' chain |
| **KDQQPsGSEGE** | S86 | THUMP domain containing protein 1 | CK II\| Casein kinase II, alpha and alpha' chain |
| **QQPSGsEGEDD** | S88 | THUMP domain containing protein 1 | CK II\| Casein kinase II, alpha and alpha' chain |
| **AVSELsPRERS** | S243 | TRAP150 protein | CDK2\| Cell division protein kinase 2 |
| **AVSELsPRERS** | S243 | TRAP150 protein | MAPK11\| Mitogen-activated protein kinase p38 beta |
| **AVSELsPRERS** | S243 | TRAP150 protein | MAPK14\| Mitogen-activated protein kinase p38 alpha |
| **AVSELsPRERS** | S243 | TRAP150 protein | MAPK13\| Mitogen-activated protein kinase p38 delta |
| **GVDEQsDSSEE** | S305 | Transcription initiation factor IIF alpha subunit | CK II\| Casein kinase II, alpha and alpha' chain |
| **DEQSDsSEESE** | S307 | Transcription initiation factor IIF alpha subunit | CK II\| Casein kinase II, alpha and alpha' chain |
| **EQSDSsEESEE** | S308 | Transcription initiation factor IIF alpha subunit | CK II\| Casein kinase II, alpha and alpha' chain |
| **DSSEEsEEEKP** | S311 | Transcription initiation factor IIF alpha subunit | CK II\| Casein kinase II, alpha and alpha' chain |
| **SAAASsPAGGG** | S50 | Transcription intermediary factor 1-beta | CDK2\| Cell division protein kinase 2 |
| **SAPQMsPGSSD** | S467 | Ubiquitin associated protein 2-like | MAPK11\| Mitogen-activated protein kinase p38 beta |
| **SAPQMsPGSSD** | S467 | Ubiquitin associated protein 2-like | MAPK14\| Mitogen-activated protein kinase p38 alpha |
| **SAPQMsPGSSD** | S467 | Ubiquitin associated protein 2-like | MAPK13\| Mitogen-activated protein kinase p38 delta |
| **SAPQMsPGSSD** | S467 | Ubiquitin associated protein 2-like | CDK2\| Cell division protein kinase 2 |
| **SAPQMsPGSSD** | S467 | Ubiquitin associated protein 2-like | MOK\| MAPK/MAK/MRK overlapping kinase |
| **SAPQMsPGSSD** | S467 | Ubiquitin associated protein 2-like | MAPK1\| Mitogen-activated protein kinase 1 |
| **SAPQMsPGSSD** | S467 | Ubiquitin associated protein 2-like | MAPK3\| Mitogen-activated protein kinase 3 |
| **SAPQMsPGSSD** | S467 | Ubiquitin associated protein 2-like | MAPK7\| Mitogen-activated protein kinase 7 |
| **SSDNQsSSPQP** | S475 | Ubiquitin associated protein 2-like | TGFBR2\| TGF-beta receptor type II |
| **SSDNQsSSPQP** | S475 | Ubiquitin associated protein 2-like | ACTRIIA\| Activin receptor type II |
| **SLYASsPGGVY** | S56 | Vimentin | CDK2\| Cell division protein kinase 2 |
| **VINETsQHHDD** | S459 | Vimentin | ATM\| Serine-protein kinase ATM |
| **VINETsQHHDD** | S459 | Vimentin | ATR\| Serine-protein kinase ATR |
| **WLRDPsASPGD** | S288 | Vinculin | CAMK2A\| Calcium/calmodulin-dependent protein kinase type II alpha chain |
| **WLRDPsASPGD** | S288 | Vinculin | DMPK\| Myotonin-protein kinase |
| **WLRDPsASPGD** | S288 | Vinculin | PRKG1\| cGMP-dependent protein kinase 1, beta isozyme |
| **WLRDPsASPGD** | S288 | Vinculin | TGFBR2\| TGF-beta receptor type II |
| **VEDKEsEGEEE** | S153 | Zinc finger protein 265 | CK II\| Casein kinase II, alpha and alpha' chain |

**Additional File 3. Phosphorylated sites found in peptides according to MS experiments and kinase phosphorylation analysis using NetworKIN 2.0 Beta (http://www.networkin.info/version_2_0/).** To investigate which kinases could be involved in the phosphorylation of peptides found in MS experiments, NetworKIN 2.0 Beta kinase databank was used to match phoshorylated serine, threonine and tyrosine in peptides found to be phoshorylated, according to prior experiments in literature for a given kinase based on PhosphoELM (http://phospho.elm.eu.org/) and on Phosphosite (http://www.phosphosite.org).
