# Supplementary material for: New insights in osteogenic differentiation revealed by mass spectrometric assessment of phosphorylated substrates in murine skin mesenchymal cells
Source: BMC Cell Biol. 2013 Oct 22;14:47. doi: 10.1186/1471-2121-14-47 (PMC3819743; doi:10.1186/1471-2121-14-47)
Supplement: Additional file 4 — Network of proteins which were found to interact with phosphorylated proteins found. The list of phosphoproteins found were subjected to Ingenuity Pathway Analysis (IPA) to investigate probable protein interactions for each cellular compartment. Proteins described to be transcription factors were selected to investigate the activation of osteoblast related genes by quantitative real-time PCR. [file 1471-2121-14-47-S4.docx]

| Additional File 4. Ingenuity® Protein network analysis for the phosphoproteins found in MS experiments | | |
| --- | --- | --- |
| Symbol | Entrez Gene Name | Location |
| ACLY | ATP Citrate Lyase | Cytoplasm |
| ACSL1 | Acyl-Coa Synthetase Long-Chain Family Member 1 | Cytoplasm |
| AKAP12 | A Kinase (PRKA) Anchor Protein 12 | Cytoplasm |
| BCL2 | B-Cell CLL/Lymphoma 2 | Cytoplasm |
| BMF | Bcl2 Modifying Factor | Cytoplasm |
| Bnip3 | BCL2/Adenovirus E1B Interacting Protein 3 | Cytoplasm |
| BNIP3L | BCL2/Adenovirus E1B 19kda Interacting Protein 3-Like | Cytoplasm |
| CANX | Calnexin | Cytoplasm |
| CASP4 | Caspase 4, Apoptosis-Related Cysteine Peptidase | Cytoplasm |
| Ces2b/Ces2c | Carboxylesterase 2C | Cytoplasm |
| CIDEC | Cell Death-Inducing DFFA-Like Effector C | Cytoplasm |
| CRAT | Carnitine O-Acetyltransferase | Cytoplasm |
| CYP24A1 | Cytochrome P450, Family 24, Subfamily A, Polypeptide 1 | Cytoplasm |
| CYP51A1 | Cytochrome P450, Family 51, Subfamily A, Polypeptide 1 | Cytoplasm |
| CYP7B1 | Cytochrome P450, Family 7, Subfamily B, Polypeptide 1 | Cytoplasm |
| DVL2 | Dishevelled, Dsh Homolog 2 (Drosophila) | Cytoplasm |
| DVL3 | Dishevelled, Dsh Homolog 3 (Drosophila) | Cytoplasm |
| FHL1 | Four And A Half LIM Domains 1 | Cytoplasm |
| GPX2 | Glutathione Peroxidase 2 (Gastrointestinal) | Cytoplasm |
| GSTA5 | Glutathione S-Transferase Alpha 5 | Cytoplasm |
| HSP90AB1 | Heat Shock Protein 90kda Alpha (Cytosolic), Class B Member 1 | Cytoplasm |
| IQGAP1 | IQ Motif Containing Gtpase Activating Protein 1 | Cytoplasm |
| LIMA1 | LIM Domain And Actin Binding 1 | Cytoplasm |
| MAP1B | Microtubule-Associated Protein 1B | Cytoplasm |
| MAP4 | Microtubule-Associated Protein 4 | Cytoplasm |
| MARCKSL1 | MARCKS-Like 1 | Cytoplasm |
| MYH9 | Myosin, Heavy Chain 9, Non-Muscle | Cytoplasm |
| NEDD4 | Neural Precursor Cell Expressed, Developmentally Down-Regulated 4 | Cytoplasm |
| NMT1 | N-Myristoyltransferase 1 | Cytoplasm |
| PALLD | Palladin, Cytoskeletal Associated Protein | Cytoplasm |
| PIK3R3 | Phosphoinositide-3-Kinase, Regulatory Subunit 3 (Gamma) | Cytoplasm |
| PLA2G4A | Phospholipase A2, Group IVA (Cytosolic, Calcium-Dependent) | Cytoplasm |
| PLEC | Plectin | Cytoplasm |
| PLS3 | Plastin 3 | Cytoplasm |
| PPP2CA | Protein Phosphatase 2, Catalytic Subunit, Alpha Isozyme | Cytoplasm |
| PPP2R2B | Protein Phosphatase 2, Regulatory Subunit B, Beta | Cytoplasm |
| RPLP0 | Ribosomal Protein, Large, P0 | Cytoplasm |
| RPLP2 | Ribosomal Protein, Large, P2 | Cytoplasm |
| SH3KBP1 | SH3-Domain Kinase Binding Protein 1 | Cytoplasm |
| TNFAIP8 | Tumor Necrosis Factor, Alpha-Induced Protein 8 | Cytoplasm |
| TSPO | Translocator Protein (18kda) | Cytoplasm |
| VIM | Vimentin | Cytoplasm |
| AFP | Alpha-Fetoprotein | Extracellular Space |
| CLCF1 | Cardiotrophin-Like Cytokine Factor 1 | Extracellular Space |
| LGALS3 | Lectin, Galactoside-Binding, Soluble, 3 | Extracellular Space |
| LGALS8 | Lectin, Galactoside-Binding, Soluble, 8 | Extracellular Space |
| RELN | Reelin | Extracellular Space |
| SERPINA1 | Serpin Peptidase Inhibitor, Clade A Member 1 | Extracellular Space |
| TNC | Tenascin C | Extracellular Space |
| TNFSF14 | Tumor Necrosis Factor (Ligand) Superfamily, Member 14 | Extracellular Space |
| WNT4 | Wingless-Type MMTV Integration Site Family, Member 4 | Extracellular Space |
| WNT5A | Wingless-Type MMTV Integration Site Family, Member 5A | Extracellular Space |
| AHNAK | AHNAK Nucleoprotein | Nucleus |
| Ap1 | Transcription factor AP-1 | Nucleus |
| BARD1 | BRCA1 Associated RING Domain 1 | Nucleus |
| BIN1 | Bridging Integrator 1 | Nucleus |
| BMI1 | BMI1 Polycomb Ring Finger Oncogene | Nucleus |
| CBX3 | Chromobox Homolog 3 | Nucleus |
| CBX4 | Chromobox Homolog 4 | Nucleus |
| CCND1 | Cyclin D1 | Nucleus |
| CDC6 | Cell Division Cycle 6 Homolog (S. Cerevisiae) | Nucleus |
| CDCA7L | Cell Division Cycle Associated 7-Like | Nucleus |
| CTNNB1 | Catenin (Cadherin-Associated Protein), Beta 1, 88kda | Nucleus |
| DNMT1 | DNA (Cytosine-5-)-Methyltransferase 1 | Nucleus |
| DUSP16 | Dual Specificity Phosphatase 16 | Nucleus |
| E2F4 | E2F Transcription Factor 4, P107/P130-Binding | Nucleus |
| ECT2 | Epithelial Cell Transforming Sequence 2 Oncogene | Nucleus |
| HIC1 | Hypermethylated In Cancer 1 | Nucleus |
| Histone h3 | Histone H3 | Nucleus |
| HMGA1 | High Mobility Group AT-Hook 1 | Nucleus |
| HMGA2 | High Mobility Group AT-Hook 2 | Nucleus |
| HNF4A | Hepatocyte Nuclear Factor 4, Alpha | Nucleus |
| HNRNPK | Heterogeneous Nuclear Ribonucleoprotein K | Nucleus |
| ID3 | Inhibitor Of DNA Binding 3, Dominant Negative Helix-Loop-Helix Protein | Nucleus |
| KDM5B | Lysine (K)-Specific Demethylase 5B | Nucleus |
| KPNA3 | Karyopherin Alpha 3 (Importin Alpha 4) | Nucleus |
| LMNA | Lamin A/C | Nucleus |
| MAD2L1 | MAD2 Mitotic Arrest Deficient-Like 1 (Yeast) | Nucleus |
| MAZ | MYC-Associated Zinc Finger Protein (Purine-Binding Transcription Factor) | Nucleus |
| MCM2 | Minichromosome Maintenance Complex Component 2 | Nucleus |
| MCM3 | Minichromosome Maintenance Complex Component 3 | Nucleus |
| MCM5 | Minichromosome Maintenance Complex Component 5 | Nucleus |
| MTA1 | Metastasis Associated 1 | Nucleus |
| MYC | V-Myc Myelocytomatosis Viral Oncogene Homolog (Avian) | Nucleus |
| NAA10 | N(Alpha)-Acetyltransferase 10, Nata Catalytic Subunit | Nucleus |
| Ncl | Nucleolin | Nucleus |
| NDC80 | NDC80 Homolog, Kinetochore Complex Component (S. Cerevisiae) | Nucleus |
| NFATC1 | Nuclear Factor Of Activated T-Cells, Cytoplasmic, Calcineurin-Dependent 1 | Nucleus |
| NFKB1 | Nuclear Factor Of Kappa Light Polypeptide Gene Enhancer In B-Cells 1 | Nucleus |
| NOLC1 | Nucleolar And Coiled-Body Phosphoprotein 1 | Nucleus |
| NR1I3 | Nuclear Receptor Subfamily 1, Group I, Member 3 | Nucleus |
| NR3C1 | Nuclear Receptor Subfamily 3, Group C, Member 1 (Glucocorticoid Receptor) | Nucleus |
| PPP1R13L | Protein Phosphatase 1, Regulatory (Inhibitor) Subunit 13 Like | Nucleus |
| PRMT1 | Protein Arginine Methyltransferase 1 | Nucleus |
| PSIP1 | PC4 And SFRS1 Interacting Protein 1 | Nucleus |
| PTBP1 | Polypyrimidine Tract Binding Protein 1 | Nucleus |
| SIAH2 | Seven In Absentia Homolog 2 (Drosophila) | Nucleus |
| SMAD3 | SMAD Family Member 3 | Nucleus |
| SMN1/SMN2 | Survival Of Motor Neuron 1, Telomeric | Nucleus |
| SOX4 | SRY (Sex Determining Region Y)-Box 4 | Nucleus |
| SP1 | Sp1 Transcription Factor | Nucleus |
| SREBF1 | Sterol Regulatory Element Binding Transcription Factor 1 | Nucleus |
| TCOF1 | Treacher Collins-Franceschetti Syndrome 1 | Nucleus |
| TFDP1 | Transcription Factor Dp-1 | Nucleus |
| TGFB1I1 | Transforming Growth Factor Beta 1 Induced Transcript 1 | Nucleus |
| TLE1 | Transducin-Like Enhancer Of Split 1 (E(Sp1) Homolog, Drosophila) | Nucleus |
| TMPO | Thymopoietin | Nucleus |
| TOP2A | Topoisomerase (DNA) II Alpha 170kda | Nucleus |
| TP53BP2 | Tumor Protein P53 Binding Protein, 2 | Nucleus |
| YAP1 | Yes-Associated Protein 1 | Nucleus |
| ACVR2A | Activin A Receptor, Type IIA | Plasma Membrane |
| AGTR1 | Angiotensin II Receptor, Type 1 | Plasma Membrane |
| AMIGO2 | Adhesion Molecule With Ig-Like Domain 2 | Plasma Membrane |
| Ap2 alpha | Transcription factor AP-2-alpha | Plasma Membrane |
| Cadherin | Cadherin | Plasma Membrane |
| Cald1 | Caldesmon 1 | Plasma Membrane |
| CFTR | ATP-Binding Cassette Sub-Family C, Member 7 | Plasma Membrane |
| CLNS1A | Chloride Channel, Nucleotide-Sensitive, 1A | Plasma Membrane |
| DAB2 | Disabled Homolog 2, Mitogen-Responsive Phosphoprotein (Drosophila) | Plasma Membrane |
| DAG1 | Dystroglycan 1 (Dystrophin-Associated Glycoprotein 1) | Plasma Membrane |
| EPB41L1 | Erythrocyte Membrane Protein Band 4.1-Like 1 | Plasma Membrane |
| EZR | Ezrin | Plasma Membrane |
| F2R | Coagulation Factor II (Thrombin) Receptor | Plasma Membrane |
| HMMR | Hyaluronan-Mediated Motility Receptor (RHAMM) | Plasma Membrane |
| IL15RA | Interleukin 15 Receptor, Alpha | Plasma Membrane |
| IL2RG | Interleukin 2 Receptor, Gamma | Plasma Membrane |
| IL7R | Interleukin 7 Receptor | Plasma Membrane |
| ITGA2 | Integrin, Alpha 2 (CD49B, Alpha 2 Subunit Of VLA-2 Receptor) | Plasma Membrane |
| JAM2 | Junctional Adhesion Molecule 2 | Plasma Membrane |
| LRP2 | Low Density Lipoprotein Receptor-Related Protein 2 | Plasma Membrane |
| MARCKS | Myristoylated Alanine-Rich Protein Kinase C Substrate | Plasma Membrane |
| PMP22 | Peripheral Myelin Protein 22 | Plasma Membrane |
| PTH1R | Parathyroid Hormone 1 Receptor | Plasma Membrane |
| SCNN1A | Sodium Channel, Nonvoltage-Gated 1 Alpha | Plasma Membrane |
| SFRP1 | Secreted Frizzled-Related Protein 1 | Plasma Membrane |
| SLC9A3R1 | Solute Carrier Family 9 (Sodium/Hydrogen Exchanger), Member 3 Regulator 1 | Plasma Membrane |
| SLC9A3R2 | Solute Carrier Family 9 (Sodium/Hydrogen Exchanger), Member 3 Regulator 2 | Plasma Membrane |
| let-7 | Microrna Let-7b | unknown |
| Thymidine Kinase | Thymidine Kinase | unknown |

**Additional File 4. Network of proteins which were found to interact with phosphorylated proteins found.** The list of phosphoproteins found were subjected to Ingenuity Pathway Analysis (IPA) to investigate problable protein interactions for each cellular compartment. Proteins described to be transcription factors were selected to investigate the activation of osteoblast related genes by quantitative real-time PCR.
