# Supplementary material for: New insights in osteogenic differentiation revealed by mass spectrometric assessment of phosphorylated substrates in murine skin mesenchymal cells
Source: BMC Cell Biol. 2013 Oct 22;14:47. doi: 10.1186/1471-2121-14-47 (PMC3819743; doi:10.1186/1471-2121-14-47)
Supplement: Additional file 5 — Predicted network of interaction for phosphoproteins found using Ingenuity Pathway Analysis. Ingenuity Pathway Analysis was used to identify the network of proteins which could interact with the phosphoproteins identified. Dashed arrows represent the predicted interactions and the full arrows represent a confirmed interaction. Proteins were separated by cell compartment and proteins known to be transcription factors were selected to further analysis to investigate possible activators of osteoblast differentiation by real-time quantitative PCR. [file 1471-2121-14-47-S5.docx]

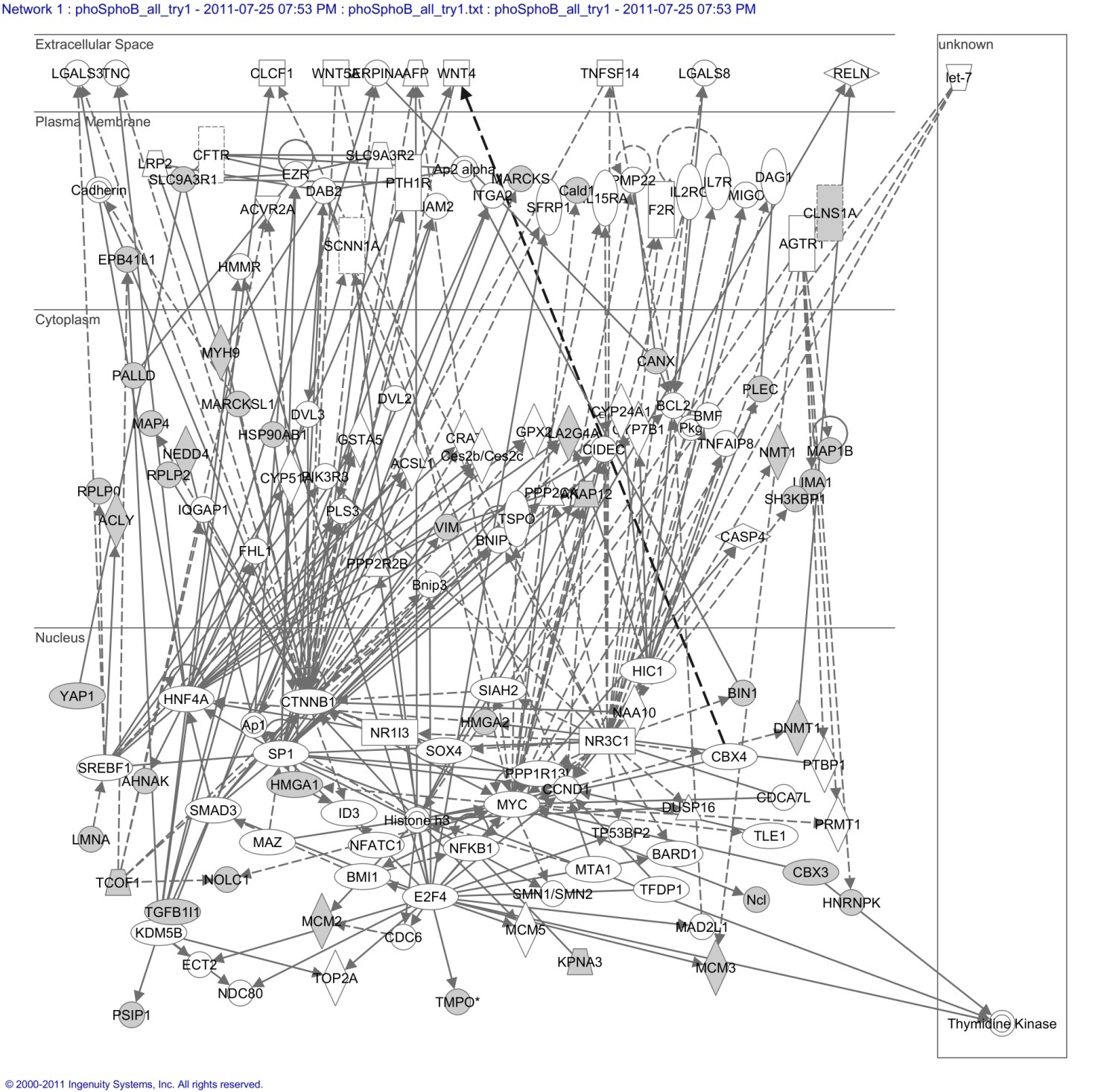


**Additional File 5. Predicted network of interaction for phosphoproteins found using Ingenuity Pathway Analysis.** Ingenuity Pathway Analysis was used to identify the network of proteins which could interact with the phosphoproteins identified. Dashed arrows represent the predicted interactions and the full arrows represent a confirmed interaction. Proteins were separated by cell compartiment and proteins known to be transcription factors were selected to further analysis to investigate possible activators of osteoblast differentiation by real-time quantitative PCR.
